# Root Exudation of Phytochemicals in Arabidopsis Follows Specific Patterns That Are Developmentally Programmed and Correlate with Soil Microbial Functions

**DOI:** 10.1371/journal.pone.0055731

**Published:** 2013-02-01

**Authors:** Jacqueline M. Chaparro, Dayakar V. Badri, Matthew G. Bakker, Akifumi Sugiyama, Daniel K. Manter, Jorge M. Vivanco

**Affiliations:** 1 Department of Horticulture and Landscape Architecture, Colorado State University, Fort Collins, Colorado, United States of America; 2 Research Institute for Sustainable Humanosphere, Kyoto University, Uji, Kyoto, Japan; 3 United States Department of Agriculture - Agricultural Research Service, Soil-Plant-Nutrient Research Unit, Fort Collins, Colorado, United States of America; National Taiwan University, Taiwan

## Abstract

Plant roots constantly secrete compounds into the soil to interact with neighboring organisms presumably to gain certain functional advantages at different stages of development. Accordingly, it has been hypothesized that the phytochemical composition present in the root exudates changes over the course of the lifespan of a plant. Here, root exudates of *in vitro* grown *Arabidopsis* plants were collected at different developmental stages and analyzed using GC-MS. Principle component analysis revealed that the composition of root exudates varied at each developmental stage. Cumulative secretion levels of sugars and sugar alcohols were higher in early time points and decreased through development. In contrast, the cumulative secretion levels of amino acids and phenolics increased over time. The expression in roots of genes involved in biosynthesis and transportation of compounds represented in the root exudates were consistent with patterns of root exudation. Correlation analyses were performed of the *in vitro* root exudation patterns with the functional capacity of the rhizosphere microbiome to metabolize these compounds at different developmental stages of Arabidopsis grown in natural soils. Pyrosequencing of rhizosphere mRNA revealed strong correlations (p<0.05) between microbial functional genes involved in the metabolism of carbohydrates, amino acids and secondary metabolites with the corresponding compounds released by the roots at particular stages of plant development. In summary, our results suggest that the root exudation process of phytochemicals follows a developmental pattern that is genetically programmed.

## Introduction

Plants use root exudates as chemical cues to monitor and interact with their surroundings [1], [2]. Exudate release is dependent on the needs of a plant [3] and exudation can be modified in order to cope with stresses [4], [5]. For example, to overcome nitrogen deficiency legumes release specific flavonols which attract and initiate symbiotic relationships with rhizobia [6]. However, when N fertilization is supplemented the symbiotic interaction is halted [7]. When *Arabidopsis* is attacked by the foliar pathogen *Pseudomonas syringae* pv *tomato*, roots release malic acid which recruits beneficial soil bacteria capable of triggering host defense responses against *P. syringae* [8]. *Zea mays* releases 2,4-dihydroxy-7-methoxy-1,4-benzoxazin-3-one that chemotactically attracts the beneficial rhizobacterium *Pseudomonas putida* KT2440 [9]. Besides such one-to-one interactions, multitrophic interactions also occur in the rhizosphere. For instance, *Medicago truncatula* emits dimethyl sulfide that attracts *Caenorhabditis elegans*, which in turn transports rhizobia close to the legume roots to initiate symbiosis [10]. Evidence is mounting that the cross talk between plants and the soil microbes is largely orchestrated by root exudates, not only at the one compound-one microbe level, but at the community level. For instance, it has been reported that changes in root exudation due to mutation of an ABC transporter gene modulated the soil microbial community composition such that more beneficial microbes were cultured [11]. Similarly, Micallef et al. [12] showed that soil microbial communities are affected by plant age and genotype.

Rhizosphere microbial communities have shown strong ties to root exudate composition [13] and changes in exudate composition result in significant modifications of the soil microbial community [11]. Root exudate composition and concentrations change depending upon the environment in which a plant is growing, including soil edaphic and biological factors [14]–[17]. It has been previously reported that root secretion of proteins changes with plant development, and when challenged by pathogens or symbiotic bacteria [1], [2], [16], [17]. Root secretions of some phytochemicals have also been shown to follow a diurnal rhythm [18]. However, there is no information available on how the composition of root secreted primary and secondary metabolites changes over the course of plant development, and how those changes correlate to the functioning of the rhizosphere microbiome.

Soil microbes have been shown to have both negative and positive effects on plant development. For example, *Agrobacterium rhizogenes* influence and manipulate plant development for the formation of hairy roots [19], [20]. Similarly, soil microbes such as PGPRs can modulate plant growth through the production of hormones such as auxin and cytokinin or via the release of volatile organic compounds [20].

The rhizomicrobiome plays an important role in disease suppression by direct antagonism against pathogens [21], in overcoming abiotic stress by induced systemic tolerance [4] and in overcoming biotic stress by increasing the plant's innate immunity [5]. It has also been documented that phytohormone production and enhanced access to nutrients due to rhizomicrobiome activity has a positive correlation with plant productivity [22] and overall plant health [23], [24].

In summary, there is a concerted understanding of the ability of root exudates to influence the structure of rhizosphere microbial communities. Root exudates act as substrates, signals and/or antimicrobials influencing the relative abundance of microbial taxa in the rhizosphere. However, the functional capacity of most of these organisms is unknown and our understanding of the correlation between root exudation and microbiome functioning remains limited. Here, we show how *in vitro Arabidopsis* root exudate composition changes over the course of plant development, and we correlate these patterns with the ability of the soil microbiome to metabolize those compounds under natural soil conditions.

## Materials and Methods

### Plant growth conditions and root exudate collection

*Arabidopsis* wild type (Col-0) plants were grown and root exudates were collected by using an established protocol as previously described [1], [2], [11], [15], [18], [25]–[27], with a few modifications. *Arabidopsis* wild type (Col-0) seeds were surface-sterilized with Clorox® for one minute followed by four rinses in sterile distilled water and plated on Murashige and Skoog medium (MS) [28], supplemented with 3% sucrose and 0.9% bactoagar in Petri plates. Petri plates were incubated in a growth chamber (Percival Scientific) at 25°C for seven days, with a photoperiod of 16 h light/8 h dark. To collect root exudates at different developmental time points, seven-day-old seedlings were transferred to Magenta® boxes each containing 10 ml of liquid MS (MS basal salts supplemented with 1% sucrose), incubated on an orbital shaker at 90 rpm and illuminated under cool white fluorescent light (45 µmol m^−2^ s^−1^) with a photoperiod of 16 h light/8 h dark at 25°C±2. Prior to exudate collection (7, 14, 21 or 28 days), plants were gently washed with sterile water to remove the surface-adhering exudates and transferred to new Magenta® boxes containing 10 ml of sterile water. Growth medium plus dissolved exudates were collected at approximately the same time on the third day, after three days of continuous secretion for each time point (7–10, 14–17, 21–24 and 28–31 days). Each growth stage of the plant was as follows: the 10 day plants consisted of the two leaf growth stage, the 17 day plants were at the 5 leaf rosette stage, the 24 day plants reached the bolting stage and the 31 day plants reached the flowering stage as described by Boyes et al. [29], representative pictures of each growth stage can be found in De-la-Pena et al. [1]. Each time point consisted of three replicates and each replicate consisted of a total volume of 180 ml of exudate-containing medium, from 18 individually-grown *Arabidopsis* plants. The collected root exudates were filtered using nylon filters of pore size 0.45 µm (Millipore, MA) to remove root sheathing and root-border-like cells. After filtration, the exudates were freeze-dried (Labconco, MO) and stored at −20°C for further analyses. Plant root tissues were collected from each replicate of all time points, frozen with liquid nitrogen and stored at −80°C for gene expression analyses. Sterile techniques were used throughout the experiment and there was no evidence of contamination in the media.

### Gas Chromatography and Mass Spectrometry (GC-MS) analyses of exudates

Freeze dried root exudates were dissolved in 5 ml of 80% methanol and the supernatant was collected into new glass tubes after centrifugation at 8000 rpm for 15 minutes at room temperature. The supernatants were dried under nitrogen gas and shipped to the Genome Center Core Services at the University of California, Davis for GC-MS analyses. Briefly, the dried supernatants were derivatized as described by Sana et al. [30]. All samples were spiked with a mixture of fatty acid methyl esters of C8, C9, C10, C12, C14, C16, C18, C20, C22, C24, C26, C28 and C30 linear chain length which served as an internal retention index [30], [31]. An Agilent 6890 gas chromatograph (Santa Clara, CA) containing a 30 m long, 0.25 mm i.d. rtx5Sil-MS column with an additional 10 m integrated guard column was used to run the samples. The Agilent 6890 was controlled by the Leco ChromaTOF software version 2.32 (St. Joseph, MI). Resulting text files were exported to a data server with absolute spectra intensities and further processed by a filtering algorithm implemented in the metabolomics BinBase database [32]. Quantification was reported as peak height using the unique ion as default. Metabolites were unambiguously assigned by the BinBase identifier numbers using retention index and mass spectrum as the two most important identification criteria. Additional confidence criteria were used by giving mass spectral metadata, using the combination of unique ions, apex ions, peak purity and signal/noise ratios. All database entries in BinBase were matched against the Fiehn mass spectral library (http://fiehnlab.ucdavis.edu/Metabolite-Library). Data normalization was performed as described in Fiehn et al. [31], using total metabolite content. The resulting data underwent a log transformation and was subjected to multivariate analyses and significant feature identification using MetaboAnalyst, a web-based metabolomics data processing tool (http://www.metaboanalyst.ca) [33].

### Gene expression analyses from plant root tissue

Total RNA was isolated from frozen root tissues (see above) using TriReagent (Sigma, MO), and was quantified with a Nanodrop ND-1000 Spectrophotometer (Thermo, DE). RNA integrity was checked on a formamide denaturing agarose gel. Two µg of purified total RNA were reverse-transcribed using Superscript III RT and a poly (T) primer (Invitrogen, CA) at 42°C for one hour according to the manufacturer's instructions. The reaction product was diluted to a concentration of 50 ng µl^−1^ and 1 µl was used for each PCR reaction. The PCR reaction mix (20 µl) contained 0.4 µmoles of each gene-specific primer, 200 µmoles of dNTPs, 1× reaction buffer and one unit of Taq DNA polymerase (Takara, Japan). PCR included 30 cycles of 94°C for 30 s, 56°C for 30 s and 72°C for 2 min in a GeneAmp 2700 thermal cycler (Applied Biosystems, CA). Actin primers were used as a control to determine the uniformity of the concentration of cDNA. The gene-specific primers used for RT-PCR assays are listed in Table S1.

### Soil Experiment

Soil with a history of exposure to *Arabidopsis* was collected in July 2011 from the Michigan Extension Station, Benton Harbor, MI (N42°05′34″, W86°21′19″ W, elevation 630 feet). The top 5–10 cm of soil was collected from under three patches of *Arabidopsis thaliana* that have been growing naturally in a fallow field for more than 8 years. All the necessary permits were obtained for the described soil. Broeckling et al. [13] described the soil in detail although from a different collection event. Soil from the same site although collected at other time points was used in previous experiments by Badri et al. [11] and Broeckling et al. [13]. The soil was transported to the laboratory in air tight coolers and stored in a cold room (4°C) until further use. At the time of use, the soil was dried at room temperature, homogenized by hand, and cleaned of plant debris. Pots (2×6×6-cm) were lined with Whatmann 3 MM filter paper to avoid soil loss. The pots were placed in a growth chamber at 25°C with a photoperiod of 16 h light/8 h dark. Six replicate pots were maintained for each of the four developmental time points. Pots without plants served as a bulk soil control that could be contrasted with rhizosphere communities under the influence of the host plant. *Arabidopsis* seeds were surface-sterilized and grown on MS plates as described above (see plant material and growth conditions). One seven day old seedling was transplanted to each of the six pots. Plants were grown until they were: 17 days, 24 days, 31 days or 38 days old, bulk soil was collected along with the 38 day old sample (Figure S1).

### Extraction of microbial RNA from soil

For each of the 5 time points (17, 24, 31, 38 days and bulk soil) 6 replicate pots were maintained, rhizosphere soil was collected by obtaining the soil attached to the roots of the plant and bulk soil was collected from the center of the pot. Soil samples were transferred to 2.0 ml tubes, immediately frozen in liquid nitrogen and stored at −80°C until processing. A total of 6 rhizosphere soil samples were collected for each time point (17, 24, 31, 38 days and bulk soil; 30 samples total). Total RNA was extracted from each soil sample using the PowerSoil® total RNA isolation kit (MoBio, CA), with slight modifications to the manufacturer's instructions. The modifications were as follows: after solution SR2 was added to the bead tube, the solution was vortexed at maximum speed for 30 minutes instead of 5 minutes. After the phenol: chloroform: isoamyl alcohol was added to the bead tube, the bead tube was shaken at 200 rpm for 30 minutes instead of being vortexed at high speed for 10 minutes. RNA integrity was checked on a formamide denaturing agarose gel. Microbial RNA was quantified using a Nanodrop ND-1000 spectrophotometer. All RNA samples that had an A_260_∶A_280_ ratio between 1.7 and 2.0 were processed for metatranscriptomics.

### Pyrosequencing and analyses

Total RNA collected from the 6 pots per time point were pooled and 15 µg of total RNA from each time point and bulk soil were sent to the W.M. Keck Center for Comparative and Functional Genomics, Roy J. Carver Biotechnology Center, University of Illinois at Urbana-Champaign, where steps from mRNA isolation to pyrosequencing were performed. Briefly, for each of the time points which consisted of 6 pooled replicates ribosomal RNA was removed from 5 µg of total RNA using the Ribozero rRNA removal Meta-bacteria kit (Epicentre Biotechnologies, WI). The mRNA was converted to cDNA by using barcoded random hexamer primers and nebulized with the nebulization kit supplied with the GS Titanium library preparation kit (454 Life Sciences, CT). Each sample (17, 24, 31, 38 days and bulk soil) was given a unique 10 bp sequence barcode and the cDNA libraries of each sample were normalized by using the Trimmer Direct kit (Evrogen, Russia) following the manufacturer's instructions and as previously described [34]. cDNA normalization equalizes the number of gene copies in the library which allows for the discovery of new genes that are transcribed at low levels [35]. The normalized barcoded cDNA libraries were pooled in equimolar concentrations based on average fragment length and concentration. The pooled libraries were quantified using a Qubit fluorometer (Invitrogen, CA) and average fragment sizes were determined by analyzing 1 µl of each sample on a Bioanalyzer (Agilent, CA) using a DNA 7500 chip. The pooled library was diluted to 1×10^6^ molecules µl^−1^. Emulsion-based clonal amplification and sequencing on the 454 Genome Sequencer FLX+ system was performed according to the manufacturer's instructions (454 Life Sciences, CT). 454 pyrosequencing was performed on 1/8 of a PicoTiter-Plate (454 Life Sciences, CT). Signal processing and base calling were performed using the bundled 454 Data Analysis Software v2.6. 454 sequencing yielded a total of approximately 166,250 sequence reads. MG-RAST [36] and Mothur [37] were used for quality screening and sequence processing for each of the 5 samples. Sequences were screened on the following criteria: sequences derived from *A. thaliana* were removed using the Bowtie algorithm [38]. Sequences were dereplicated and filtered by length to remove sequences that differed by more than two standard deviations from the mean length. Sequences were dropped if they contained five or more ambiguous bases, or appeared to be ribosomal RNA. To equalize sampling effort across time points, a random subset of 14,740 high-quality sequence reads were selected for each time point. A summary of the 454 pyrosequencing data for each sample is found in Table S2. Sequence reads were assigned to the Kyoto Encyclopedia of Genes and Genomes (KEGG) [39] subsystem categories using the MG-RAST web-server pipeline. A minimum percent identity cutoff of 70% between our sequences and the sBLAT database and an E-value cutoff of 10^−5^ was used for further quality control.

### Correlating rhizosphere microbial function with host plant root exudation

Correlation analyses (SAS ver. 9.3; SAS Institute, NC) were performed with the transformed data of the 17, 24, and 31 day metabolomics data with that of the corresponding metatranscriptomics data as follows: the average of the transformed GC-MS identified compounds were correlated with the overall functional genes identified by Level 2 KEGG orthology as being involved in Carbohydrate Metabolism, Metabolism of Amino Acids, and Metabolism of Secondary Metabolites (which includes the KEGG level 2 categories of Biosynthesis of Other Secondary Metabolites and Metabolism of Terpenoids and Polyketides) with the compounds categorized as sugars, amino acids and phenolics. We further performed a more in depth correlation of the individual compounds from our metabolomics data through development with the corresponding functional genes at the KEGG functional level. The interactive pathways explorer (iPath2) [40] was used to map the functional genes involved in Metabolism, specifically Carbohydrate Metabolism, Amino Acid Metabolism, Biosynthesis of Other Secondary Metabolites and Metabolism of Terpenoids and Polyketides along with the root exudate compounds categorized as amino acids, phenolics and sugars (Figure S2)

## Results

### *Arabidopsis* root exudation over a developmental time course

**Figure 1:**
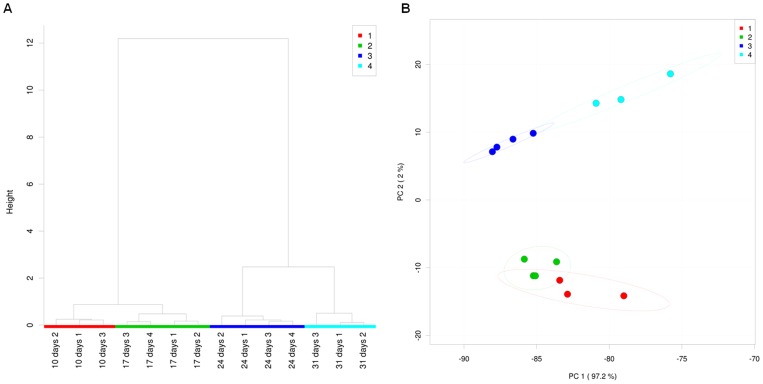
Multivariate analysis of the root exudates analyzed by GC-MS. (A) Cluster analysis (Ward method) based on the 107 compounds detected in root exudates collected at 7–10d, 14–17d, 21–24d, and 28–31d. (B) Principal Component Analysis (PCA) of the 107 root exudate compounds from samples collected at 7–10d, 14–17d, 21–24d, and 28–31d. Dashed ellipses indicate the 95% confidence region of each time point. Red: 7–10 days; Green: 14–17 days; Blue: 21–24 days; Aqua: 28–31 days. 1: 7–10d; 2: 14–17d; 3: 21–24d; 4: 28–31d.

The primary and secondary metabolites present in the root exudation profiles of *in vitro* grown wildtype Col-0 *Arabidopsis* through a developmental time series were analyzed by GC-MS. After normalization, 107 compounds were detected. Among these, 57 compounds were identified (Table S3) based on the mass spectral library database developed by the Fiehn laboratory (University of California, Davis), which includes sugars, sugar alcohols, amino acids, organic acids, fatty acids, phenolics, etc. Hierarchical analysis using a Ward clustering algorithm and Pearson's correlation as a similarity measure revealed that the root exudate profile at each time point clustered separately and that the early (7–10 days and 14–17 days) and later (21–24 days and 28–31 days) developmental time points formed two distinct groups (Figure 1A). Principle component analysis (PCA) also showed that the root exudate profiles of early and later developmental time points clustered separately from each other (Figure 1B). Most of the variability in the data could be accounted for by component 1 (97.2%), while component 2 accounted for 2% of the variability in the data. The identified compounds contributing most to component 1 in the PCA were glycerol, ethanolamine, fructose, glucose, glycine, alanine and tagatose. The identified compounds contributing most to component 2 were oxoproline, γ-Aminobutyric acid (GABA), urea, isoleucine, galactose and tagatose. These data clearly indicate that the quantitative composition of *Arabidopsis* root exudates varies at each developmental stage.

**Figure 2:**
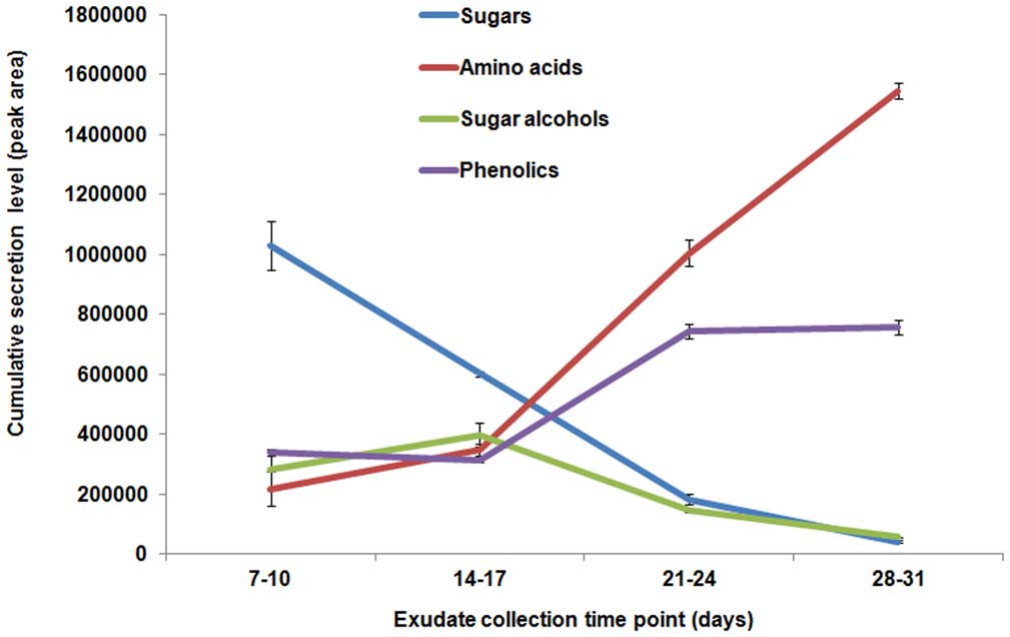
*Arabidopsis* root exudate composition across development. Identified compounds were grouped into chemical classes, and secretion levels were calculated based on cumulative peak area after normalization.

We broadly categorized the 57 identified compounds into four groups: sugars, sugar alcohols, phenolics and amino acids. In total, we identified nine sugars, six sugar alcohols, twelve amino acids and twenty-seven phenolic compounds (Table S3). The compounds categorized as phenolics consisted of compounds belonging to organic acids, carboxylic acids, fatty acids and phenolics. For each group of compounds, cumulative secretion levels where calculated in order to identify potential patterns found throughout development. These cumulative secretion levels did indeed follow a trend depending upon the developmental stage of the plant. For instance, the secretion levels of sugars and sugar alcohols were higher at early developmental time points and gradually lowered at later developmental time points of the plant (Figure 2). On the contrary, the cumulative secretion levels of amino acids and phenolics were low during the early developmental time points, but rose at later developmental time points of the plant (Figure 2).

### Root gene expression analyses

**Figure 3:**
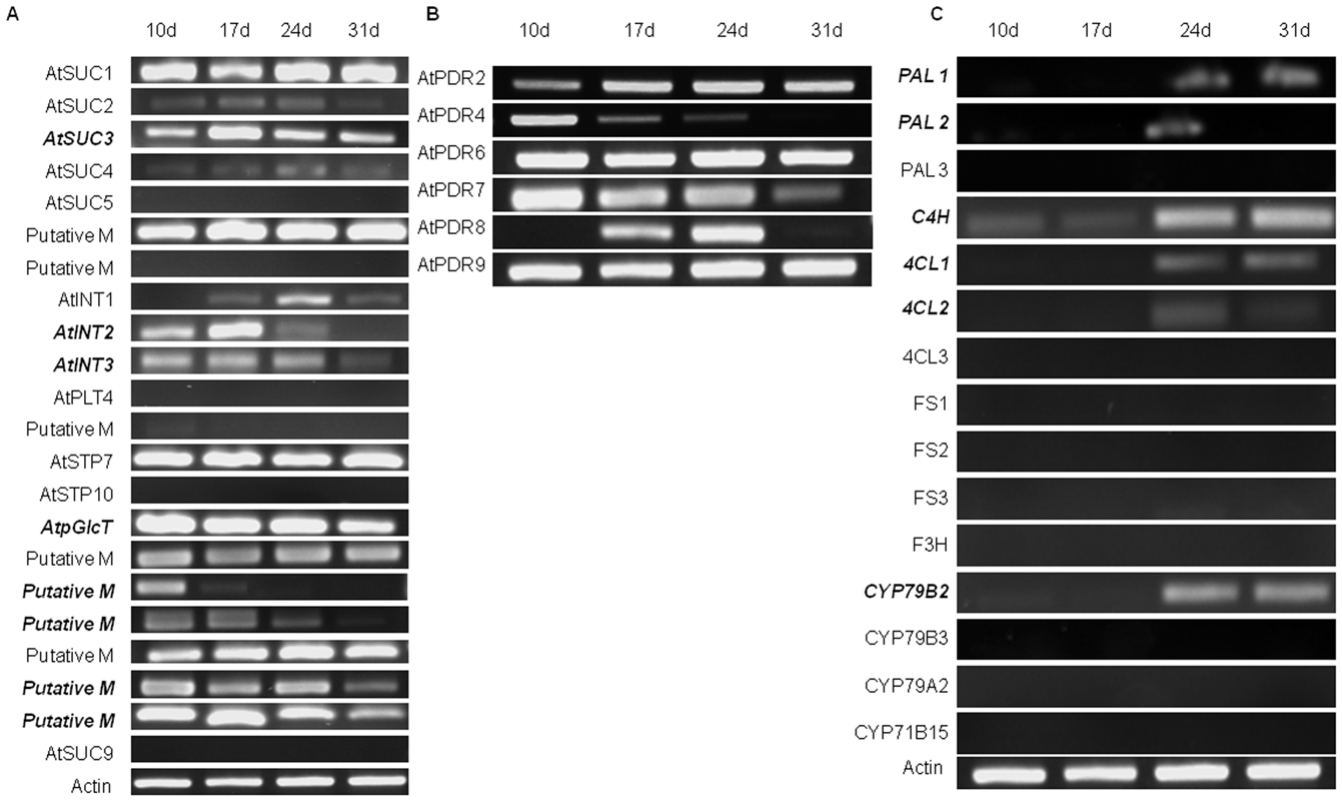
*Arabidopsis* root gene expression profiles. (A) sugar transporters, (B) ABC transporters, and (C) genes involved in secondary metabolism, as measured by semi-quantitative RT-PCR. Gene names are listed on the left side. The time points at which root tissues were collected are shown along the top. Bold and italicized text indicates genes whose expression profiles are most consistent with the GC-MS analysis of collected exudates.

To validate the observed trends in root exudation over developmental time, we examined the gene expression of sugar transporters, ABC transporters and genes involved in secondary metabolite biosynthesis in the root tissues of 10, 17, 24 and 31 day old plants by semi-quantitative RT-PCR. In total, gene expression patterns were analyzed for 43 genes, including twenty-two sugar transporters (Figure 3A), six ABC transporters (Figure 3B) and fifteen secondary metabolite biosynthesis genes (Figure 3C). The expression of 22 sugar transporter genes varied with development. Among those, the expression levels of eight sugar transporters (AtSUC3, AtINT2, AtINT3, AtpGlcT, and four genes belonging to the putative monosaccharide transporter family) were higher in early plant development and gradually decreased at later developmental stages (Figure 3A). This result is in agreement with our GC-MS data showing more sugars secreted at early stages of plant development. The expression of six sugar transporters (AtSUC5, AtPLT4, AtSTP10, AtSUC9 and two genes belonging to the putative monosaccharide transporter family) was not detectable in our RT-PCR analyses and the expression of five sugar transporters (AtSUC1, AtSTP7 and three genes belonging to the putative monosaccharide transporter family) was constant throughout the time points. The remaining three sugar transporters (AtSUC2, AtSUC4 and AtINT1) showed an increase in gene expression until 24 days and then decreased at 31 days.

Among the six ABC transporters (AtPDR2, AtPDR4, AtPDR6, AtPDR7, AtPDR8 and AtPDR9) that were analyzed, none showed a definite pattern of gene expression with respect to the developmental stages of the plant. For the most part the ABC transporter genes showed consistent expression over time (AtPDR2, AtPDR6, ATPDR7 and AtPDR9), with the exception of AtPDR4 which showed high gene expression at 10 days but expression decreasing to undetectable levels by 31 days. AtPDR8 was equally expressed at 17 days and 24 days, but was not detected at the other time points (Figure 3B).

We also analyzed the expression of fifteen genes involved in secondary metabolite biosynthesis such as the phenylpropanoid pathway (Figure 3C). Several of these genes were only expressed or were more highly expressed at later stages of plant development. These included PAL1 and PAL2 (phenylalanine ammonia-lyase), C4H (cinnamate-4-hydroxylase), 4CL1 and 4CL2 (4-coumarate-CoA ligase), and CYP79B2 (involved in converting tryptophan to indole-3-acetaldoxomine, a precursor of indole glucosinolates and indole-3-acetic acid) (Figure 3C). However, the expression of the remaining nine genes (PAL3, 4CL3, FS1, FS2, FS3, F3H, CYP79B3 and CYP71B15) tested was not detectable in our RT-PCR analysis.

### Correlations between root exudation patterns and the functional capacity of the soil microbiome

Measuring the patterns of root exudation identified in this study on plants growing in natural soils is problematic due to technical sensitivity limitations and confounding factors such as the release and modification of compounds by other organisms in the soil. Therefore, we correlated the *in vitro* root exudation patterns with the functional capacity of the rhizosphere microbiome to metabolize these compounds at different developmental stages of Arabidopsis grown in natural soils.

We performed a metatranscriptomic analysis to correlate rhizosphere microbial functions with root exudation patterns corresponding to different stages of plant development. A total of 14,740 sequences for each time point (Table S2) were uploaded to the Metagenomics-RAST (MG-RAST) server [36] and annotated to the Kyoto Encyclopedia of Genes and Genomes (KEGG) hierarchical classification within MG-RAST to assign a putative function to each sequence (KEGG hierarchical annotations for each sample is provided in Table S4). KEGG divides the functional genes obtained into a four level hierarchy with the first level consisting of five categories: metabolism, genetic information processing, environmental information processing, cellular processes and human diseases (Table S5) [39]. In our data, metabolism accounted for over 50% of the functional genes in each sample (Table S5). Since the metabolic activity of the soil microbes is presumable directly tied to their utilization of the root exudate compounds identified via GC-MS we focused on the metabolic activity of the soil microbes.

We performed Pearson correlation and Spearman rank correlation analyses between the group of exudate components and the abundance and number of related microbial functional genes assigned to specific KEGG metabolic functions at the different time points. Indeed, we observed that the exudation of phenolics by the plant through development was significantly (p<0.05) positively correlated with both the abundance and number of expressed microbial genes involved in secondary metabolism (r = 0.98 and 0.95, respectively; Table 1). Similarly, there was a positive correlation between the amino acids released as root exudates and the number of expressed microbial genes involved in Amino Acid Metabolism at each time point (ρ = 1.00; Table 1). Correlations between overall Carbohydrate Metabolism performed by the soil functional microbiome and the sugars released as root exudates were not statistically significant (Table 1).

**Table 1:** Correlation of the compounds identified in the root exudates with the abundance or number of functional genes in each sample.

	Corresponding functional genes in each sample		Abundance of corresponding functional genes in each sample	
	Pearson	Spearman	Pearson	Spearman
Amino Acids	0.86	1.00*	0.63	0.80
Sugars	−0.55	−0.40	−0.24	0.20
Phenolics	0.95*	0.80	0.98*	0.80

* Correlation was statistically significant (p<0.05).

Correlation analyses were also performed between specific compounds present in the root exudates and the functional genes involved in their metabolism. For example, beta-alanine positively correlated to the functional gene adenylosuccinate synthase [EC:6.3.4.4] which is involved in the alanine, aspartate and glutamate metabolism pathway (Table S5). Likewise, glycine positively correlated with sarcosine oxidase, subunit alpha [EC:1.5.3.1] and sarcosine oxidase, subunit beta [EC:1.5.3.1] functional genes involved in the glycine, serine and threonine metabolism pathway (Table S5). On the other hand, 41 functional genes involved in Amino Acid Metabolism negatively correlated with asparagine. Some functional genes that negatively correlate with asparagines were: shikimate dehydrogenase [EC:1.1.1.25], hydroxypyruvate reductase [EC:1.1.1.81], or anthranilate phosphoribosyltransferase [EC:2.4.2.18](Table S5). Similarly, the functional gene farnesyl diphosphate synthase [EC:2.5.1.1 2.5.1.10] involved in the terpenoid backbone biosynthesis pathway positively correlated with six compounds categorized as phenolics (4-hydroxybutyric acid, capric acid, lauric acid, palmitic acid, propane-1,3-diol, and stearic acid; (Table S6)). We observed that the sugars arabinose, fructose and sucrose positively correlated with 27 functional genes involved in carbohydrate metabolism such as chitin synthase [EC:2.4.1.16]; glucan endo-1,3-beta-D-glucosidase [EC:3.2.1.39]; glucan 1,3-beta-glucosidase [EC:3.2.1.58] or rhamnulose-1-phosphate aldolase [EC:4.1.2.19] (Table S7).

The iPATH 2 KEGG map (Figure S2) visually illustrates that the compounds released as root exudates could be utilized by the soil microbial functional genes. For example, we identified sucrose in the plant root exudates (Table S3). Sucrose can be used by two of the identified microbial functional genes alpha-glucosidase [EC:3.2.1.20] and beta-fructofuranosidase [EC:3.2.1.26] for the production of glucose and fructose. Similarly, galactose is used by the functional gene galactokinase [EC:2.7.1.6] to make alpha-D-galactose-1-phosphate. Beta-alanine is used to make 3-oxoproponoate and L-Glutamate by beta-alanine–pyruvate transaminase [EC:2.6.1.18].

## Discussion

Soil microbial communities are able to utilize and are impacted by root exudates in a variety of ways. For example, *Bacillus subtilis*, *Rhizobium leguminosarum*, or *Agrobacterium tumefaciens* C58C1 are just a few examples of soil bacterial species that utilize and exhibit chemotaxis towards a wide variety of sugars [41]–[43]. Rhizobia use specific flavonoids for initiating symbiosis [6]. *Agrobacterium* is chemotactically attracted to certain phenolics, such as acetosyringone [44], while *Pseudomonas putida* is able to catabolize flavonoids, such as naringen, p-hydroxybenzoic acid and quercetin for use as nutritional sources [45]. Similarly, many gram positive bacteria use amino acids or modified peptides as signal molecules [46].Here we present evidence that root exudation of primary and secondary metabolites by *Arabidopsis* changes with plant development and follow specific trends. For instance, the quantity of amino acids and secondary metabolites (phenolics) released from the roots increased over developmental time. On the other hand, sugars were released in the greatest abundance early in the plant's life cycle. These patterns were corroborated by root gene expression analyses, which showed higher expression of the majority of sugar transporter genes tested in the early stages of plant development. Similarly, higher expressions of the genes involved in the phenylpropanoid pathway were seen in later stages of plant development. Sugars serve as ready sources of energy for microbial growth [47], and secondary metabolites, such as those categorized as phenolics in this study, may function as antimicrobials and signaling molecules in the rhizosphere [6], [8], [48], [49].

Plant secondary metabolites' are known defense signals [50] that play important roles in disease resistance [51], [52], in adapting to the changing environment and overcoming stress [53]. Their increased release at later stages in the plants life cycle is in agreement with De-la-Pena et al. [1], where defense related proteins showed enhanced secretion during flowering time. The observed increase in the exudation of phenolics at later developmental stages was mirrored by a corresponding increase in microbial functions related to the metabolism of secondary metabolites (Table 1). Microbes can quickly evolve a variety of mechanisms to detoxify and overcome the effects of potentially harmful chemicals either by chemically modifying the toxin, metabolizing the toxin or by extruding the toxin from their cells. For instance, studies analyzing the effect of the toxic compound toluene on the soil bacterial metaproteome have shown an increase in ABC transporter activity after toluene amendment, presumably due to export of the toxic substance out of the bacterial cell [54], [55]. Likewise, we observed that as the host plant aged, there was an increase in the expression of ABC transporters and genes involved in membrane transport among soil microbes peaking at 24 days when phenolics are at their most abundant secretion time point (data not shown).

Rates of sugars' root exudation decreased with plant development and this trend did not correlate with the overall functional genes categorized under Carbohydrate Metabolism. This can be due to the fact that many pathways and cycles utilizing sugars such as glycolysis and the citric acid cycle are synergistically regulated by both sugars and amino acids [56]. Although the correlation of the overall group of compounds categorized as sugars present in the root exudates did not correspond with the whole Carbohydrate Metabolism functional genes, we did see individual sugar compounds positively correlating with particular functional genes involved in Carbohydrate Metabolism (Table S7). For example sucrose, arabinose and fructose positively correlated with 27 functional genes involved in carbohydrate metabolism (Table S7). These results are indicative of the microbial community actively utilizing these specific compounds released by the plant. Studies have shown that *Sinorhizobium meliloti* carries genes necessary for the catabolism of arabinose [57]. Fructose and alanine have also been shown to produce a positive metabolic priming effect on soil microbes that is manifested in the increased degradation and mineralization of more complex soil organic matter when compared to simple sugars [58]. The priming effect is thought to be due to the ability of these easily available substrates, i.e. fructose and alanine, to activate microbial metabolism and increase enzyme production [59]. The enhanced enzyme production presumably increases the metabolic capability of the soil microbiome, which in turn benefits the plant as various limiting nutrients can be made available.

Rates of amino acid exudation increased with plant development, and this trend was mirrored by an increase in the number and abundance of expressed functional genes related to the metabolism of Amino Acids (Table 1). We observed significant correlations between specific amino acids and functional genes involved in Amino Acid Metabolism (Table S5). Amino acid availability is necessary for bacterial root colonization. For example, *Pseudomonas fluorescens Pf0-1* shows chemotactic response towards tomato roots due to L-amino acids found in its root exudates [60]. Similarly, *Pseudomonas fluorescens* strain WCS365 colonizes the tomato root in the presence of amino acids such as: aspartic acid, glutamic acid, isoleucine, leucine and lysine [61]. Our studies identified the release of the amino acid isoleucine (Table S3), which has been shown to be one of the major amino acid components required for the colonization of *Pseudomonas fluorescens* on tomato roots [61]. Interestingly, amino acid exudation of rice increased upon the plants exposure to *Cyanobacterium* sp. (Sb26) and *Rhizobium* sp. (Sb16) [62] presumably due to certain microbial products that are able to trigger amino acid exudation [63]. Because we collected exudates in an axenic system, this suggests that the amino acid concentration released by the plant in a more natural setting (i.e. when the plant is interacting with its biotic environment, in this case a soil microbial community) may be even higher than we observed here.

Ample evidence demonstrates that plant root exudates mediate the selection of specific rhizosphere microbes. However, there is no information available on how plants and their root exudates influence the rhizosphere microbiome functioning over the course of plant development. In this study, we suggest that plant root exudates have associations with rhizosphere microbial functions, and that these interactions are dependent upon the plant developmental stage. These observed trends of *in vitro* collected root exudates and their correlation with rhizosphere microbial functions might hint that the qualitative changes in root exudation observed through plant development are genetically regulated and independent of the microbial community. On the other hand, quantitative changes in root exudates might be attributed to the microbial community. In other words, the soil microbial community is able to modify plant root exudation but not control it. For example the increased exudation of secondary metabolites and amino acids through plant development might be indicative of innate defensive priming by the plant. Studies analyzing the metabolite profiles of potatoes after pathogen inoculation showed that 42 metabolites (consisting of sugars, amino acids, organic acids and fatty acids) significantly increased or decreased [64]. Similar to our exudation profiling over a developmental time course, metabolite profiling by Abu-Nada et al. [64] revealed that pathogen inoculation lead to an up-regulation of amino acids and a down-regulation of sugars. Although our exudation profiles were obtained axenically, we observed the release of compounds that are released as defensive and priming strategies against pathogens or as attractors for beneficial microbes. This may mean that as plants develop and start to set seed, they begin to adopt a more defensive strategy. Incidentally, it was previously reported that defense related proteins are secreted by roots in higher concentrations at flowering time [1].

Root exuded metabolites can also have a dual role with respect to their effect on microbes. For example, GABA that in our studies increased following a developmental pattern has been shown to reduce *Agrobacterium tumefaciens* virulence by quenching quorum-sensing [65]; while the beneficial bacteria *Pseudomonas putida* is able to use GABA as its sole nutrient source [66]. In contrast, proline nullifies GABAs ability to quench quorum-sensing [67]. While proline catabolism by the symbiont *Rhizobium meliloti* aids in its ability to colonize the root and establish symbiosis [68]. In our study, GABA and oxoproline (which is very similar in structure to proline) increase following plant development (Table S3). Experimentally these two signals have been shown to work in opposing fashion with respect to plant *Agrobacterium* infection [65], [67] yet understanding the interplay of these signals in a complex environment like the rhizosphere still needs to be explored.

In connection to these patterns in root exudation, our rhizosphere metatranscriptomics data showed that fewer functional genes were uniquely expressed at early plant developmental time points compared with later developmental stages (Table S2). Thus, it is possible that: 1) high sugar levels exuded in early plant developmental stages may attract a wide range of microbes expressing a limited number of genes (which are similar across taxa) involved in the utilization of sugars as general substrates, and 2) high levels of phenolics exuded in later plant developmental stages might induce the expression of genes belonging to more specialized pathways, where these compounds are used as specific substrates or signaling molecules in ways that vary across taxa. This hypothesis implies that the plant attracts a wide range of microbes in the early stages of development when compared to later developmental stages by secreting sugars which are readily available for metabolism. As the plant develops, it begins to select among rhizosphere inhabitants by releasing phenolics and amino acids. The increase in the number of uniquely expressed microbial functional genes at later plant developmental stages may be indicative of a community-wide microbial response to shifting exudation toward more recalcitrant or inhibitory compounds. Thus, only those microbes that have evolved means of detoxifying or utilizing these compounds will thrive. A detailed analysis of the rhizosphere microbes associated with Arabidopsis plants at different stages of development may provide a means of answering some of these questions. Micallef et al. [12] used denaturing gel gradient electrophoresis to demonstrate that rhizospheric microbial communities change with plant development. Further studies identifying the taxonomic microbial community of the rhizosphere would allow us to identify how specific microbial taxa are influenced by root exudation.

In nature, root exudation is affected by a myriad of factors. Here we analyzed the compounds released as root exudates in a controlled environment and correlated them with the functions present in the rhizosphere. Although we did see correlations between the identified compounds in the *in vitro* studies and the functions carried out in the rhizosphere *in vivo*, additional work is needed to clarify the impacts of root exudation changing not only with plant development, but also in response to the specific microbiomes present at each developmental stage. It is important to note that in our rhizosphere soil community analysis we have not excluded the contributions of components such as proteins and polysaccharides that contribute to root exudation. Our exudate profiling was not exhaustive, and other root exudate constituents, such as proteins and polysaccharides, might also contribute to the changes observed in the functional microbiome at different stages of plant development.

Rhizosphere driven selection of microbial functions has the potential to improve the development and health of plants in a sustainable manner. A deeper understanding of soil microbial functions over plant development can help devise better strategies for disease resistance and thus improving plant and soil health. However, further mechanistic studies are required to identify specific microbial candidates that perform certain microbial functions of benefit to the plant, and to uncover pathways for inter-species signaling in the rhizosphere.

## Supporting Information

Table S1 List of the primers used in this study.
(PDF)

Table S2 Summary of the 454 pyrosequencing results for each sample.
(PDF)

Table S3 Table detailing the compounds released via root exudation by the plant as it develops.
(PDF)

Table S4 Hierarchical classification of the functional genes in each sample they are categorized under KEGG orthology.
(PDF)

Table S5 Pearson correlation analysis of the functional genes involved in Amino Acid Metabolism with root exudate compounds classified as amino acids. Values present significantly correlate at p<0.05.
(XLSX)

Table S6 Pearson correlation analysis of the functional genes involved in Metabolism of Secondary Metabolites which includes the Biosynthesis of Other Secondary Metabolites and Metabolism of Terpenoids and Polyketides with root exudate compounds classified as phenolics. Values present significantly correlate at p<0.05.
(XLSX)

Table S7 Pearson correlation analysis of the functional genes involved Carbohydrate Metabolism with root exudate compounds classified as sugars. Values present significantly correlate at p<0.05.
(XLSX)

Figure S1 Soil grown *Arabidopsis thaliana* Col-0 at each plant developmental stage (17, 24, 31 and 38 days).
(TIF)

Figure S2 iPATH 2 KEGG Map exhibiting the functional genes involved in Metabolism with the identified root exudate compounds. Blue lines: functional genes involved in Carbohydrate Metabolism; Green lines: functional genes involved in Amino Acid Metabolism; Brown lines: functional genes involved in the Metabolism of Secondary Metabolites which includes the Biosynthesis of Other Secondary Metabolites and Metabolism of Terpenoids and Polyketides; Red dots: root exudate compounds classified as sugars; Yellow dots: root exudate compounds classified as amino acids; Orange dots: root exudate compounds classified as phenolics.
(PNG)
