# Correction: Root Exudation of Phytochemicals in Arabidopsis Follows Specific Patterns That Are Developmentally Programmed and Correlate with Soil Microbial Functions

**DOI:** 10.1371/annotation/51142aed-2d94-4195-8a8a-9cb24b3c733b

**Published:** 2013-08-28

**Authors:** Jacqueline M. Chaparro, Dayakar V. Badri, Matthew G. Bakker, Akifumi Sugiyama, Daniel K. Manter, Jorge M. Vivanco

Due to a copy and paste error, the expression profile of AtPDR9 in Figure 3B and the Actin panel in Figure 3C were duplicated. We provide the corrected Figure 3 along with the raw data for the corrected bands of AtPDR9 in Figure 3B and Actin in Figure 3C as reference for the readers. Further, we verified the gene expression data for AtPDR9 and Actin by repeating the RT-PCR using the same cDNA used to generate the previous RT-PCR data.

**Figure:**
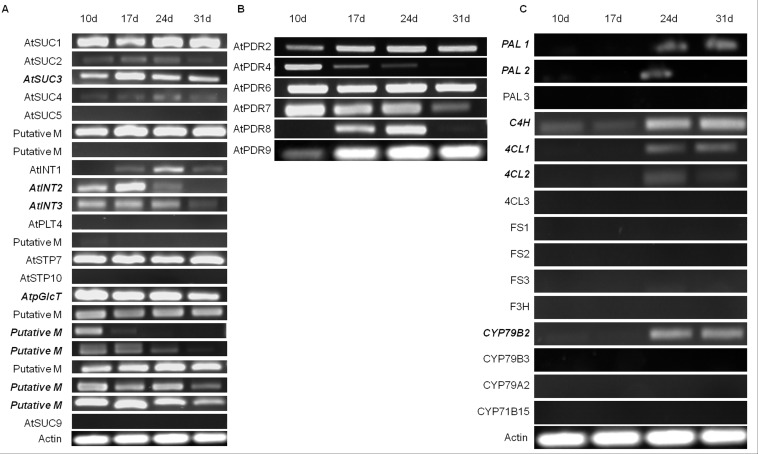


There were errors in the Supporting Information file Table S3. The corrected version of the file are available here: 
