# Supplementary material for: Root Exudation of Phytochemicals in Arabidopsis Follows Specific Patterns That Are Developmentally Programmed and Correlate with Soil Microbial Functions
Source: PLoS One. 2013 Feb 1;8(2):e55731. doi: 10.1371/journal.pone.0055731 (PMC3562227; doi:10.1371/journal.pone.0055731)
Supplement: Table S1 — List of the primers used in this study. (PDF) [file pone.0055731.s001.pdf]

Table S1. List of the primers used in this study. RT-PCR was used to analyze the expression of sugar transporters, ABC transporters and genes involved in secondary metabolism. Putative M refers to genes involved in the putative monosaccharide transporter family. All primers are given in the 5' to 3'orientation.

	AGI Code	Forward	Reverse
AtSUC1	At1g71880	GGAGTCCAATCTGGTGCAAT	GAATCCTCCCATGGTCGTTG
AtSUC2	At1g22710	AACTTCATCCTCGCCATTTG	GCTTTGAAGGCAGGAGCATC
AtSUC3	At2g02860	TGGGGATCCAACAGGAGATA	CCGGTGGACTTGAAAGAACTC
AtSUC4	At1g09960	TATGGGTGCACTTGGTTTGA	GAGAGGGATGGGCTTCTGAAT
AtSUC5	At1g71890	AATCGATTGGTCGGAAAATG	ATAGCCCCTGACATGGCTGG
Putative M	At1g08920	GTGCGTTGCAAGTTGTGACT	CACCGGTAGAGGCCAATAGA
Putative M	At4g04760	AATCGGATGGTTCGCTATTG	CGGTCATCGATGTCTTGTTG
AtINT1	At2g43330	TTGGTCGGTTTAGGAGTTGG	GGCAGCAACAATGAGAGACA
AtINT2	At1g30220	GGGCATGTTGGATCTCTGAT	CATCGATCTTCGTTCAAGCA
AtINT3	At2g35740	TGGTGATGGTGATTGCTCAT	TATCCTCGTCAGCCGTCTCT
AtPLT4	At2g20780	CGTGAGCTTCTTAGCCCATC	GCACGTGACACAGAGAGGAA
Putative M	At1g79820	CCAAATTGTCGGAGTTGCTT	ATAACCCAGTGAACGGCAAG
AtSTP7	At4g02050	TGAATGCTGGAGCTGTGAAC	TGGCATGCAAATAGCCATAA
AtSTP10	At3g19940	CGAGAGAGGCAAAAATGAGG	TGTCCCGCTGGTCTAATTTC
AtpGlcT	At5g16150	CTCTGGCCAAGGTTCTTCTG	CTCTGATTCGGGATGCAAAT
Putative M	At2g48020	GGGGCTCTAACCACACTGAA	TTCTTCCGGCTCTGTCAACT
Putative M	At3g20460	GTTGAAATCGCTCCCAAAAA	ACGCTTGTCACAGACTGCAC
Putative M	At1g67300	CTGCTGCACTACTCGCTCTG	CGCCGAAAAATGTGGTAAGT
Putative M	At1g05030	GAATCACGAGTGGTGCTCAA	GAGCCAAAGCTGGCATAGAC
Putative M	At1g19450	GAGATCGCTCCACAGACCAT	GAGTAGAAGCCGACGACCTG
Putative M	At5g17010	CCCAACCTATTCCGTTCTGA	GCTTTCAAGCACTTCCCTTG
AtSUC9	At5g06170	AGCCGTTGGTTTCTTCGTTG	TCTTACTAATCACTCCAATAACAAGG
AtPDR2	At4g15230	TGGCAAGAGATGAAGTGTCAGGGAAAG	CTACAGCAGGATCTGGAATGATTTCTTGG
AtPDR4	At2g26910	CACGATTCATCAGCCTAGCA	ACATTGTGGTTTGGGGTGAT
AtPDR6	At2g36380	AGATGTTGACGTCACGAATCTTGCT	GTTGCCCTGCGTGAAAAGAATTG
AtPDR7	At1g15210	GGACATACACGCTTCCCACT	AAGCACACTTGTTCCCAACC
AtPDR8	At1g59870	AGAGCAGCGGCTATTGTGATGA	TGGCGTAGACGATGAGTGAGAT
AtPDR9	At3g53480	TGAGGAGAGGTATAACAGGAGGTC	GAGAGATTCAAAGAACGAGAGAGG
PAL1	At2g37040	GATTCTCGAAGCAATTACCAGTTT	GAGGAGAAGTACGAAGAGCGTAAC
PAL2	At3g53260	GTCAGAGTCAACACTCTTCTCCAA	TTACTTCAATTTGAGGACCTAGCC
PAL3	At5g04230	TACAACAACGGGTTACCCTCTAAT	GTTTTCCTTGAGATATAGCCCTGA
C4H	At2g30490	TCCTATCCTTAGACCATTCCTCAG	CTCAGACGAAGAGTCTCCTTAACC
4CL1	At1g51680	AGCTCGATAAGAGTGGTGAAATCT	CATCTTCTGATAACTCCGAATCCT
4CL2	At3g21240	TCGTTAGGGTTTGCTAAAGAGC	AAACACAACCTGTTTTGACACG
4CL3	At1g65060	CTGATCACTACCGATGAACCAA	CACCGGAAAGAACGAATCTAAC
FS1	At1g49390	GCCATCGATCTCAGTCTTCTCT	TGGACATGGAGGAAAGAAGTT
FS2	At5g63580	GGACCGAGAATCTTTTTCACAG	AGAGGAGGGAATGTAGTGGACA
FS3	At5g63590	CTGATACTGCGGTTGCTACAAG	CGTCGATCCATTGGTTATCTTT
F3H	At5g07990	TCCCTTAAAGGAACTGATCTTGAC	GAATCTCTCGGGTTTAAATGCTAA
CYP79B2	At4g39950	CAGAAGATCCTCTCTAACGGCTAC	CGATTTGAGTTCTCTTTCCTTCTC
CYP79B3	At2g22330	CTACACGACAATAGAGCTGAGGAA	ACCGTAACGGCTAAGTAAAACTTG
CYP79A2	At5g05260	GAGATTCTGAAGAAGCAAGACTCC	GATGGGTTAAAACCGTAAACAAAG

	AGI Code	Forward	Reverse
CYP71B15	At3g26830	GAGGAAGTGCTAAAGATCAACGAT	ATTCTTCAATCTCCTGTTCTGACC
β-Actin		CAACTGGGACGAYATGGAGA	GAGTCATCTTCTCTCTGTTGGCC
