# Supplementary material for: Root Exudation of Phytochemicals in Arabidopsis Follows Specific Patterns That Are Developmentally Programmed and Correlate with Soil Microbial Functions
Source: PLoS One. 2013 Feb 1;8(2):e55731. doi: 10.1371/journal.pone.0055731 (PMC3562227; doi:10.1371/journal.pone.0055731)
Supplement: Table S2 — Summary of the 454 pyrosequencing results for each sample. (PDF) [file pone.0055731.s002.pdf]

Table S2. Summary of the 454 pyrosequencing results for each sample.

	17d	24d	31d	38d	Bulk Soil
Total Number of Sequences uploaded to MG-RAST	14740	14740	14740	14740	14740
Mean Sequence Length	380	425	415	437	412
Predicted proteins with known function	7571	8632	7650	8699	8679
Predicted protein with unknown function	6194	5702	6114	5490	5959
Predicted Protein Features	14707	15904	15442	15829	15908
Identified Protein Features	7795	9093	8219	9245	9013
Identified Functional Categories to the M5NR protein datbase[a]	6858	8231	7165	8265	8154
Predicted KEGG Orthology[b]	4449	4255	4573	4750	4047
Unique Features in KEGG hierarchical classification	1694	1533	1489	1797	1366
Reads attributed to the unique feature of KEGG hierarchical classification[c]	2142	1611	1647	1916	1420
KEGG functions expressed in each sample after hierarchical classification	528	516	553	560	439
Unique KEGG functions for each time point	139	114	145	145	98
Reads attributed to unique KEGG functions for each time point	225	234	256	259	204

[a] M5NR protein database consists of NCBI's nr, KEGG database, EGGnogs, and SEED database

[b] Report abundances using the KEGG protein database that include all functional labels

[c] Report abundances using the KEGG protein database that supports hierarchical relationships between functions
