# Supplementary material for: Root Exudation of Phytochemicals in Arabidopsis Follows Specific Patterns That Are Developmentally Programmed and Correlate with Soil Microbial Functions
Source: PLoS One. 2013 Feb 1;8(2):e55731. doi: 10.1371/journal.pone.0055731 (PMC3562227; doi:10.1371/journal.pone.0055731)
Supplement: Table S3 — Table detailing the compounds released via root exudation by the plant as it develops. (PDF) [file pone.0055731.s003.pdf]

Table S3. Table detailing the compounds released via root exudation by the plant as it develops. These were collected over a period of 3 days (7-10 days, 14-17 days, 21-24 days and 28-31 days). Compounds were detected using GC-MS. Numbers indicate the average area under the curve of three replicates, numbers in parenthesis indicate the standard deviation.

Compound	Category	7-10 days	14-17 days	21- 24 days	28-31 days
1-deoxyerythritol	Sugar Alcohol	1933.06 (260.45)	1933.06 (55.06)	11315.12 (1060.15)	90.38 (90.38)
2-hydroxyvaleric acid	Phenolics	12057.96 (1049.56)	12057.96 (1357.67)	17989.5 (1910.57)	285.01 (285.01)
3-hydroxybutanoic acid	Phenolics	26410.08 (7734.05)	26410.08 (2004.28)	2061.1 (162.69)	5.28 (5.28)
3-hydroxypropionic acid	Phenolics	9308.5 (905.44)	9308.5 (615.95)	22432.5 (476.55)	131.67 (131.67)
4-hydroxybutyric acid	Phenolics	3118.83 (269.45)	3118.83 (115.35)	2332.9 (118.05)	39.05 (39.05)
alanine	Amino Acid	20380.66 (2154.44)	20380.66 (790.75)	125416 (11632.23)	8756.43 (8756.43)
arabinose	Sugar	4701.83 (683.13)	4701.83 (362.88)	3108.75 (312.58)	399.06 (399.06)
arabitol	Sugar Alcohol	1252.54 (224.23)	1252.54 (114.38)	2638.05 (88.27)	48.26 (48.26)
asparagine	Amino Acid	121.44 (26.6)	121.44 (38.16)	420.9 (34.18)	167.65 (167.65)
benzoic acid	Phenolics	4851.96 (815.49)	4851.96 (301.82)	8785.3 (656.46)	708.56 (708.56)
beta-alanine	Amino Acid	414.7 (75.56)	414.7 (44.88)	5441.97 (201.44)	352.57 (352.57)
butyrolactam	Phenolics	9875.53 (1089.79)	9875.53 (615.98)	172080 (16103.72)	6279.88 (6279.88)
capric acid	Phenolics	8792.66 (1241.18)	8792.66 (457.12)	4583.37 (199.49)	195.91 (195.91)
cyclohexylamine	Amino Acid	93297 (34137.08)	93297 (20157.1)	18729.75 (1323.14)	351.85 (351.85)
erythritol	Sugar Alcohol	1000.66 (297.79)	1000.66 (55.92)	4323.15 (573.73)	107.52 (107.52)
ethanolamine	Amino Acid	21175.93 (3919.15)	21175.93 (1869.85)	502022.5 (24338.64)	48001.73 (48001.73)
fructose	Sugar	266676.66 (13868.22)	266676.66 (2743.66)	67314.25 (9310.25)	1344.15 (1344.15)
fucose + rhamnose	Sugar	2614.9 (304.29)	2614.9 (794.75)	28415 (836.67)	331.57 (331.57)
fumaric acid	Phenolics	282.23 (40.36)	282.23 (17.99)	4451.87 (820.7)	699.84 (699.84)
γ-Aminobutyric acid	Phenolics	68.8 (30.13)	68.8 (178.34)	63043.5 (6834.63)	29177.79 (29177.79)
galactose	Sugar	291386.66 (28268.19)	291386.66 (8682.93)	1939.85 (126.17)	46.18 (46.18)
glucose	Sugar	436110 (37741.68)	436110 (4910.03)	42315.75 (6521.47)	695.25 (695.25)
glucose-1-phosphate	Sugar	784.83 (255.06)	784.83 (55.14)	2419.57 (55.19)	78.98 (78.98)
glyceric acid	Phenolics	440.53 (20.64)	440.53 (142.85)	10077.07 (785.22)	637.01 (637.01)
glycerol	Sugar Alcohol	274263.33 (62052.43)	274263.33 (39583.93)	103075.25 (7835.79)	620.74 (620.74)
glycine	Amino Acid	60957.33 (15722.64)	60957.33 (2459.06)	127173 (8954.69)	5529.88 (5529.88)
glycolic acid	Phenolics	6675.4 (1728.38)	6675.4 (258.8)	2019.06 (737.81)	266.5 (266.5)
hydroxylamine	Amino Acid	3858.49 (983.25)	3858.49 (1160.47)	1927.55 (129.2)	970.9 (970.9)
inositol myo-	Sugar Alcohol	2875.86 (619.56)	2875.86 (124.1)	17899.55 (2618.37)	725.12 (725.12)
isoleucine	Amino Acid	342.89 (135.93)	342.89 (210.45)	26118.75 (1584.97)	2707.19 (2707.19)
lactic acid	Phenolics	23300.73 (5238.51)	23300.73 (7104.4)	45983.25 (6544.83)	192.65 (192.65)
lauric acid	Phenolics	12790.33 (460.52)	12790.33 (801.36)	6192.55 (328.03)	146.13 (146.13)
levoglucosan	Phenolics	1869.33 (316.03)	1869.33 (79.29)	3851.27 (89.15)	29.34 (29.34)
N-acetyl-D-hexosamine	Phenolics	169.08 (90.71)	169.08 (29.34)	1896.42 (145.72)	123.55 (123.55)
N-acetyl-D-mannosamine	Phenolics	220.73 (37.44)	220.73 (16.85)	5020 (357.59)	86.99 (86.99)
oxoproline	Amino Acid	80.04 (17.55)	80.04 (34.88)	4896.02 (656.58)	67685.3 (67685.3)
palmitic acid	Phenolics	780.29 (31.94)	780.29 (50.66)	641.72 (24.09)	13.35 (13.35)
pelargonic acid	Phenolics	19089.13 (3845.42)	19089.13 (1136.18)	8041.9 (610.54)	1545.58 (1545.58)
propane-1,3-diol	Phenolics	57358 (4163.92)	57358 (1825.69)	23968.75 (1078.21)	1075.56 (1075.56)
putrescine	Phenolics	9849.96 (1357.04)	9849.96 (2118.03)	3959.92 (156.26)	2999.73 (2999.73)
ribose	Sugar	287.19 (50.08)	287.19 (272.29)	34444.25 (3057.63)	622.33 (622.33)
serine	Amino Acid	7103.1 (1333.6)	7103.1 (1428.11)	99913.75 (3031.47)	3118.76 (3118.76)
shikimic acid	Phenolics	80701 (20108.85)	80701 (2721.09)	5485.27 (927.12)	468.42 (468.42)
stearic acid	Phenolics	30803.33 (4158.47)	30803.33 (1359.91)	5694.55 (282.42)	86.15 (86.15)
succinic acid	Phenolics	193.23 (28.36)	193.23 (46.95)	1215.59 (186.23)	395.21 (395.21)
sucrose	Sugar	28497.63 (6433.51)	28497.63 (1755.13)	1982.77 (153.69)	31.07 (31.07)
tagatose	Sugar	515.14 (24.41)	515.14 (68.69)	54.51 (13.34)	5.96 (5.96)

Compound	Category	7-10 days	14-17 days	21- 24 days	28-31 days
threitol	Sugar Alcohol	217.08 (83.28)	217.08 (130.4)	6245.45 (337.02)	45.25 (45.25)
threonic acid	Phenolics	64.3 (12.65)	64.3 (17.58)	2410.9 (189.88)	188.38 (188.38)
threonine	Amino Acid	1441.9 (361.03)	1441.9 (73.8)	2524.92 (229.55)	914.86 (914.86)
tocopherol alpha	Phenolics	19102 (3951.27)	19102 (704.01)	2505.55 (159.38)	87.43 (87.43)
uracil	Phenolics	666.59 (181.86)	666.59 (139.24)	1422.87 (84.62)	477.83 (477.83)
urea	Phenolics	516.65 (101.66)	516.65 (1675.14)	316592.5 (24815.98)	17260.82 (17260.82)
valine	Amino Acid	8361.83 (1076.37)	8361.83 (575.52)	90746.75 (3294.57)	3983.52 (3983.52)
xylitol	Sugar Alcohol	99.05 (39.74)	99.05 (23.84)	3719.12 (329.48)	78.4 (78.4)
