# Supplementary material for: Root Exudation of Phytochemicals in Arabidopsis Follows Specific Patterns That Are Developmentally Programmed and Correlate with Soil Microbial Functions
Source: PLoS One. 2013 Feb 1;8(2):e55731. doi: 10.1371/journal.pone.0055731 (PMC3562227; doi:10.1371/journal.pone.0055731)
Supplement: Table S4 — Hierarchical classification of the functional genes in each sample they are categorized under KEGG orthology. (PDF) [file pone.0055731.s004.pdf]

Table S4. Hierarchical classification of the functional genes in each sample they are categorized under KEGG orthology. There are 1338 functional genes present throughout the samples (day17, day24, day31, day38 and bulk soil). Sample Day17 has 528 functional genes that account for 2142 reads, sample Day24 has 516 functional genes that account for 1611 reads, sample Day31 has 553 functional genes that account for 1647 reads, sample Day38 has 560 functional genes that account for 1916 reads and bulk soil has 439 functional genes that account for 1420 reads.

Level 1	Level 2	Level 3	Function	KEGG ID	Sequence Reads				
					Day17	Day24	Day31	Day38	Bulk Soil
Cellular Processes	Cell Growth and Death	04112 Cell cycle - Caulobacter [PATH:ko04112]	aspartyl protease family protein	K06985	0	1	0	0	0
Cellular Processes	Cell Growth and Death	04112 Cell cycle - Caulobacter [PATH:ko04112]	ATP-dependent Clp protease ATP-binding subunit ClpX	K03544	14	6	7	1	5
Cellular Processes	Cell Growth and Death	04112 Cell cycle - Caulobacter [PATH:ko04112]	ATP-dependent Clp protease, protease subunit [EC:3.4.21.92]	K01358	12	1	6	6	5
Cellular Processes	Cell Growth and Death	04112 Cell cycle - Caulobacter [PATH:ko04112]	ATP-dependent Lon protease [EC:3.4.21.53]	K01338	23	22	17	14	14
Cellular Processes	Cell Growth and Death	04112 Cell cycle - Caulobacter [PATH:ko04112]	cell division protein FtsA	K03590	0	0	2	0	0
Cellular Processes	Cell Growth and Death	04112 Cell cycle - Caulobacter [PATH:ko04112]	cell division protein FtsQ	K03589	1	0	0	0	0
Cellular Processes	Cell Growth and Death	04112 Cell cycle - Caulobacter [PATH:ko04112]	cell division protein FtsZ	K03531	4	7	3	9	0
Cellular Processes	Cell Growth and Death	04112 Cell cycle - Caulobacter [PATH:ko04112]	GcrA cell cycle regulator	K13583	0	1	0	0	0
Cellular Processes	Cell Growth and Death	04112 Cell cycle - Caulobacter [PATH:ko04112]	modification methylase [EC:2.1.1.72]	K13581	2	2	0	0	0
Cellular Processes	Cell Growth and Death	04112 Cell cycle - Caulobacter [PATH:ko04112]	pilus assembly protein Flp/PilA	K02651	0	0	0	6	0
Cellular Processes	Cell Growth and Death	04112 Cell cycle - Caulobacter [PATH:ko04112]	regulator of sigma E protease [EC:3.4.24.-]	K11749	0	0	0	0	3

Level 1	Level 2	Level 3	Function	KEGG ID	Sequence Reads				
					Day17	Day24	Day31	Day38	Bulk Soil
Cellular Processes	Cell Growth and Death	04112 Cell cycle - Caulobacter [PATH:ko04112]	replicative DNA helicase [EC:3.6.4.12]	K02314	0	0	0	2	6
Cellular Processes	Cell Growth and Death	04112 Cell cycle - Caulobacter [PATH:ko04112]	two-component system, cell cycle response regulator CpdR	K13589	1	0	0	0	0
Cellular Processes	Cell Growth and Death	04112 Cell cycle - Caulobacter [PATH:ko04112]	two-component system, cell cycle response regulator CtrA	K13584	1	0	0	0	0
Cellular Processes	Cell Growth and Death	04112 Cell cycle - Caulobacter [PATH:ko04112]	two-component system, cell cycle response regulator DivK	K11443	0	2	0	0	0
Cellular Processes	Cell Growth and Death	04112 Cell cycle - Caulobacter [PATH:ko04112]	two-component system, cell cycle sensor histidine kinase	K07716	0	2	0	0	3
Cellular Processes	Cell Growth and Death	04112 Cell cycle - Caulobacter [PATH:ko04112]	two-component system, cell cycle sensor histidine kinase and	K13587	1	0	0	0	4
Cellular Processes	Cell Growth and Death	04112 Cell cycle - Caulobacter [PATH:ko04112]	UDP-N-acetylglucosamine--N-acetylmuramyl-(pentapeptide)	K02563	0	3	1	0	0
Cellular Processes	Cell Growth and Death	04113 Meiosis - yeast [PATH:ko04113]	adenylate cyclase [EC:4.6.1.1]	K01768	3	0	0	10	0
Cellular Processes	Cell Growth and Death	04113 Meiosis - yeast [PATH:ko04113]	serine/threonine-protein kinase RIM15 [EC:2.7.11.1]	K12767	0	0	1	0	0
Cellular Processes	Cell Growth and Death	04114 Oocyte meiosis [PATH:ko04114]	protein phosphatase 2 (formerly 2A), regulatory subunit B'	K11584	0	0	0	1	0
Cellular Processes	Cell Growth and Death	04115 p53 signaling pathway [PATH:ko04115]	ribonucleoside-diphosphate reductase subunit M2	K10808	0	0	0	1	0
Cellular Processes	Cell Motility	02030 Bacterial chemotaxis [PATH:ko02030]	chemotaxis protein methyltransferase CheR [EC:2.1.1.80]	K00575	0	0	0	0	3
Cellular Processes	Cell Motility	02030 Bacterial chemotaxis [PATH:ko02030]	purine-binding chemotaxis protein CheW	K03408	0	0	0	2	0

Level 1	Level 2	Level 3	Function	KEGG ID	Sequence Reads				
					Day17	Day24	Day31	Day38	Bulk Soil
Cellular Processes	Cell Motility	02030 Bacterial chemotaxis [PATH:ko02030]	ribose transport system substrate-binding protein	K10439	3	0	0	2	2
Cellular Processes	Cell Motility	02030 Bacterial chemotaxis [PATH:ko02030]	two-component system, chemotaxis family, CheB/CheR fusion	K13924	0	0	0	7	6
Cellular Processes	Cell Motility	02030 Bacterial chemotaxis [PATH:ko02030]	two-component system, chemotaxis family, response regulator	K03412	3	0	0	0	0
Cellular Processes	Cell Motility	02030 Bacterial chemotaxis [PATH:ko02030]	two-component system, chemotaxis family, response regulator	K03413	0	0	3	2	1
Cellular Processes	Cell Motility	02030 Bacterial chemotaxis [PATH:ko02030]	two-component system, chemotaxis family, sensor kinase CheA	K03407	0	6	0	7	0
Cellular Processes	Cell Motility	02040 Flagellar assembly [PATH:ko02040]	chemotaxis protein MotA	K02556	0	1	0	0	0
Cellular Processes	Cell Motility	02040 Flagellar assembly [PATH:ko02040]	chemotaxis protein MotB	K02557	0	0	0	4	2
Cellular Processes	Cell Motility	02040 Flagellar assembly [PATH:ko02040]	flagellar basal-body rod modification protein FlgD	K02389	1	3	0	0	0
Cellular Processes	Cell Motility	02040 Flagellar assembly [PATH:ko02040]	flagellar basal-body rod protein FlgB	K02387	0	0	0	0	1
Cellular Processes	Cell Motility	02040 Flagellar assembly [PATH:ko02040]	flagellar basal-body rod protein FlgC	K02388	0	0	1	0	1
Cellular Processes	Cell Motility	02040 Flagellar assembly [PATH:ko02040]	flagellar biosynthesis protein FlhA	K02400	1	0	1	2	2
Cellular Processes	Cell Motility	02040 Flagellar assembly [PATH:ko02040]	flagellar biosynthetic protein FlhB	K02401	0	0	0	1	0
Cellular Processes	Cell Motility	02040 Flagellar assembly [PATH:ko02040]	flagellar biosynthetic protein FliP	K02419	0	1	0	4	2

Level 1	Level 2	Level 3	Function	KEGG ID	Sequence Reads				
					Day17	Day24	Day31	Day38	Bulk Soil
Cellular Processes	Cell Motility	02040 Flagellar assembly [PATH:ko02040]	flagellar hook-associated protein 1 FlgK	K02396	0	0	0	0	4
Cellular Processes	Cell Motility	02040 Flagellar assembly [PATH:ko02040]	flagellar L-ring protein precursor FlgH	K02393	0	1	0	0	0
Cellular Processes	Cell Motility	02040 Flagellar assembly [PATH:ko02040]	flagellar motor switch protein FliG	K02410	0	2	0	0	2
Cellular Processes	Cell Motility	02040 Flagellar assembly [PATH:ko02040]	flagellar motor switch protein FliM	K02416	0	0	0	1	0
Cellular Processes	Cell Motility	02040 Flagellar assembly [PATH:ko02040]	flagellar motor switch protein FliN/FliY	K02417	0	2	1	3	1
Cellular Processes	Cell Motility	02040 Flagellar assembly [PATH:ko02040]	flagellar M-ring protein FliF	K02409	0	0	0	1	0
Cellular Processes	Cell Motility	02040 Flagellar assembly [PATH:ko02040]	flagellar P-ring protein precursor FlgI	K02394	2	0	0	0	0
Cellular Processes	Cell Motility	02040 Flagellar assembly [PATH:ko02040]	flagellar protein FliO/FliZ	K02418	0	0	0	0	1
Cellular Processes	Cell Motility	02040 Flagellar assembly [PATH:ko02040]	flagellar protein FliS	K02422	2	0	0	0	0
Cellular Processes	Cell Motility	02040 Flagellar assembly [PATH:ko02040]	flagellum-specific ATP synthase [EC:3.6.3.14]	K02412	0	2	0	1	0
Cellular Processes	Transport and Catabolism	04142 Lysosome [PATH:ko04142]	arylsulfatase A [EC:3.1.6.8]	K01134	0	0	1	0	0
Cellular Processes	Transport and Catabolism	04142 Lysosome [PATH:ko04142]	hexosaminidase [EC:3.2.1.52]	K12373	1	0	0	0	0
Cellular Processes	Transport and Catabolism	04144 Endocytosis [PATH:ko04144]	ESCRT-II complex subunit VPS25	K12189	0	0	1	0	0
Cellular Processes	Transport and Catabolism	04146 Peroxisome [PATH:ko04146]	alanine-glyoxylate transaminase / serine-glyoxylate	K00830	0	0	0	0	6

Level 1	Level 2	Level 3	Function	KEGG ID	Sequence Reads				
					Day17	Day24	Day31	Day38	Bulk Soil
Cellular Processes	Transport and Catabolism	04146 Peroxisome [PATH:ko04146]	alpha-methylacyl-CoA racemase [EC:5.1.99.4]	K01796	1	0	0	0	4
Cellular Processes	Transport and Catabolism	04146 Peroxisome [PATH:ko04146]	ATP-binding cassette, subfamily D (ALD), member 4	K05678	1	0	0	0	0
Cellular Processes	Transport and Catabolism	04146 Peroxisome [PATH:ko04146]	D-amino-acid oxidase [EC:1.4.3.3]	K00273	0	0	0	0	1
Cellular Processes	Transport and Catabolism	04146 Peroxisome [PATH:ko04146]	isocitrate dehydrogenase [EC:1.1.1.42]	K00031	4	3	2	6	7
Cellular Processes	Transport and Catabolism	04146 Peroxisome [PATH:ko04146]	peroxin-5	K13342	0	0	0	1	0
Environmental Information Processing	Membrane Transport	02010 ABC transporters [PATH:ko02010]	antibiotic transport system permease protein	K09686	3	0	0	6	0
Environmental Information Processing	Membrane Transport	02010 ABC transporters [PATH:ko02010]	ATP-binding cassette, subfamily C, bacterial	K06148	0	0	4	0	7
Environmental Information Processing	Membrane Transport	02010 ABC transporters [PATH:ko02010]	bicarbonate transport system ATP-binding protein	K11952	0	0	0	1	0
Environmental Information Processing	Membrane Transport	02010 ABC transporters [PATH:ko02010]	bicarbonate transport system ATP-binding protein	K11953	1	0	0	0	0
Environmental Information Processing	Membrane Transport	02010 ABC transporters [PATH:ko02010]	bicarbonate transport system substrate-binding protein	K11950	0	0	1	0	0
Environmental Information Processing	Membrane Transport	02010 ABC transporters [PATH:ko02010]	branched-chain amino acid transport system ATP-binding	K01996	6	0	0	0	0
Environmental Information Processing	Membrane Transport	02010 ABC transporters [PATH:ko02010]	branched-chain amino acid transport system permease protein	K01997	4	0	0	26	0
Environmental Information Processing	Membrane Transport	02010 ABC transporters [PATH:ko02010]	branched-chain amino acid transport system permease protein	K01998	0	12	0	0	26
Environmental Information Processing	Membrane Transport	02010 ABC transporters [PATH:ko02010]	branched-chain amino acid transport system substrate-binding	K01999	15	18	0	24	0

Level 1	Level 2	Level 3	Function	KEGG ID	Sequence Reads				
					Day17	Day24	Day31	Day38	Bulk Soil
Environmental Information Processing	Membrane Transport	02010 ABC transporters [PATH:ko02010]	cell division transport system ATP-binding protein	K09812	0	1	1	4	5
Environmental Information Processing	Membrane Transport	02010 ABC transporters [PATH:ko02010]	cell division transport system permease protein	K09811	0	1	0	2	0
Environmental Information Processing	Membrane Transport	02010 ABC transporters [PATH:ko02010]	cellobiose transport system ATP-binding protein	K10243	0	1	0	0	2
Environmental Information Processing	Membrane Transport	02010 ABC transporters [PATH:ko02010]	cellobiose transport system permease protein	K10241	0	1	0	0	0
Environmental Information Processing	Membrane Transport	02010 ABC transporters [PATH:ko02010]	cellobiose transport system substrate-binding protein	K10240	0	0	1	0	0
Environmental Information Processing	Membrane Transport	02010 ABC transporters [PATH:ko02010]	cobalt/nickel transport system permease protein	K02007	0	2	0	0	1
Environmental Information Processing	Membrane Transport	02010 ABC transporters [PATH:ko02010]	dipeptide transport system permease protein	K12369	0	0	3	1	0
Environmental Information Processing	Membrane Transport	02010 ABC transporters [PATH:ko02010]	D-methionine transport system ATP-binding protein	K02071	0	0	1	1	0
Environmental Information Processing	Membrane Transport	02010 ABC transporters [PATH:ko02010]	D-methionine transport system permease protein	K02072	0	0	0	0	1
Environmental Information Processing	Membrane Transport	02010 ABC transporters [PATH:ko02010]	D-xylose transport system permease protein	K10544	0	0	2	2	0
Environmental Information Processing	Membrane Transport	02010 ABC transporters [PATH:ko02010]	D-xylose transport system substrate-binding protein	K10543	0	0	0	6	0
Environmental Information Processing	Membrane Transport	02010 ABC transporters [PATH:ko02010]	fructose transport system ATP-binding protein	K10554	1	1	0	1	0
Environmental Information Processing	Membrane Transport	02010 ABC transporters [PATH:ko02010]	fructose transport system permease protein	K10553	0	0	1	0	0

Level 1	Level 2	Level 3	Function	KEGG ID	Sequence Reads				
					Day17	Day24	Day31	Day38	Bulk Soil
Environmental Information Processing	Membrane Transport	02010 ABC transporters [PATH:ko02010]	general L-amino acid transport system ATP-binding protein	K09972	2	0	0	0	0
Environmental Information Processing	Membrane Transport	02010 ABC transporters [PATH:ko02010]	general L-amino acid transport system permease protein	K09970	0	0	3	0	0
Environmental Information Processing	Membrane Transport	02010 ABC transporters [PATH:ko02010]	general L-amino acid transport system permease protein	K09971	0	0	0	2	0
Environmental Information Processing	Membrane Transport	02010 ABC transporters [PATH:ko02010]	glutamate transport system ATP-binding protein [EC:3.6.3.-]	K10008	1	2	0	1	0
Environmental Information Processing	Membrane Transport	02010 ABC transporters [PATH:ko02010]	glutamate/aspartate transport system ATP-binding protein	K10004	0	0	1	0	0
Environmental Information Processing	Membrane Transport	02010 ABC transporters [PATH:ko02010]	glutamate/aspartate transport system permease protein	K10003	1	0	1	0	0
Environmental Information Processing	Membrane Transport	02010 ABC transporters [PATH:ko02010]	glutamate/aspartate transport system substrate-binding	K10001	0	0	0	0	1
Environmental Information Processing	Membrane Transport	02010 ABC transporters [PATH:ko02010]	glutathione transport system ATP-binding protein	K13892	0	0	0	0	2
Environmental Information Processing	Membrane Transport	02010 ABC transporters [PATH:ko02010]	glycine betaine/proline transport system ATP-binding protein	K02000	0	2	0	1	0
Environmental Information Processing	Membrane Transport	02010 ABC transporters [PATH:ko02010]	glycine betaine/proline transport system permease protein	K02001	0	0	2	0	0
Environmental Information Processing	Membrane Transport	02010 ABC transporters [PATH:ko02010]	iron complex transport system ATP-binding protein	K02013	0	0	0	3	0
Environmental Information Processing	Membrane Transport	02010 ABC transporters [PATH:ko02010]	iron complex transport system permease protein	K02015	0	2	0	0	0
Environmental Information Processing	Membrane Transport	02010 ABC transporters [PATH:ko02010]	iron complex transport system substrate-binding protein	K02016	0	0	0	0	5

Level 1	Level 2	Level 3	Function	KEGG ID	Sequence Reads				
					Day17	Day24	Day31	Day38	Bulk Soil
Environmental Information Processing	Membrane Transport	02010 ABC transporters [PATH:ko02010]	iron(III) transport system ATP-binding protein [EC:3.6.3.30]	K02010	0	0	2	4	0
Environmental Information Processing	Membrane Transport	02010 ABC transporters [PATH:ko02010]	iron(III) transport system permease protein	K02011	1	0	0	0	0
Environmental Information Processing	Membrane Transport	02010 ABC transporters [PATH:ko02010]	iron(III) transport system substrate-binding protein	K02012	2	2	0	0	0
Environmental Information Processing	Membrane Transport	02010 ABC transporters [PATH:ko02010]	lactose/L-arabinose transport system substrate-binding	K10188	0	0	0	1	0
Environmental Information Processing	Membrane Transport	02010 ABC transporters [PATH:ko02010]	lipopolysaccharide export system ATP-binding protein	K06861	1	0	2	0	0
Environmental Information Processing	Membrane Transport	02010 ABC transporters [PATH:ko02010]	lipopolysaccharide transport system permease protein	K09690	0	0	2	0	1
Environmental Information Processing	Membrane Transport	02010 ABC transporters [PATH:ko02010]	lipoprotein-releasing system permease protein	K09808	0	0	0	1	2
Environmental Information Processing	Membrane Transport	02010 ABC transporters [PATH:ko02010]	maltose/maltodextrin transport system ATP-binding protein	K10112	2	0	0	0	5
Environmental Information Processing	Membrane Transport	02010 ABC transporters [PATH:ko02010]	manganese/zinc/iron transport system permease protein	K11708	0	0	0	0	1
Environmental Information Processing	Membrane Transport	02010 ABC transporters [PATH:ko02010]	methyl-galactoside transport system ATP-binding protein	K10542	3	0	0	0	0
Environmental Information Processing	Membrane Transport	02010 ABC transporters [PATH:ko02010]	microcin C transport system ATP-binding protein	K13896	1	3	0	1	0
Environmental Information Processing	Membrane Transport	02010 ABC transporters [PATH:ko02010]	microcin C transport system permease protein	K13894	0	0	0	3	0
Environmental Information Processing	Membrane Transport	02010 ABC transporters [PATH:ko02010]	microcin C transport system substrate-binding protein	K13893	0	0	0	0	1

Level 1	Level 2	Level 3	Function	KEGG ID	Sequence Reads				
					Day17	Day24	Day31	Day38	Bulk Soil
Environmental Information Processing	Membrane Transport	02010 ABC transporters [PATH:ko02010]	molybdate transport system ATP-binding protein [EC:3.6.3.29]	K02017	0	0	0	1	0
Environmental Information Processing	Membrane Transport	02010 ABC transporters [PATH:ko02010]	molybdate transport system permease protein	K02018	0	2	0	0	0
Environmental Information Processing	Membrane Transport	02010 ABC transporters [PATH:ko02010]	molybdate transport system substrate-binding protein	K02020	0	4	1	0	5
Environmental Information Processing	Membrane Transport	02010 ABC transporters [PATH:ko02010]	multiple sugar transport system permease protein	K10118	0	0	0	1	0
Environmental Information Processing	Membrane Transport	02010 ABC transporters [PATH:ko02010]	neutral amino acid transport system permease protein	K11956	0	0	0	1	0
Environmental Information Processing	Membrane Transport	02010 ABC transporters [PATH:ko02010]	neutral amino acid transport system substrate-binding	K11954	1	0	0	0	0
Environmental Information Processing	Membrane Transport	02010 ABC transporters [PATH:ko02010]	oligogalacturonide transport system permease protein	K10193	1	0	0	0	0
Environmental Information Processing	Membrane Transport	02010 ABC transporters [PATH:ko02010]	osmoprotectant transport system substrate-binding protein	K05845	0	2	0	0	0
Environmental Information Processing	Membrane Transport	02010 ABC transporters [PATH:ko02010]	peptide/nickel transport system ATP-binding protein	K02031	0	12	0	0	0
Environmental Information Processing	Membrane Transport	02010 ABC transporters [PATH:ko02010]	peptide/nickel transport system ATP-binding protein	K02032	0	0	0	0	15
Environmental Information Processing	Membrane Transport	02010 ABC transporters [PATH:ko02010]	peptide/nickel transport system permease protein	K02033	0	0	5	0	0
Environmental Information Processing	Membrane Transport	02010 ABC transporters [PATH:ko02010]	peptide/nickel transport system permease protein	K02034	0	13	0	12	0
Environmental Information Processing	Membrane Transport	02010 ABC transporters [PATH:ko02010]	peptide/nickel transport system substrate-binding protein	K02035	0	22	0	0	0

Level 1	Level 2	Level 3	Function	KEGG ID	Sequence Reads				
					Day17	Day24	Day31	Day38	Bulk Soil
Environmental Information Processing	Membrane Transport	02010 ABC transporters [PATH:ko02010]	phosphate transport system ATP-binding protein [EC:3.6.3.27]	K02036	4	0	0	1	0
Environmental Information Processing	Membrane Transport	02010 ABC transporters [PATH:ko02010]	phosphate transport system permease protein	K02037	2	0	1	0	0
Environmental Information Processing	Membrane Transport	02010 ABC transporters [PATH:ko02010]	phosphate transport system permease protein	K02038	0	0	0	4	4
Environmental Information Processing	Membrane Transport	02010 ABC transporters [PATH:ko02010]	phosphonate transport system permease protein	K02042	0	0	2	0	0
Environmental Information Processing	Membrane Transport	02010 ABC transporters [PATH:ko02010]	putative ATP-binding cassette transporter	K06160	0	0	0	0	1
Environmental Information Processing	Membrane Transport	02010 ABC transporters [PATH:ko02010]	putative glutamine transport system ATP-binding protein	K10041	0	0	1	0	0
Environmental Information Processing	Membrane Transport	02010 ABC transporters [PATH:ko02010]	putative multiple sugar transport system ATP-binding protein	K10548	0	0	0	1	0
Environmental Information Processing	Membrane Transport	02010 ABC transporters [PATH:ko02010]	putative multiple sugar transport system permease protein	K10547	1	0	0	0	0
Environmental Information Processing	Membrane Transport	02010 ABC transporters [PATH:ko02010]	putative multiple sugar transport system substrate-binding	K10546	15	1	1	0	0
Environmental Information Processing	Membrane Transport	02010 ABC transporters [PATH:ko02010]	putative phosphonate transport system ATP-binding protein	K05780	0	0	0	1	0
Environmental Information Processing	Membrane Transport	02010 ABC transporters [PATH:ko02010]	putative tungstate transport system permease protein	K05773	0	0	0	0	1
Environmental Information Processing	Membrane Transport	02010 ABC transporters [PATH:ko02010]	putative tungstate transport system substrate-binding	K05772	0	0	1	0	0
Environmental Information Processing	Membrane Transport	02010 ABC transporters [PATH:ko02010]	putrescine transport system ATP-binding protein	K11076	0	2	1	0	0

Level 1	Level 2	Level 3	Function	KEGG ID	Sequence Reads				
					Day17	Day24	Day31	Day38	Bulk Soil
Environmental Information Processing	Membrane Transport	02010 ABC transporters [PATH:ko02010]	putrescine transport system permease protein	K11074	0	0	1	0	0
Environmental Information Processing	Membrane Transport	02010 ABC transporters [PATH:ko02010]	ribose transport system permease protein	K10440	0	0	0	7	3
Environmental Information Processing	Membrane Transport	02010 ABC transporters [PATH:ko02010]	sn-glycerol 3-phosphate transport system ATP-binding protein	K05816	0	2	0	0	0
Environmental Information Processing	Membrane Transport	02010 ABC transporters [PATH:ko02010]	sn-glycerol 3-phosphate transport system permease protein	K05814	0	3	0	0	0
Environmental Information Processing	Membrane Transport	02010 ABC transporters [PATH:ko02010]	sn-glycerol 3-phosphate transport system substrate-binding	K05813	0	0	0	0	4
Environmental Information Processing	Membrane Transport	02010 ABC transporters [PATH:ko02010]	sorbitol/mannitol transport system permease protein	K10229	1	0	0	1	0
Environmental Information Processing	Membrane Transport	02010 ABC transporters [PATH:ko02010]	sorbitol/mannitol transport system substrate-binding protein	K10227	1	1	0	1	0
Environmental Information Processing	Membrane Transport	02010 ABC transporters [PATH:ko02010]	spermidine/putrescine transport system ATP-binding protein	K11072	1	0	0	0	0
Environmental Information Processing	Membrane Transport	02010 ABC transporters [PATH:ko02010]	spermidine/putrescine transport system permease protein	K11070	0	3	0	0	0
Environmental Information Processing	Membrane Transport	02010 ABC transporters [PATH:ko02010]	spermidine/putrescine transport system permease protein	K11071	2	0	0	1	0
Environmental Information Processing	Membrane Transport	02010 ABC transporters [PATH:ko02010]	sulfate transport system ATP-binding protein [EC:3.6.3.25]	K02045	0	0	0	0	1
Environmental Information Processing	Membrane Transport	02010 ABC transporters [PATH:ko02010]	sulfate transport system permease protein	K02046	0	3	0	2	1
Environmental Information Processing	Membrane Transport	02010 ABC transporters [PATH:ko02010]	sulfate transport system permease protein	K02047	0	0	2	0	0

Level 1	Level 2	Level 3	Function	KEGG ID	Sequence Reads				
					Day17	Day24	Day31	Day38	Bulk Soil
Environmental Information Processing	Membrane Transport	02010 ABC transporters [PATH:ko02010]	sulfonate/nitrate/taurine transport system ATP-binding	K02049	0	0	0	0	13
Environmental Information Processing	Membrane Transport	02010 ABC transporters [PATH:ko02010]	sulfonate/nitrate/taurine transport system permease protein	K02050	0	0	2	6	0
Environmental Information Processing	Membrane Transport	02010 ABC transporters [PATH:ko02010]	sulfonate/nitrate/taurine transport system substrate-binding	K02051	5	0	5	0	9
Environmental Information Processing	Membrane Transport	02010 ABC transporters [PATH:ko02010]	thiamine transport system permease protein	K02063	1	0	0	0	0
Environmental Information Processing	Membrane Transport	02010 ABC transporters [PATH:ko02010]	thiamine transport system substrate-binding protein	K02064	0	0	0	1	0
Environmental Information Processing	Membrane Transport	02010 ABC transporters [PATH:ko02010]	trehalose/maltose transport system permease protein	K10238	0	0	0	0	1
Environmental Information Processing	Membrane Transport	02060 Phosphotransferase system (PTS) [PATH:ko02060]	PTS system, mannitol-specific IIB component [EC:2.7.1.69]	K02799	0	0	1	0	0
Environmental Information Processing	Membrane Transport	02060 Phosphotransferase system (PTS) [PATH:ko02060]	PTS system, mannitol-specific IIC component	K02800	0	0	1	0	0
Environmental Information Processing	Membrane Transport	02060 Phosphotransferase system (PTS) [PATH:ko02060]	PTS system, mannose-specific IIA component [EC:2.7.1.69]	K02793	0	0	0	0	1
Environmental Information Processing	Membrane Transport	02060 Phosphotransferase system (PTS) [PATH:ko02060]	PTS system, N-acetylglucosamine-specific IIB component	K02803	0	0	0	0	1
Environmental Information Processing	Membrane Transport	02060 Phosphotransferase system (PTS) [PATH:ko02060]	PTS system, N-acetylglucosamine-specific IIC component	K02804	0	0	0	0	1

Level 1	Level 2	Level 3	Function	KEGG ID	Sequence Reads				
					Day17	Day24	Day31	Day38	Bulk Soil
Environmental Information Processing	Membrane Transport	03070 Bacterial secretion system [PATH:ko03070]	ATP-binding cassette, subfamily B, bacterial HlyB/CyaB	K11004	0	0	0	1	0
Environmental Information Processing	Membrane Transport	03070 Bacterial secretion system [PATH:ko03070]	fused signal recognition particle receptor	K03110	3	7	4	0	9
Environmental Information Processing	Membrane Transport	03070 Bacterial secretion system [PATH:ko03070]	general secretion pathway protein D	K02453	0	0	3	0	0
Environmental Information Processing	Membrane Transport	03070 Bacterial secretion system [PATH:ko03070]	general secretion pathway protein E	K02454	0	0	4	3	6
Environmental Information Processing	Membrane Transport	03070 Bacterial secretion system [PATH:ko03070]	general secretion pathway protein F	K02455	0	2	0	0	1
Environmental Information Processing	Membrane Transport	03070 Bacterial secretion system [PATH:ko03070]	general secretion pathway protein G	K02456	0	0	0	0	2
Environmental Information Processing	Membrane Transport	03070 Bacterial secretion system [PATH:ko03070]	general secretion pathway protein I	K02458	0	0	1	0	0
Environmental Information Processing	Membrane Transport	03070 Bacterial secretion system [PATH:ko03070]	general secretion pathway protein L	K02461	0	1	0	0	0
Environmental Information Processing	Membrane Transport	03070 Bacterial secretion system [PATH:ko03070]	preprotein translocase subunit SecA	K03070	12	7	13	8	10
Environmental Information Processing	Membrane Transport	03070 Bacterial secretion system [PATH:ko03070]	preprotein translocase subunit SecB	K03071	0	2	0	0	0
Environmental Information Processing	Membrane Transport	03070 Bacterial secretion system [PATH:ko03070]	preprotein translocase subunit SecE	K03073	0	0	1	0	0
Environmental Information Processing	Membrane Transport	03070 Bacterial secretion system [PATH:ko03070]	preprotein translocase subunit SecF	K03074	0	0	1	0	0
Environmental Information Processing	Membrane Transport	03070 Bacterial secretion system [PATH:ko03070]	preprotein translocase subunit SecG	K03075	3	1	0	0	0

Level 1	Level 2	Level 3	Function	KEGG ID	Sequence Reads				
					Day17	Day24	Day31	Day38	Bulk Soil
Environmental Information Processing	Membrane Transport	03070 Bacterial secretion system [PATH:ko03070]	preprotein translocase subunit SecY	K03076	11	0	2	3	2
Environmental Information Processing	Membrane Transport	03070 Bacterial secretion system [PATH:ko03070]	preprotein translocase subunit YajC	K03210	0	0	0	2	0
Environmental Information Processing	Membrane Transport	03070 Bacterial secretion system [PATH:ko03070]	preprotein translocase subunit YidC	K03217	2	6	6	0	0
Environmental Information Processing	Membrane Transport	03070 Bacterial secretion system [PATH:ko03070]	SecD/SecF fusion protein	K12257	0	3	0	0	1
Environmental Information Processing	Membrane Transport	03070 Bacterial secretion system [PATH:ko03070]	sec-independent protein translocase protein TatA	K03116	0	0	0	1	4
Environmental Information Processing	Membrane Transport	03070 Bacterial secretion system [PATH:ko03070]	sec-independent protein translocase protein TatB	K03117	1	0	0	1	0
Environmental Information Processing	Membrane Transport	03070 Bacterial secretion system [PATH:ko03070]	sec-independent protein translocase protein TatC	K03118	1	0	0	2	0
Environmental Information Processing	Membrane Transport	03070 Bacterial secretion system [PATH:ko03070]	serine/threonine-protein kinase PpkA [EC:2.7.11.1]	K11912	0	0	1	0	0
Environmental Information Processing	Membrane Transport	03070 Bacterial secretion system [PATH:ko03070]	signal recognition particle subunit SRP54	K03106	2	7	6	5	7
Environmental Information Processing	Membrane Transport	03070 Bacterial secretion system [PATH:ko03070]	type IV secretion system protein VirB11	K03196	0	1	0	0	0
Environmental Information Processing	Membrane Transport	03070 Bacterial secretion system [PATH:ko03070]	type IV secretion system protein VirB3	K03198	0	1	0	0	0
Environmental Information Processing	Membrane Transport	03070 Bacterial secretion system [PATH:ko03070]	type IV secretion system protein VirB4	K03199	0	2	0	0	0
Environmental Information Processing	Membrane Transport	03070 Bacterial secretion system [PATH:ko03070]	type IV secretion system protein VirD4	K03205	0	1	0	1	1

Level 1	Level 2	Level 3	Function	KEGG ID	Sequence Reads				
					Day17	Day24	Day31	Day38	Bulk Soil
Environmental Information Processing	Membrane Transport	03070 Bacterial secretion system [PATH:ko03070]	type VI secretion system protein VasG	K11907	2	0	0	1	0
Environmental Information Processing	Membrane Transport	03070 Bacterial secretion system [PATH:ko03070]	type VI secretion system secreted protein Hcp	K11903	0	1	0	2	0
Environmental Information Processing	Signal Transduction	02020 Two-component system [PATH:ko02020]	[protein-PII] uridylyltransferase [EC:2.7.7.59]	K00990	0	0	3	0	5
Environmental Information Processing	Signal Transduction	02020 Two-component system [PATH:ko02020]	acetyl-CoA C-acetyltransferase [EC:2.3.1.9]	K00626	13	23	0	6	0
Environmental Information Processing	Signal Transduction	02020 Two-component system [PATH:ko02020]	aerobic C4-dicarboxylate transport protein	K11103	4	3	8	0	3
Environmental Information Processing	Signal Transduction	02020 Two-component system [PATH:ko02020]	alkaline phosphatase D [EC:3.1.3.1]	K01113	0	0	2	0	3
Environmental Information Processing	Signal Transduction	02020 Two-component system [PATH:ko02020]	beta-lactamase [EC:3.5.2.6]	K01467	2	2	0	0	1
Environmental Information Processing	Signal Transduction	02020 Two-component system [PATH:ko02020]	C4-dicarboxylate transporter, DctM subunit	K11690	0	0	0	2	0
Environmental Information Processing	Signal Transduction	02020 Two-component system [PATH:ko02020]	C4-dicarboxylate transporter, DctQ subunit	K11689	0	0	1	0	0
Environmental Information Processing	Signal Transduction	02020 Two-component system [PATH:ko02020]	cation efflux system protein involved in nickel and cobalt	K11326	0	0	1	0	0
Environmental Information Processing	Signal Transduction	02020 Two-component system [PATH:ko02020]	chromosomal replication initiator protein	K02313	0	5	3	6	0
Environmental Information Processing	Signal Transduction	02020 Two-component system [PATH:ko02020]	citrate lyase subunit beta / citryl-CoA lyase [EC:4.1.3.6	K01644	0	0	0	0	1
Environmental Information Processing	Signal Transduction	02020 Two-component system [PATH:ko02020]	Cu(I)/Ag(I) efflux system membrane protein CusA	K07787	1	0	0	0	2

Level 1	Level 2	Level 3	Function	KEGG ID	Sequence Reads				
					Day17	Day24	Day31	Day38	Bulk Soil
Environmental Information Processing	Signal Transduction	02020 Two-component system [PATH:ko02020]	Cu(I)/Ag(I) efflux system membrane protein CusB	K07798	4	0	0	0	0
Environmental Information Processing	Signal Transduction	02020 Two-component system [PATH:ko02020]	cytochrome c oxidase subunit XV assembly protein	K02259	0	1	0	0	0
Environmental Information Processing	Signal Transduction	02020 Two-component system [PATH:ko02020]	glutamine synthetase [EC:6.3.1.2]	K01915	9	18	19	17	10
Environmental Information Processing	Signal Transduction	02020 Two-component system [PATH:ko02020]	K+-transporting ATPase ATPase B chain [EC:3.6.3.12]	K01547	0	1	2	2	2
Environmental Information Processing	Signal Transduction	02020 Two-component system [PATH:ko02020]	malate dehydrogenase (oxaloacetate-decarboxylating)	K00027	1	0	0	3	0
Environmental Information Processing	Signal Transduction	02020 Two-component system [PATH:ko02020]	malate:Na+ symporter	K11616	0	1	0	0	0
Environmental Information Processing	Signal Transduction	02020 Two-component system [PATH:ko02020]	methyl-accepting chemotaxis protein	K03406	0	0	8	0	19
Environmental Information Processing	Signal Transduction	02020 Two-component system [PATH:ko02020]	Nif-specific regulatory protein	K02584	0	0	0	0	4
Environmental Information Processing	Signal Transduction	02020 Two-component system [PATH:ko02020]	nitrate reductase 1, alpha subunit [EC:1.7.99.4]	K00370	4	0	2	2	0
Environmental Information Processing	Signal Transduction	02020 Two-component system [PATH:ko02020]	nitrate reductase 1, beta subunit [EC:1.7.99.4]	K00371	1	2	0	1	1
Environmental Information Processing	Signal Transduction	02020 Two-component system [PATH:ko02020]	nitrate reductase 1, delta subunit [EC:1.7.99.4]	K00373	0	0	0	0	1
Environmental Information Processing	Signal Transduction	02020 Two-component system [PATH:ko02020]	nitrogen regulatory protein P-II 1	K04751	3	2	2	2	1
Environmental Information Processing	Signal Transduction	02020 Two-component system [PATH:ko02020]	omega-6 fatty acid desaturase (delta-12 desaturase)	K10255	1	0	0	0	0

Level 1	Level 2	Level 3	Function	KEGG ID	Sequence Reads				
					Day17	Day24	Day31	Day38	Bulk Soil
Environmental Information Processing	Signal Transduction	02020 Two-component system [PATH:ko02020]	phosphate transport system substrate-binding protein	K02040	5	5	2	0	0
Environmental Information Processing	Signal Transduction	02020 Two-component system [PATH:ko02020]	putative serine protease PepD [EC:3.4.21.-]	K08372	0	0	0	0	2
Environmental Information Processing	Signal Transduction	02020 Two-component system [PATH:ko02020]	putative tricarboxylic transport membrane protein	K07793	8	5	1	7	0
Environmental Information Processing	Signal Transduction	02020 Two-component system [PATH:ko02020]	putative tricarboxylic transport membrane protein	K07794	0	0	0	0	1
Environmental Information Processing	Signal Transduction	02020 Two-component system [PATH:ko02020]	RND superfamily, multidrug transport protein MdtB	K07788	0	11	0	0	0
Environmental Information Processing	Signal Transduction	02020 Two-component system [PATH:ko02020]	RND superfamily, multidrug transport protein MdtC	K07789	0	1	0	5	0
Environmental Information Processing	Signal Transduction	02020 Two-component system [PATH:ko02020]	short-chain fatty acids transporter	K02106	0	0	0	0	1
Environmental Information Processing	Signal Transduction	02020 Two-component system [PATH:ko02020]	sodium transport system ATP-binding protein	K09697	0	0	0	1	0
Environmental Information Processing	Signal Transduction	02020 Two-component system [PATH:ko02020]	tetrathionate reductase subunit B	K08358	0	0	1	0	0
Environmental Information Processing	Signal Transduction	02020 Two-component system [PATH:ko02020]	twitching motility protein PilJ	K02660	0	0	0	2	0
Environmental Information Processing	Signal Transduction	02020 Two-component system [PATH:ko02020]	two-component system, cell cycle sensor kinase and response	K02489	2	0	0	0	0
Environmental Information Processing	Signal Transduction	02020 Two-component system [PATH:ko02020]	two-component system, chemotaxis family, sensor kinase Cph1	K11354	1	0	0	1	0
Environmental Information Processing	Signal Transduction	02020 Two-component system [PATH:ko02020]	two-component system, LuxR family, sensor histidine kinase	K11711	0	1	0	0	0

Level 1	Level 2	Level 3	Function	KEGG ID	Sequence Reads				
					Day17	Day24	Day31	Day38	Bulk Soil
Environmental Information Processing	Signal Transduction	02020 Two-component system [PATH:ko02020]	two-component system, LytT family, response regulator AlgR	K08083	0	1	0	0	0
Environmental Information Processing	Signal Transduction	02020 Two-component system [PATH:ko02020]	two-component system, LytT family, sensor histidine kinase	K08082	0	0	0	1	0
Environmental Information Processing	Signal Transduction	02020 Two-component system [PATH:ko02020]	two-component system, NarL family, sensor histidine kinase	K07778	0	0	0	0	2
Environmental Information Processing	Signal Transduction	02020 Two-component system [PATH:ko02020]	two-component system, NtrC family, C4-dicarboxylate	K10126	1	0	1	0	0
Environmental Information Processing	Signal Transduction	02020 Two-component system [PATH:ko02020]	two-component system, NtrC family, nitrogen regulation	K07708	0	0	0	2	1
Environmental Information Processing	Signal Transduction	02020 Two-component system [PATH:ko02020]	two-component system, NtrC family, nitrogen regulation	K07712	0	3	1	0	0
Environmental Information Processing	Signal Transduction	02020 Two-component system [PATH:ko02020]	two-component system, NtrC family, nitrogen regulation	K13598	1	0	0	0	0
Environmental Information Processing	Signal Transduction	02020 Two-component system [PATH:ko02020]	two-component system, NtrC family, response regulator PilR	K02667	0	0	0	2	0
Environmental Information Processing	Signal Transduction	02020 Two-component system [PATH:ko02020]	two-component system, NtrC family, sensor histidine kinase	K02668	0	0	0	2	1
Environmental Information Processing	Signal Transduction	02020 Two-component system [PATH:ko02020]	two-component system, OmpR family, copper resistance	K07665	1	0	0	0	0
Environmental Information Processing	Signal Transduction	02020 Two-component system [PATH:ko02020]	two-component system, OmpR family, osmolarity sensor	K07638	0	1	0	0	0
Environmental Information Processing	Signal Transduction	02020 Two-component system [PATH:ko02020]	two-component system, OmpR family, phosphate regulon	K07659	0	0	0	3	0
Environmental Information Processing	Signal Transduction	02020 Two-component system [PATH:ko02020]	two-component system, OmpR family, response regulator MtrA	K07670	0	0	0	0	3

Level 1	Level 2	Level 3	Function	KEGG ID	Sequence Reads				
					Day17	Day24	Day31	Day38	Bulk Soil
Environmental Information Processing	Signal Transduction	02020 Two-component system [PATH:ko02020]	two-component system, OmpR family, response regulator NblR	K11332	0	0	0	1	0
Environmental Information Processing	Signal Transduction	02020 Two-component system [PATH:ko02020]	two-component system, OmpR family, response regulator RegX3	K07776	1	2	2	0	0
Environmental Information Processing	Signal Transduction	02020 Two-component system [PATH:ko02020]	two-component system, OmpR family, sensor histidine kinase	K07641	0	0	0	2	0
Environmental Information Processing	Signal Transduction	02020 Two-component system [PATH:ko02020]	two-component system, OmpR family, sensor histidine kinase	K07642	0	0	0	0	3
Environmental Information Processing	Signal Transduction	02020 Two-component system [PATH:ko02020]	two-component system, OmpR family, sensor histidine kinase	K07646	1	0	0	0	0
Environmental Information Processing	Signal Transduction	02020 Two-component system [PATH:ko02020]	two-component system, OmpR family, sensor histidine kinase	K07651	0	1	0	0	0
Environmental Information Processing	Signal Transduction	02020 Two-component system [PATH:ko02020]	type IV pilus assembly protein PilA	K02650	5	4	5	3	3
Environmental Information Processing	Signal Transduction	04011 MAPK signaling pathway - yeast [PATH:ko04011]	lactoylglutathione lyase [EC:4.4.1.5]	K01759	0	0	2	0	1
Environmental Information Processing	Signal Transduction	04070 Phosphatidylinositol signaling system [PATH:ko04070]	diacylglycerol kinase [EC:2.7.1.107]	K00901	0	0	1	0	0
Environmental Information Processing	Signal Transduction	04070 Phosphatidylinositol signaling system [PATH:ko04070]	myo-inositol-1(or 4)-monophosphatase [EC:3.1.3.25]	K01092	2	2	0	0	0
Environmental Information Processing	Signal Transduction	04070 Phosphatidylinositol signaling system [PATH:ko04070]	phosphatidate cytidylyltransferase [EC:2.7.7.41]	K00981	0	0	0	1	0

Level 1	Level 2	Level 3	Function	KEGG ID	Sequence Reads				
					Day17	Day24	Day31	Day38	Bulk Soil
Environmental Information Processing	Signal Transduction	04340 Hedgehog signaling pathway [PATH:ko04340]	casein kinase 1 [EC:2.7.11.1]	K02218	1	0	0	0	0
Genetic Information Processing	Folding, Sorting and Degradation	03018 RNA degradation [PATH:ko03018]	5'-3' exoribonuclease 2 [EC:3.1.13.-]	K12619	1	0	0	0	0
Genetic Information Processing	Folding, Sorting and Degradation	03018 RNA degradation [PATH:ko03018]	ATP-dependent DNA helicase RecQ [EC:3.6.4.12]	K03654	0	0	0	0	10
Genetic Information Processing	Folding, Sorting and Degradation	03018 RNA degradation [PATH:ko03018]	ATP-dependent RNA helicase DeaD [EC:3.6.4.13]	K05592	0	4	0	2	0
Genetic Information Processing	Folding, Sorting and Degradation	03018 RNA degradation [PATH:ko03018]	ATP-dependent RNA helicase RhlE [EC:3.6.4.13]	K11927	6	3	0	4	0
Genetic Information Processing	Folding, Sorting and Degradation	03018 RNA degradation [PATH:ko03018]	enolase [EC:4.2.1.11]	K01689	5	2	3	0	6
Genetic Information Processing	Folding, Sorting and Degradation	03018 RNA degradation [PATH:ko03018]	host factor-I protein	K03666	0	2	1	1	0
Genetic Information Processing	Folding, Sorting and Degradation	03018 RNA degradation [PATH:ko03018]	molecular chaperone DnaK	K04043	25	6	13	15	8
Genetic Information Processing	Folding, Sorting and Degradation	03018 RNA degradation [PATH:ko03018]	PAB-dependent poly(A)-specific ribonuclease subunit 2	K12571	0	0	0	1	0
Genetic Information Processing	Folding, Sorting and Degradation	03018 RNA degradation [PATH:ko03018]	poly(A) polymerase [EC:2.7.7.19]	K00970	0	2	2	0	0
Genetic Information Processing	Folding, Sorting and Degradation	03018 RNA degradation [PATH:ko03018]	polyadenylate-binding protein	K13126	0	0	1	0	0
Genetic Information Processing	Folding, Sorting and Degradation	03018 RNA degradation [PATH:ko03018]	polyphosphate kinase [EC:2.7.4.1]	K00937	0	0	0	4	7
Genetic Information Processing	Folding, Sorting and Degradation	03018 RNA degradation [PATH:ko03018]	polyribonucleotide nucleotidyltransferase [EC:2.7.7.8]	K00962	16	14	0	13	0

Level 1	Level 2	Level 3	Function	KEGG ID	Sequence Reads				
					Day17	Day24	Day31	Day38	Bulk Soil
Genetic Information Processing	Folding, Sorting and Degradation	03018 RNA degradation [PATH:ko03018]	putative (di)nucleoside polyphosphate hydrolase [EC:3.6.1.-]	K08311	0	1	0	0	0
Genetic Information Processing	Folding, Sorting and Degradation	03018 RNA degradation [PATH:ko03018]	ribonuclease E [EC:3.1.26.12]	K08300	0	5	2	0	1
Genetic Information Processing	Folding, Sorting and Degradation	03018 RNA degradation [PATH:ko03018]	ribonuclease R [EC:3.1.-.-]	K12573	1	1	6	7	0
Genetic Information Processing	Folding, Sorting and Degradation	03018 RNA degradation [PATH:ko03018]	transcription termination factor Rho	K03628	6	2	6	5	4
Genetic Information Processing	Folding, Sorting and Degradation	03050 Proteasome [PATH:ko03050]	20S proteasome subunit beta 1 [EC:3.4.25.1]	K02738	0	0	1	0	0
Genetic Information Processing	Folding, Sorting and Degradation	03050 Proteasome [PATH:ko03050]	20S proteasome subunit beta 5 [EC:3.4.25.1]	K02737	0	0	0	1	0
Genetic Information Processing	Folding, Sorting and Degradation	03050 Proteasome [PATH:ko03050]	26S proteasome regulatory subunit N1	K03028	2	0	0	0	0
Genetic Information Processing	Folding, Sorting and Degradation	03050 Proteasome [PATH:ko03050]	26S proteasome regulatory subunit N8	K03038	1	0	0	0	0
Genetic Information Processing	Folding, Sorting and Degradation	03050 Proteasome [PATH:ko03050]	26S proteasome regulatory subunit T2	K03062	1	0	0	0	0
Genetic Information Processing	Folding, Sorting and Degradation	03050 Proteasome [PATH:ko03050]	proteasome alpha subunit [EC:3.4.25.1]	K03432	0	1	0	1	0
Genetic Information Processing	Folding, Sorting and Degradation	03050 Proteasome [PATH:ko03050]	proteasome beta subunit [EC:3.4.25.1]	K03433	0	1	0	0	0
Genetic Information Processing	Folding, Sorting and Degradation	03050 Proteasome [PATH:ko03050]	proteasome-associated ATPase	K13527	1	3	1	4	0
Genetic Information Processing	Folding, Sorting and Degradation	03060 Protein export [PATH:ko03060]	signal peptidase I [EC:3.4.21.89]	K03100	4	1	1	0	0

Level 1	Level 2	Level 3	Function	KEGG ID	Sequence Reads				
					Day17	Day24	Day31	Day38	Bulk Soil
Genetic Information Processing	Folding, Sorting and Degradation	04120 Ubiquitin mediated proteolysis [PATH:ko04120]	baculoviral IAP repeat-containing protein 6 (apollon)	K10586	0	0	1	0	0
Genetic Information Processing	Folding, Sorting and Degradation	04120 Ubiquitin mediated proteolysis [PATH:ko04120]	cullin 3	K03869	0	0	0	1	0
Genetic Information Processing	Folding, Sorting and Degradation	04120 Ubiquitin mediated proteolysis [PATH:ko04120]	ubiquitin-conjugating enzyme E2 D/E [EC:6.3.2.19]	K06689	0	0	2	0	0
Genetic Information Processing	Folding, Sorting and Degradation	04120 Ubiquitin mediated proteolysis [PATH:ko04120]	ubiquitin-like 1-activating enzyme E1 B [EC:6.3.2.19]	K10685	1	0	0	0	0
Genetic Information Processing	Folding, Sorting and Degradation	04122 Sulfur relay system [PATH:ko04122]	adenylyltransferase and sulfurtransferase	K11996	2	6	0	0	0
Genetic Information Processing	Folding, Sorting and Degradation	04122 Sulfur relay system [PATH:ko04122]	molybdenum cofactor biosynthesis protein	K03639	5	2	0	0	0
Genetic Information Processing	Folding, Sorting and Degradation	04122 Sulfur relay system [PATH:ko04122]	molybdenum cofactor biosynthesis protein C	K03637	0	2	3	1	0
Genetic Information Processing	Folding, Sorting and Degradation	04122 Sulfur relay system [PATH:ko04122]	molybdopterin synthase catalytic subunit [EC:2.-.-.-]	K03635	0	1	0	0	0
Genetic Information Processing	Folding, Sorting and Degradation	04122 Sulfur relay system [PATH:ko04122]	thiosulfate/3-mercaptopyruvate sulfurtransferase [EC:2.8.1.1	K01011	1	0	0	5	4
Genetic Information Processing	Folding, Sorting and Degradation	04122 Sulfur relay system [PATH:ko04122]	tRNA 2-thiouridine synthesizing protein E [EC:2.8.1.-]	K11179	1	0	0	0	0
Genetic Information Processing	Folding, Sorting and Degradation	04122 Sulfur relay system [PATH:ko04122]	tRNA-specific 2-thiouridylase [EC:2.8.1.-]	K00566	1	0	2	3	4
Genetic Information Processing	Folding, Sorting and Degradation	04141 Protein processing in endoplasmic	HSP20 family protein	K13993	0	5	0	0	0

Level 1	Level 2	Level 3	Function	KEGG ID	Sequence Reads				
					Day17	Day24	Day31	Day38	Bulk Soil
		reticulum [PATH:ko04141]							
Genetic Information Processing	Replication and Repair	03030 DNA replication [PATH:ko03030]	DNA primase [EC:2.7.7.-]	K02316	4	0	0	4	3
Genetic Information Processing	Replication and Repair	03030 DNA replication [PATH:ko03030]	DNA replication ATP-dependent helicase Dna2 [EC:3.6.4.12]	K10742	0	0	1	0	0
Genetic Information Processing	Replication and Repair	03030 DNA replication [PATH:ko03030]	ribonuclease HI [EC:3.1.26.4]	K03469	0	0	0	0	1
Genetic Information Processing	Replication and Repair	03030 DNA replication [PATH:ko03030]	ribonuclease HII [EC:3.1.26.4]	K03470	0	0	0	1	0
Genetic Information Processing	Replication and Repair	03410 Base excision repair [PATH:ko03410]	A/G-specific adenine glycosylase [EC:3.2.2.-]	K03575	0	1	2	0	0
Genetic Information Processing	Replication and Repair	03410 Base excision repair [PATH:ko03410]	AraC family transcriptional regulator, regulatory protein of	K13529	0	0	0	1	2
Genetic Information Processing	Replication and Repair	03410 Base excision repair [PATH:ko03410]	DNA-3-methyladenine glycosylase I [EC:3.2.2.20]	K01246	0	1	2	3	0
Genetic Information Processing	Replication and Repair	03410 Base excision repair [PATH:ko03410]	DNA-3-methyladenine glycosylase II [EC:3.2.2.21]	K01247	0	0	1	0	0
Genetic Information Processing	Replication and Repair	03410 Base excision repair [PATH:ko03410]	endonuclease III [EC:4.2.99.18]	K10773	1	2	0	0	0
Genetic Information Processing	Replication and Repair	03410 Base excision repair [PATH:ko03410]	endonuclease VIII [EC:3.2.2.- 4.2.99.18]	K05522	0	0	0	0	2
Genetic Information Processing	Replication and Repair	03410 Base excision repair [PATH:ko03410]	exodeoxyribonuclease III [EC:3.1.11.2]	K01142	2	0	0	2	0
Genetic Information Processing	Replication and Repair	03420 Nucleotide excision repair [PATH:ko03420]	DNA excision repair protein ERCC-3 [EC:3.6.4.12]	K10843	0	0	1	0	2

Level 1	Level 2	Level 3	Function	KEGG ID	Sequence Reads				
					Day17	Day24	Day31	Day38	Bulk Soil
Genetic Information Processing	Replication and Repair	03420 Nucleotide excision repair [PATH:ko03420]	excinuclease ABC subunit A	K03701	4	11	6	19	13
Genetic Information Processing	Replication and Repair	03420 Nucleotide excision repair [PATH:ko03420]	excinuclease ABC subunit B	K03702	1	0	4	8	6
Genetic Information Processing	Replication and Repair	03420 Nucleotide excision repair [PATH:ko03420]	excinuclease ABC subunit C	K03703	0	0	2	0	0
Genetic Information Processing	Replication and Repair	03430 Mismatch repair [PATH:ko03430]	DNA helicase II / ATP-dependent DNA helicase PcrA	K03657	0	7	9	11	7
Genetic Information Processing	Replication and Repair	03430 Mismatch repair [PATH:ko03430]	DNA ligase (NAD+) [EC:6.5.1.2]	K01972	0	0	3	0	0
Genetic Information Processing	Replication and Repair	03430 Mismatch repair [PATH:ko03430]	DNA mismatch repair protein MutL	K03572	0	4	2	0	0
Genetic Information Processing	Replication and Repair	03430 Mismatch repair [PATH:ko03430]	DNA mismatch repair protein MutS2	K07456	0	0	1	0	0
Genetic Information Processing	Replication and Repair	03430 Mismatch repair [PATH:ko03430]	exodeoxyribonuclease VII large subunit [EC:3.1.11.6]	K03601	0	1	0	0	0
Genetic Information Processing	Replication and Repair	03430 Mismatch repair [PATH:ko03430]	single-stranded-DNA-specific exonuclease [EC:3.1.-.-]	K07462	0	0	0	2	0
Genetic Information Processing	Replication and Repair	03440 Homologous recombination [PATH:ko03440]	ATP-dependent DNA helicase RecG [EC:3.6.4.12]	K03655	0	3	3	0	0
Genetic Information Processing	Replication and Repair	03440 Homologous recombination [PATH:ko03440]	crossover junction endodeoxyribonuclease RuvC [EC:3.1.22.4]	K01159	0	0	0	1	0
Genetic Information Processing	Replication and Repair	03440 Homologous recombination [PATH:ko03440]	DNA polymerase delta subunit 1 [EC:2.7.7.7]	K02327	0	0	0	1	0
Genetic Information Processing	Replication and Repair	03440 Homologous recombination [PATH:ko03440]	DNA polymerase III subunit alpha [EC:2.7.7.7]	K02337	4	0	0	4	0

Level 1	Level 2	Level 3	Function	KEGG ID	Sequence Reads				
					Day17	Day24	Day31	Day38	Bulk Soil
Genetic Information Processing	Replication and Repair	03440 Homologous recombination [PATH:ko03440]	DNA polymerase III subunit beta [EC:2.7.7.7]	K02338	2	3	2	0	5
Genetic Information Processing	Replication and Repair	03440 Homologous recombination [PATH:ko03440]	DNA polymerase III subunit delta [EC:2.7.7.7]	K02340	0	0	0	0	4
Genetic Information Processing	Replication and Repair	03440 Homologous recombination [PATH:ko03440]	DNA polymerase III subunit delta' [EC:2.7.7.7]	K02341	1	0	0	0	0
Genetic Information Processing	Replication and Repair	03440 Homologous recombination [PATH:ko03440]	DNA polymerase III subunit epsilon [EC:2.7.7.7]	K02342	4	1	0	0	2
Genetic Information Processing	Replication and Repair	03440 Homologous recombination [PATH:ko03440]	DNA polymerase III subunit gamma/tau [EC:2.7.7.7]	K02343	2	3	4	0	5
Genetic Information Processing	Replication and Repair	03440 Homologous recombination [PATH:ko03440]	DNA replication and repair protein RecF	K03629	0	0	0	2	0
Genetic Information Processing	Replication and Repair	03440 Homologous recombination [PATH:ko03440]	exodeoxyribonuclease V alpha subunit [EC:3.1.11.5]	K03581	1	2	0	0	0
Genetic Information Processing	Replication and Repair	03440 Homologous recombination [PATH:ko03440]	exodeoxyribonuclease V beta subunit [EC:3.1.11.5]	K03582	0	3	0	0	0
Genetic Information Processing	Replication and Repair	03440 Homologous recombination [PATH:ko03440]	exodeoxyribonuclease V gamma subunit [EC:3.1.11.5]	K03583	1	0	0	0	0
Genetic Information Processing	Replication and Repair	03440 Homologous recombination [PATH:ko03440]	holliday junction DNA helicase RuvB	K03551	0	2	3	0	0
Genetic Information Processing	Replication and Repair	03440 Homologous recombination [PATH:ko03440]	primosomal protein N' (replication factor Y) (superfamily II	K04066	1	0	0	0	0
Genetic Information Processing	Replication and Repair	03440 Homologous recombination [PATH:ko03440]	primosomal replication protein N''	K04067	0	0	0	0	1
Genetic Information Processing	Replication and Repair	03440 Homologous recombination [PATH:ko03440]	recombination protein RecA	K03553	5	5	3	3	3

Level 1	Level 2	Level 3	Function	KEGG ID	Sequence Reads				
					Day17	Day24	Day31	Day38	Bulk Soil
Genetic Information Processing	Replication and Repair	03440 Homologous recombination [PATH:ko03440]	single-strand DNA-binding protein	K03111	0	0	0	0	1
Genetic Information Processing	Replication and Repair	03450 Non-homologous end-joining [PATH:ko03450]	DNA ligase (ATP) [EC:6.5.1.1]	K01971	9	14	0	13	0
Genetic Information Processing	Transcription	03020 RNA polymerase [PATH:ko03020]	DNA-directed RNA polymerase I subunit A2 [EC:2.7.7.6]	K03002	0	0	1	0	0
Genetic Information Processing	Transcription	03020 RNA polymerase [PATH:ko03020]	DNA-directed RNA polymerase subunit beta [EC:2.7.7.6]	K03043	35	6	16	26	14
Genetic Information Processing	Transcription	03020 RNA polymerase [PATH:ko03020]	DNA-directed RNA polymerase subunit beta' [EC:2.7.7.6]	K03046	34	16	13	21	13
Genetic Information Processing	Transcription	03020 RNA polymerase [PATH:ko03020]	RNA polymerase primary sigma factor	K03086	0	6	8	13	8
Genetic Information Processing	Transcription	03020 RNA polymerase [PATH:ko03020]	RNA polymerase sigma-32 factor	K03089	0	0	0	4	0
Genetic Information Processing	Transcription	03020 RNA polymerase [PATH:ko03020]	RNA polymerase sigma-70 factor, ECF subfamily	K03088	0	0	29	0	0
Genetic Information Processing	Transcription	03020 RNA polymerase [PATH:ko03020]	RNA polymerase sporulation-specific sigma factor	K03091	1	0	0	0	0
Genetic Information Processing	Transcription	03040 Spliceosome [PATH:ko03040]	pre-mRNA-processing factor 8	K12856	7	0	0	0	0
Genetic Information Processing	Transcription	03040 Spliceosome [PATH:ko03040]	pre-mRNA-splicing helicase BRR2 [EC:3.6.4.13]	K12854	2	0	0	0	0
Genetic Information Processing	Transcription	03040 Spliceosome [PATH:ko03040]	splicing factor 3B subunit 1	K12828	1	0	0	0	0

Level 1	Level 2	Level 3	Function	KEGG ID	Sequence Reads				
					Day17	Day24	Day31	Day38	Bulk Soil
Genetic Information Processing	Transcription	03040 Spliceosome [PATH:ko03040]	splicing factor U2AF 65 kDa subunit	K12837	1	0	0	0	0
Genetic Information Processing	Translation	00970 Aminoacyl-tRNA biosynthesis [PATH:ko00970]	alanyl-tRNA synthetase [EC:6.1.1.7]	K01872	0	5	0	4	6
Genetic Information Processing	Translation	00970 Aminoacyl-tRNA biosynthesis [PATH:ko00970]	arginyl-tRNA synthetase [EC:6.1.1.19]	K01887	1	7	5	0	0
Genetic Information Processing	Translation	00970 Aminoacyl-tRNA biosynthesis [PATH:ko00970]	asparaginyl-tRNA synthetase [EC:6.1.1.22]	K01893	0	1	4	0	0
Genetic Information Processing	Translation	00970 Aminoacyl-tRNA biosynthesis [PATH:ko00970]	aspartyl-tRNA synthetase [EC:6.1.1.12]	K01876	6	7	5	0	0
Genetic Information Processing	Translation	00970 Aminoacyl-tRNA biosynthesis [PATH:ko00970]	aspartyl-tRNA(Asn)/glutamyl-tRNA (Gln) amidotransferase	K02433	7	0	0	0	0
Genetic Information Processing	Translation	00970 Aminoacyl-tRNA biosynthesis [PATH:ko00970]	aspartyl-tRNA(Asn)/glutamyl-tRNA (Gln) amidotransferase	K02434	0	2	0	0	0
Genetic Information Processing	Translation	00970 Aminoacyl-tRNA biosynthesis [PATH:ko00970]	cysteinyl-tRNA synthetase [EC:6.1.1.16]	K01883	0	0	0	3	0
Genetic Information Processing	Translation	00970 Aminoacyl-tRNA biosynthesis [PATH:ko00970]	glutaminyl-tRNA synthetase [EC:6.1.1.18]	K01886	3	2	2	0	0
Genetic Information Processing	Translation	00970 Aminoacyl-tRNA biosynthesis [PATH:ko00970]	glutamyl-tRNA synthetase [EC:6.1.1.17]	K01885	0	4	0	0	8
Genetic Information Processing	Translation	00970 Aminoacyl-tRNA biosynthesis [PATH:ko00970]	glycyl-tRNA synthetase [EC:6.1.1.14]	K01880	0	3	1	5	0
Genetic Information Processing	Translation	00970 Aminoacyl-tRNA biosynthesis [PATH:ko00970]	glycyl-tRNA synthetase alpha chain [EC:6.1.1.14]	K01878	0	2	2	2	1
Genetic Information Processing	Translation	00970 Aminoacyl-tRNA biosynthesis [PATH:ko00970]	glycyl-tRNA synthetase beta chain [EC:6.1.1.14]	K01879	0	0	0	6	3

Level 1	Level 2	Level 3	Function	KEGG ID	Sequence Reads				
					Day17	Day24	Day31	Day38	Bulk Soil
Genetic Information Processing	Translation	00970 Aminoacyl-tRNA biosynthesis [PATH:ko00970]	histidyl-tRNA synthetase [EC:6.1.1.21]	K01892	1	0	0	0	0
Genetic Information Processing	Translation	00970 Aminoacyl-tRNA biosynthesis [PATH:ko00970]	isoleucyl-tRNA synthetase [EC:6.1.1.5]	K01870	0	7	0	9	0
Genetic Information Processing	Translation	00970 Aminoacyl-tRNA biosynthesis [PATH:ko00970]	leucyl-tRNA synthetase [EC:6.1.1.4]	K01869	0	2	3	8	7
Genetic Information Processing	Translation	00970 Aminoacyl-tRNA biosynthesis [PATH:ko00970]	L-seryl-tRNA(Ser) seleniumtransferase [EC:2.9.1.1]	K01042	0	0	0	3	2
Genetic Information Processing	Translation	00970 Aminoacyl-tRNA biosynthesis [PATH:ko00970]	lysyl-tRNA synthetase, class I [EC:6.1.1.6]	K04566	1	1	1	0	0
Genetic Information Processing	Translation	00970 Aminoacyl-tRNA biosynthesis [PATH:ko00970]	lysyl-tRNA synthetase, class II [EC:6.1.1.6]	K04567	2	0	0	0	0
Genetic Information Processing	Translation	00970 Aminoacyl-tRNA biosynthesis [PATH:ko00970]	lysyl-tRNA synthetase, class II [EC:6.1.1.6]	K04568	0	0	0	1	1
Genetic Information Processing	Translation	00970 Aminoacyl-tRNA biosynthesis [PATH:ko00970]	methionyl-tRNA formyltransferase [EC:2.1.2.9]	K00604	0	0	0	0	3
Genetic Information Processing	Translation	00970 Aminoacyl-tRNA biosynthesis [PATH:ko00970]	nondiscriminating aspartyl-tRNA synthetase [EC:6.1.1.23]	K09759	0	1	0	0	0
Genetic Information Processing	Translation	00970 Aminoacyl-tRNA biosynthesis [PATH:ko00970]	phenylalanyl-tRNA synthetase alpha chain [EC:6.1.1.20]	K01889	6	2	0	3	5
Genetic Information Processing	Translation	00970 Aminoacyl-tRNA biosynthesis [PATH:ko00970]	phenylalanyl-tRNA synthetase beta chain [EC:6.1.1.20]	K01890	2	0	0	2	0
Genetic Information Processing	Translation	00970 Aminoacyl-tRNA biosynthesis [PATH:ko00970]	prolyl-tRNA synthetase [EC:6.1.1.15]	K01881	0	0	2	0	3
Genetic Information Processing	Translation	00970 Aminoacyl-tRNA biosynthesis [PATH:ko00970]	seryl-tRNA synthetase [EC:6.1.1.11]	K01875	6	0	0	1	0

Level 1	Level 2	Level 3	Function	KEGG ID	Sequence Reads				
					Day17	Day24	Day31	Day38	Bulk Soil
Genetic Information Processing	Translation	00970 Aminoacyl-tRNA biosynthesis [PATH:ko00970]	threonyl-tRNA synthetase [EC:6.1.1.3]	K01868	4	3	7	2	0
Genetic Information Processing	Translation	00970 Aminoacyl-tRNA biosynthesis [PATH:ko00970]	tryptophanyl-tRNA synthetase [EC:6.1.1.2]	K01867	0	2	3	4	0
Genetic Information Processing	Translation	00970 Aminoacyl-tRNA biosynthesis [PATH:ko00970]	tyrosyl-tRNA synthetase [EC:6.1.1.1]	K01866	1	0	2	4	5
Genetic Information Processing	Translation	00970 Aminoacyl-tRNA biosynthesis [PATH:ko00970]	valyl-tRNA synthetase [EC:6.1.1.9]	K01873	0	6	6	0	0
Genetic Information Processing	Translation	03010 Ribosome [PATH:ko03010]	large subunit ribosomal protein L1	K02863	7	0	5	4	0
Genetic Information Processing	Translation	03010 Ribosome [PATH:ko03010]	large subunit ribosomal protein L10	K02864	15	0	2	13	3
Genetic Information Processing	Translation	03010 Ribosome [PATH:ko03010]	large subunit ribosomal protein L10e	K02866	0	0	1	1	0
Genetic Information Processing	Translation	03010 Ribosome [PATH:ko03010]	large subunit ribosomal protein L11	K02867	9	0	3	5	1
Genetic Information Processing	Translation	03010 Ribosome [PATH:ko03010]	large subunit ribosomal protein L11e	K02868	0	0	0	1	0
Genetic Information Processing	Translation	03010 Ribosome [PATH:ko03010]	large subunit ribosomal protein L12e	K02870	1	0	0	0	0
Genetic Information Processing	Translation	03010 Ribosome [PATH:ko03010]	large subunit ribosomal protein L13	K02871	8	3	5	3	0
Genetic Information Processing	Translation	03010 Ribosome [PATH:ko03010]	large subunit ribosomal protein L14	K02874	7	3	2	5	1
Genetic Information Processing	Translation	03010 Ribosome [PATH:ko03010]	large subunit ribosomal protein L15	K02876	4	0	2	0	0

Level 1	Level 2	Level 3	Function	KEGG ID	Sequence Reads				
					Day17	Day24	Day31	Day38	Bulk Soil
Genetic Information Processing	Translation	03010 Ribosome [PATH:ko03010]	large subunit ribosomal protein L15e	K02877	0	0	1	0	0
Genetic Information Processing	Translation	03010 Ribosome [PATH:ko03010]	large subunit ribosomal protein L16	K02878	6	3	8	3	2
Genetic Information Processing	Translation	03010 Ribosome [PATH:ko03010]	large subunit ribosomal protein L17	K02879	6	1	4	3	0
Genetic Information Processing	Translation	03010 Ribosome [PATH:ko03010]	large subunit ribosomal protein L18	K02881	0	1	2	0	3
Genetic Information Processing	Translation	03010 Ribosome [PATH:ko03010]	large subunit ribosomal protein L18e	K02883	1	0	0	0	0
Genetic Information Processing	Translation	03010 Ribosome [PATH:ko03010]	large subunit ribosomal protein L19	K02884	5	1	1	2	2
Genetic Information Processing	Translation	03010 Ribosome [PATH:ko03010]	large subunit ribosomal protein L19e	K02885	1	0	0	0	0
Genetic Information Processing	Translation	03010 Ribosome [PATH:ko03010]	large subunit ribosomal protein L2	K02886	6	4	1	9	4
Genetic Information Processing	Translation	03010 Ribosome [PATH:ko03010]	large subunit ribosomal protein L20	K02887	5	1	5	6	4
Genetic Information Processing	Translation	03010 Ribosome [PATH:ko03010]	large subunit ribosomal protein L21	K02888	8	0	1	0	0
Genetic Information Processing	Translation	03010 Ribosome [PATH:ko03010]	large subunit ribosomal protein L22	K02890	0	0	2	1	0
Genetic Information Processing	Translation	03010 Ribosome [PATH:ko03010]	large subunit ribosomal protein L23	K02892	0	1	0	0	0
Genetic Information Processing	Translation	03010 Ribosome [PATH:ko03010]	large subunit ribosomal protein L23Ae	K02893	0	0	1	0	0

Level 1	Level 2	Level 3	Function	KEGG ID	Sequence Reads				
					Day17	Day24	Day31	Day38	Bulk Soil
Genetic Information Processing	Translation	03010 Ribosome [PATH:ko03010]	large subunit ribosomal protein L23e	K02894	0	0	0	1	1
Genetic Information Processing	Translation	03010 Ribosome [PATH:ko03010]	large subunit ribosomal protein L24	K02895	0	0	4	3	0
Genetic Information Processing	Translation	03010 Ribosome [PATH:ko03010]	large subunit ribosomal protein L25	K02897	0	0	0	2	0
Genetic Information Processing	Translation	03010 Ribosome [PATH:ko03010]	large subunit ribosomal protein L26e	K02898	0	0	1	1	0
Genetic Information Processing	Translation	03010 Ribosome [PATH:ko03010]	large subunit ribosomal protein L27	K02899	7	2	0	2	2
Genetic Information Processing	Translation	03010 Ribosome [PATH:ko03010]	large subunit ribosomal protein L28	K02902	0	3	4	2	0
Genetic Information Processing	Translation	03010 Ribosome [PATH:ko03010]	large subunit ribosomal protein L28e	K02903	0	0	0	1	0
Genetic Information Processing	Translation	03010 Ribosome [PATH:ko03010]	large subunit ribosomal protein L29	K02904	3	0	3	0	0
Genetic Information Processing	Translation	03010 Ribosome [PATH:ko03010]	large subunit ribosomal protein L3	K02906	13	3	0	9	0
Genetic Information Processing	Translation	03010 Ribosome [PATH:ko03010]	large subunit ribosomal protein L30	K02907	1	0	0	0	0
Genetic Information Processing	Translation	03010 Ribosome [PATH:ko03010]	large subunit ribosomal protein L30e	K02908	1	1	0	0	0
Genetic Information Processing	Translation	03010 Ribosome [PATH:ko03010]	large subunit ribosomal protein L31	K02909	2	0	0	2	0
Genetic Information Processing	Translation	03010 Ribosome [PATH:ko03010]	large subunit ribosomal protein L32	K02911	1	2	2	0	0

Level 1	Level 2	Level 3	Function	KEGG ID	Sequence Reads				
					Day17	Day24	Day31	Day38	Bulk Soil
Genetic Information Processing	Translation	03010 Ribosome [PATH:ko03010]	large subunit ribosomal protein L33	K02913	2	1	0	3	0
Genetic Information Processing	Translation	03010 Ribosome [PATH:ko03010]	large subunit ribosomal protein L34	K02914	1	2	0	2	1
Genetic Information Processing	Translation	03010 Ribosome [PATH:ko03010]	large subunit ribosomal protein L35	K02916	2	0	2	3	0
Genetic Information Processing	Translation	03010 Ribosome [PATH:ko03010]	large subunit ribosomal protein L35e	K02918	0	0	0	1	0
Genetic Information Processing	Translation	03010 Ribosome [PATH:ko03010]	large subunit ribosomal protein L36	K02919	0	2	1	1	0
Genetic Information Processing	Translation	03010 Ribosome [PATH:ko03010]	large subunit ribosomal protein L39e	K02924	0	1	0	0	0
Genetic Information Processing	Translation	03010 Ribosome [PATH:ko03010]	large subunit ribosomal protein L4	K02926	0	3	4	12	2
Genetic Information Processing	Translation	03010 Ribosome [PATH:ko03010]	large subunit ribosomal protein L40e	K02927	0	0	2	0	0
Genetic Information Processing	Translation	03010 Ribosome [PATH:ko03010]	large subunit ribosomal protein L4e	K02930	5	0	0	0	0
Genetic Information Processing	Translation	03010 Ribosome [PATH:ko03010]	large subunit ribosomal protein L5	K02931	10	2	4	10	0
Genetic Information Processing	Translation	03010 Ribosome [PATH:ko03010]	large subunit ribosomal protein L6	K02933	0	2	0	0	1
Genetic Information Processing	Translation	03010 Ribosome [PATH:ko03010]	large subunit ribosomal protein L7/L12	K02935	8	3	1	6	1
Genetic Information Processing	Translation	03010 Ribosome [PATH:ko03010]	large subunit ribosomal protein L9	K02939	6	0	0	0	3

Level 1	Level 2	Level 3	Function	KEGG ID	Sequence Reads				
					Day17	Day24	Day31	Day38	Bulk Soil
Genetic Information Processing	Translation	03010 Ribosome [PATH:ko03010]	large subunit ribosomal protein L9e	K02940	1	0	0	0	0
Genetic Information Processing	Translation	03010 Ribosome [PATH:ko03010]	small subunit ribosomal protein S10	K02946	2	4	3	5	2
Genetic Information Processing	Translation	03010 Ribosome [PATH:ko03010]	small subunit ribosomal protein S11	K02948	5	2	2	4	1
Genetic Information Processing	Translation	03010 Ribosome [PATH:ko03010]	small subunit ribosomal protein S11e	K02949	0	0	1	0	0
Genetic Information Processing	Translation	03010 Ribosome [PATH:ko03010]	small subunit ribosomal protein S12	K02950	4	1	3	2	0
Genetic Information Processing	Translation	03010 Ribosome [PATH:ko03010]	small subunit ribosomal protein S12e	K02951	0	1	1	0	0
Genetic Information Processing	Translation	03010 Ribosome [PATH:ko03010]	small subunit ribosomal protein S13	K02952	5	3	1	6	2
Genetic Information Processing	Translation	03010 Ribosome [PATH:ko03010]	small subunit ribosomal protein S14	K02954	3	0	0	2	0
Genetic Information Processing	Translation	03010 Ribosome [PATH:ko03010]	small subunit ribosomal protein S15	K02956	5	3	1	0	0
Genetic Information Processing	Translation	03010 Ribosome [PATH:ko03010]	small subunit ribosomal protein S15Ae	K02957	1	1	0	0	0
Genetic Information Processing	Translation	03010 Ribosome [PATH:ko03010]	small subunit ribosomal protein S15e	K02958	0	0	1	0	0
Genetic Information Processing	Translation	03010 Ribosome [PATH:ko03010]	small subunit ribosomal protein S16e	K02960	0	1	0	0	0
Genetic Information Processing	Translation	03010 Ribosome [PATH:ko03010]	small subunit ribosomal protein S17	K02961	4	0	0	0	0

Level 1	Level 2	Level 3	Function	KEGG ID	Sequence Reads				
					Day17	Day24	Day31	Day38	Bulk Soil
Genetic Information Processing	Translation	03010 Ribosome [PATH:ko03010]	small subunit ribosomal protein S18	K02963	3	0	0	3	2
Genetic Information Processing	Translation	03010 Ribosome [PATH:ko03010]	small subunit ribosomal protein S18e	K02964	0	0	0	1	0
Genetic Information Processing	Translation	03010 Ribosome [PATH:ko03010]	small subunit ribosomal protein S19	K02965	3	4	1	3	1
Genetic Information Processing	Translation	03010 Ribosome [PATH:ko03010]	small subunit ribosomal protein S2	K02967	8	0	1	6	0
Genetic Information Processing	Translation	03010 Ribosome [PATH:ko03010]	small subunit ribosomal protein S20	K02968	9	2	0	0	1
Genetic Information Processing	Translation	03010 Ribosome [PATH:ko03010]	small subunit ribosomal protein S21	K02970	3	1	3	2	2
Genetic Information Processing	Translation	03010 Ribosome [PATH:ko03010]	small subunit ribosomal protein S23e	K02973	0	0	2	0	0
Genetic Information Processing	Translation	03010 Ribosome [PATH:ko03010]	small subunit ribosomal protein S27Ae	K02977	0	0	3	0	0
Genetic Information Processing	Translation	03010 Ribosome [PATH:ko03010]	small subunit ribosomal protein S27e	K02978	0	0	0	1	0
Genetic Information Processing	Translation	03010 Ribosome [PATH:ko03010]	small subunit ribosomal protein S2e	K02981	0	0	1	0	0
Genetic Information Processing	Translation	03010 Ribosome [PATH:ko03010]	small subunit ribosomal protein S3	K02982	10	3	8	3	3
Genetic Information Processing	Translation	03010 Ribosome [PATH:ko03010]	small subunit ribosomal protein S3e	K02985	1	0	1	1	0
Genetic Information Processing	Translation	03010 Ribosome [PATH:ko03010]	small subunit ribosomal protein S4	K02986	6	2	2	5	0

Level 1	Level 2	Level 3	Function	KEGG ID	Sequence Reads				
					Day17	Day24	Day31	Day38	Bulk Soil
Genetic Information Processing	Translation	03010 Ribosome [PATH:ko03010]	small subunit ribosomal protein S4e	K02987	0	0	0	1	0
Genetic Information Processing	Translation	03010 Ribosome [PATH:ko03010]	small subunit ribosomal protein S5	K02988	6	0	3	3	4
Genetic Information Processing	Translation	03010 Ribosome [PATH:ko03010]	small subunit ribosomal protein S5e	K02989	4	0	2	0	0
Genetic Information Processing	Translation	03010 Ribosome [PATH:ko03010]	small subunit ribosomal protein S6	K02990	4	0	0	2	1
Genetic Information Processing	Translation	03010 Ribosome [PATH:ko03010]	small subunit ribosomal protein S7	K02992	0	1	2	5	0
Genetic Information Processing	Translation	03010 Ribosome [PATH:ko03010]	small subunit ribosomal protein S7e	K02993	1	0	0	0	0
Genetic Information Processing	Translation	03010 Ribosome [PATH:ko03010]	small subunit ribosomal protein S8	K02994	9	1	0	6	1
Genetic Information Processing	Translation	03010 Ribosome [PATH:ko03010]	small subunit ribosomal protein S9	K02996	6	1	2	0	4
Genetic Information Processing	Translation	03010 Ribosome [PATH:ko03010]	small subunit ribosomal protein SAe	K02998	1	0	1	0	0
Genetic Information Processing	Translation	03013 RNA transport [PATH:ko03013]	elongation factor EF-1 alpha subunit [EC:3.6.5.3]	K03231	4	2	2	1	0
Genetic Information Processing	Translation	03013 RNA transport [PATH:ko03013]	GTP-binding nuclear protein Ran	K07936	1	0	0	0	0
Genetic Information Processing	Translation	03013 RNA transport [PATH:ko03013]	nuclear pore complex protein Nup98-Nup96	K14297	1	0	0	0	0
Genetic Information Processing	Translation	03013 RNA transport [PATH:ko03013]	ribonuclease P subunit RPR2 [EC:3.1.26.5]	K03540	0	0	0	0	1

Level 1	Level 2	Level 3	Function	KEGG ID	Sequence Reads				
					Day17	Day24	Day31	Day38	Bulk Soil
Genetic Information Processing	Translation	03015 mRNA surveillance pathway [PATH:ko03015]	poly(A) polymerase [EC:2.7.7.19]	K14376	1	0	1	0	0
Human Diseases	Cancers	05211 Renal cell carcinoma [PATH:ko05211]	fumarate hydratase, class II [EC:4.2.1.2]	K01679	2	5	4	2	6
Human Diseases	Cancers	05211 Renal cell carcinoma [PATH:ko05211]	Ras-related protein Rap-1A	K04353	1	0	1	0	0
Human Diseases	Cancers	05215 Prostate cancer [PATH:ko05215]	heat shock protein 90kDa beta	K09487	1	0	0	0	0
Human Diseases	Cancers	05215 Prostate cancer [PATH:ko05215]	molecular chaperone HtpG	K04079	5	2	6	5	4
Human Diseases	Cancers	05219 Bladder cancer [PATH:ko05219]	thymidine phosphorylase [EC:2.4.2.4]	K00758	0	1	0	0	0
Human Diseases	Cardiovascular Diseases	05416 Viral myocarditis [PATH:ko05416]	myosin heavy chain	K10352	0	0	3	0	0
Human Diseases	Immune System Diseases	05322 Systemic lupus erythematosus [PATH:ko05322]	histone H2A	K11251	1	1	2	0	0
Human Diseases	Immune System Diseases	05322 Systemic lupus erythematosus [PATH:ko05322]	histone H2B	K11252	0	0	2	0	0
Human Diseases	Immune System Diseases	05322 Systemic lupus erythematosus [PATH:ko05322]	histone H3	K11253	0	0	0	1	0
Human Diseases	Immune System Diseases	05322 Systemic lupus erythematosus [PATH:ko05322]	histone H4	K11254	0	2	2	0	0
Human Diseases	Infectious Diseases	05100 Bacterial invasion of epithelial cells [PATH:ko05100]	actin beta/gamma 1	K05692	1	0	4	2	0

Level 1	Level 2	Level 3	Function	KEGG ID	Sequence Reads				
					Day17	Day24	Day31	Day38	Bulk Soil
Human Diseases	Infectious Diseases	05100 Bacterial invasion of epithelial cells [PATH:ko05100]	dynamin GTPase [EC:3.6.5.5]	K01528	0	1	0	0	0
Human Diseases	Infectious Diseases	05100 Bacterial invasion of epithelial cells [PATH:ko05100]	Ras-related C3 botulinum toxin substrate 1	K04392	0	0	0	1	0
Human Diseases	Infectious Diseases	05111 Vibrio cholerae pathogenic cycle [PATH:ko05111]	RNA polymerase nonessential primary-like sigma factor	K03087	2	0	6	4	1
Human Diseases	Infectious Diseases	05111 Vibrio cholerae pathogenic cycle [PATH:ko05111]	RNA polymerase sigma factor for flagellar operon FliA	K02405	0	0	1	2	0
Human Diseases	Infectious Diseases	05111 Vibrio cholerae pathogenic cycle [PATH:ko05111]	RNA polymerase sigma-54 factor	K03092	1	0	0	0	0
Human Diseases	Infectious Diseases	05120 Epithelial cell signaling in Helicobacter pylori infection [PATH:ko05120]	urease subunit alpha [EC:3.5.1.5]	K01428	1	3	2	0	2
Human Diseases	Infectious Diseases	05120 Epithelial cell signaling in Helicobacter pylori infection [PATH:ko05120]	V-type H+-transporting ATPase 16kDa proteolipid subunit	K02155	0	1	0	2	0
Human Diseases	Infectious Diseases	05130 Pathogenic Escherichia coli infection [PATH:ko05130]	tubulin alpha	K07374	3	0	1	1	0
Human Diseases	Infectious Diseases	05130 Pathogenic Escherichia coli infection [PATH:ko05130]	tubulin beta	K07375	4	1	2	0	0

Level 1	Level 2	Level 3	Function	KEGG ID	Sequence Reads				
					Day17	Day24	Day31	Day38	Bulk Soil
Human Diseases	Infectious Diseases	05142 Chagas disease (American trypanosomiasis) [PATH:ko05142]	peptidyl-dipeptidase A [EC:3.4.15.1]	K01283	1	0	0	0	0
Human Diseases	Infectious Diseases	05143 African trypanosomiasis [PATH:ko05143]	oligopeptidase B [EC:3.4.21.83]	K01354	0	1	0	3	2
Human Diseases	Infectious Diseases	05143 African trypanosomiasis [PATH:ko05143]	thimet oligopeptidase [EC:3.4.24.15]	K01392	0	1	1	0	0
Human Diseases	Infectious Diseases	05144 Malaria [PATH:ko05144]	thrombospondin	K04659	0	0	1	0	0
Human Diseases	Infectious Diseases	05145 Toxoplasmosis [PATH:ko05145]	heat shock 70kDa protein 1/8	K03283	2	0	3	0	0
Human Diseases	Infectious Diseases	05146 Amoebiasis [PATH:ko05146]	actinin alpha	K05699	2	0	0	0	0
Human Diseases	Infectious Diseases	05146 Amoebiasis [PATH:ko05146]	PTK2 protein tyrosine kinase 2 [EC:2.7.10.2]	K05725	1	0	0	0	0
Human Diseases	Infectious Diseases	05146 Amoebiasis [PATH:ko05146]	Ras-related protein Rab-7A	K07897	1	1	0	0	0
Human Diseases	Infectious Diseases	05160 Hepatitis C [PATH:ko05160]	glycogen synthase kinase 3 beta [EC:2.7.11.26]	K03083	0	0	1	0	0
Human Diseases	Metabolic Diseases	04930 Type II diabetes mellitus [PATH:ko04930]	pyruvate kinase [EC:2.7.1.40]	K00873	5	0	0	0	0
Human Diseases	Metabolic Diseases	04940 Type I diabetes mellitus [PATH:ko04940]	chaperonin GroEL	K04077	72	12	20	29	13
Human Diseases	Metabolic Diseases	04940 Type I diabetes mellitus [PATH:ko04940]	glutamate decarboxylase [EC:4.1.1.15]	K01580	1	0	0	0	0
Human Diseases	Neurodegenerative Diseases	05010 Alzheimer's disease [PATH:ko05010]	calmodulin	K02183	0	0	1	0	0
Human Diseases	Neurodegenerative Diseases	05010 Alzheimer's disease [PATH:ko05010]	glyceraldehyde 3-phosphate dehydrogenase [EC:1.2.1.12]	K00134	11	0	5	0	4

Level 1	Level 2	Level 3	Function	KEGG ID	Sequence Reads				
					Day17	Day24	Day31	Day38	Bulk Soil
Human Diseases	Neurodegenerative Diseases	05012 Parkinson's disease [PATH:ko05012]	NADH dehydrogenase I subunit 1 [EC:1.6.5.3]	K03878	2	0	0	0	0
Human Diseases	Neurodegenerative Diseases	05012 Parkinson's disease [PATH:ko05012]	NADH dehydrogenase I subunit 2 [EC:1.6.5.3]	K03879	1	0	0	0	0
Human Diseases	Neurodegenerative Diseases	05012 Parkinson's disease [PATH:ko05012]	NADH dehydrogenase I subunit 4 [EC:1.6.5.3]	K03881	0	0	1	0	0
Human Diseases	Neurodegenerative Diseases	05012 Parkinson's disease [PATH:ko05012]	NADH dehydrogenase I subunit 5 [EC:1.6.5.3]	K03883	1	0	0	0	0
Human Diseases	Neurodegenerative Diseases	05012 Parkinson's disease [PATH:ko05012]	NADH dehydrogenase I subunit 6 [EC:1.6.5.3]	K03884	0	1	0	0	0
Human Diseases	Neurodegenerative Diseases	05014 Amyotrophic lateral sclerosis (ALS) [PATH:ko05014]	catalase [EC:1.11.1.6]	K03781	3	3	2	1	2
Human Diseases	Neurodegenerative Diseases	05014 Amyotrophic lateral sclerosis (ALS) [PATH:ko05014]	protein phosphatase 3, catalytic subunit [EC:3.1.3.16]	K04348	0	0	0	1	0
Human Diseases	Neurodegenerative Diseases	05016 Huntington's disease [PATH:ko05016]	cytochrome c oxidase subunit I [EC:1.9.3.1]	K02256	2	1	0	4	0
Human Diseases	Neurodegenerative Diseases	05016 Huntington's disease [PATH:ko05016]	cytochrome c oxidase subunit II [EC:1.9.3.1]	K02261	1	0	0	0	0
Human Diseases	Neurodegenerative Diseases	05016 Huntington's disease [PATH:ko05016]	cytochrome c oxidase subunit III [EC:1.9.3.1]	K02262	0	1	0	0	0
Human Diseases	Neurodegenerative Diseases	05016 Huntington's disease	DNA-directed RNA polymerase II subunit A [EC:2.7.7.6]	K03006	0	0	2	0	0

Level 1	Level 2	Level 3	Function	KEGG ID	Sequence Reads				
					Day17	Day24	Day31	Day38	Bulk Soil
		[PATH:ko05016]							
Human Diseases	Neurodegenerative Diseases	05016 Huntington's disease [PATH:ko05016]	dynein heavy chain, axonemal	K10408	0	1	0	0	0
Human Diseases	Neurodegenerative Diseases	05016 Huntington's disease [PATH:ko05016]	F-type H+-transporting ATPase subunit a [EC:3.6.3.14]	K02126	1	0	0	0	0
Human Diseases	Neurodegenerative Diseases	05016 Huntington's disease [PATH:ko05016]	F-type H+-transporting ATPase subunit alpha [EC:3.6.3.14]	K02132	0	1	0	3	0
Human Diseases	Neurodegenerative Diseases	05016 Huntington's disease [PATH:ko05016]	F-type H+-transporting ATPase subunit beta [EC:3.6.3.14]	K02133	0	0	0	1	0
Human Diseases	Neurodegenerative Diseases	05016 Huntington's disease [PATH:ko05016]	F-type H+-transporting ATPase subunit c [EC:3.6.3.14]	K02128	0	0	2	0	0
Human Diseases	Neurodegenerative Diseases	05016 Huntington's disease [PATH:ko05016]	NADH dehydrogenase (ubiquinone) Fe-S protein 1 [EC:1.6.5.3	K03934	0	0	0	0	1
Human Diseases	Neurodegenerative Diseases	05016 Huntington's disease [PATH:ko05016]	NADH dehydrogenase (ubiquinone) Fe-S protein 2 [EC:1.6.5.3	K03935	0	1	1	0	0
Human Diseases	Neurodegenerative Diseases	05016 Huntington's disease [PATH:ko05016]	NADH dehydrogenase (ubiquinone) Fe-S protein 3 [EC:1.6.5.3	K03936	0	0	1	0	0
Human Diseases	Neurodegenerative Diseases	05016 Huntington's disease [PATH:ko05016]	NADH dehydrogenase (ubiquinone) flavoprotein 1 [EC:1.6.5.3	K03942	0	0	0	1	0
Human Diseases	Neurodegenerative Diseases	05016 Huntington's disease	solute carrier family 25 (mitochondrial carrier; adenine	K05863	2	0	0	2	0

Level 1	Level 2	Level 3	Function	KEGG ID	Sequence Reads				
					Day17	Day24	Day31	Day38	Bulk Soil
		[PATH:ko05016]							
Human Diseases	Neurodegenerative Diseases	05016 Huntington's disease [PATH:ko05016]	superoxide dismutase, Fe-Mn family [EC:1.15.1.1]	K04564	0	0	0	2	0
Human Diseases	Neurodegenerative Diseases	05016 Huntington's disease [PATH:ko05016]	ubiquinol-cytochrome c reductase cytochrome b subunit	K00412	5	0	0	1	1
Human Diseases	Neurodegenerative Diseases	05016 Huntington's disease [PATH:ko05016]	ubiquinol-cytochrome c reductase iron-sulfur subunit	K00411	0	2	0	0	1
Human Diseases	Neurodegenerative Diseases	05020 Prion diseases [PATH:ko05020]	heat shock 70kDa protein 5	K09490	1	0	2	0	0
Human Diseases	Neurodegenerative Diseases	05020 Prion diseases [PATH:ko05020]	stress-induced-phosphoprotein 1	K09553	1	0	0	0	0
Metabolism	Amino Acid Metabolism	00250 Alanine, aspartate and glutamate metabolism [PATH:ko00250]	adenylosuccinate lyase [EC:4.3.2.2]	K01756	1	0	3	2	0
Metabolism	Amino Acid Metabolism	00250 Alanine, aspartate and glutamate metabolism [PATH:ko00250]	adenylosuccinate synthase [EC:6.3.4.4]	K01939	0	2	4	4	5
Metabolism	Amino Acid Metabolism	00250 Alanine, aspartate and glutamate metabolism [PATH:ko00250]	amidophosphoribosyltransferase [EC:2.4.2.14]	K00764	3	2	2	8	4
Metabolism	Amino Acid Metabolism	00250 Alanine, aspartate and glutamate metabolism [PATH:ko00250]	aspartate carbamoyltransferase catalytic subunit	K00609	1	0	1	2	0

Level 1	Level 2	Level 3	Function	KEGG ID	Sequence Reads				
					Day17	Day24	Day31	Day38	Bulk Soil
Metabolism	Amino Acid Metabolism	00250 Alanine, aspartate and glutamate metabolism [PATH:ko00250]	aspartate racemase [EC:5.1.1.13]	K01779	0	1	0	0	0
Metabolism	Amino Acid Metabolism	00250 Alanine, aspartate and glutamate metabolism [PATH:ko00250]	carbamoyl-phosphate synthase large subunit [EC:6.3.5.5]	K01955	8	0	8	9	0
Metabolism	Amino Acid Metabolism	00250 Alanine, aspartate and glutamate metabolism [PATH:ko00250]	carbamoyl-phosphate synthase small subunit [EC:6.3.5.5]	K01956	2	6	2	1	0
Metabolism	Amino Acid Metabolism	00250 Alanine, aspartate and glutamate metabolism [PATH:ko00250]	glucosamine--fructose-6-phosphate aminotransferase	K00820	6	6	0	9	0
Metabolism	Amino Acid Metabolism	00250 Alanine, aspartate and glutamate metabolism [PATH:ko00250]	glutamate synthase (NADPH/NADH) large chain [EC:1.4.1.13	K00265	6	6	11	10	11
Metabolism	Amino Acid Metabolism	00250 Alanine, aspartate and glutamate metabolism [PATH:ko00250]	glutamate synthase (NADPH/NADH) small chain [EC:1.4.1.13	K00266	0	8	0	0	0
Metabolism	Amino Acid Metabolism	00250 Alanine, aspartate and glutamate metabolism [PATH:ko00250]	L-aspartate oxidase [EC:1.4.3.16]	K00278	1	0	0	7	0
Metabolism	Amino Acid Metabolism	00260 Glycine, serine and threonine metabolism [PATH:ko00260]	5-aminolevulinate synthase [EC:2.3.1.37]	K00643	0	0	1	1	0

Level 1	Level 2	Level 3	Function	KEGG ID	Sequence Reads				
					Day17	Day24	Day31	Day38	Bulk Soil
Metabolism	Amino Acid Metabolism	00260 Glycine, serine and threonine metabolism [PATH:ko00260]	betaine-aldehyde dehydrogenase [EC:1.2.1.8]	K00130	2	5	2	0	0
Metabolism	Amino Acid Metabolism	00260 Glycine, serine and threonine metabolism [PATH:ko00260]	choline dehydrogenase [EC:1.1.99.1]	K00108	0	0	2	3	0
Metabolism	Amino Acid Metabolism	00260 Glycine, serine and threonine metabolism [PATH:ko00260]	D-3-phosphoglycerate dehydrogenase [EC:1.1.1.95]	K00058	0	0	0	6	0
Metabolism	Amino Acid Metabolism	00260 Glycine, serine and threonine metabolism [PATH:ko00260]	dimethylglycine dehydrogenase [EC:1.5.99.2]	K00315	0	0	0	0	1
Metabolism	Amino Acid Metabolism	00260 Glycine, serine and threonine metabolism [PATH:ko00260]	ectoine hydroxylase [EC:1.14.11.-]	K10674	1	0	0	0	1
Metabolism	Amino Acid Metabolism	00260 Glycine, serine and threonine metabolism [PATH:ko00260]	glycerate kinase [EC:2.7.1.31]	K00865	0	0	0	3	0
Metabolism	Amino Acid Metabolism	00260 Glycine, serine and threonine metabolism [PATH:ko00260]	glycine C-acetyltransferase [EC:2.3.1.29]	K00639	2	0	0	4	0
Metabolism	Amino Acid Metabolism	00260 Glycine, serine and threonine metabolism [PATH:ko00260]	glycine dehydrogenase [EC:1.4.4.2]	K00281	4	2	0	3	3
Metabolism	Amino Acid Metabolism	00260 Glycine, serine and threonine metabolism [PATH:ko00260]	glycine dehydrogenase subunit 1 [EC:1.4.4.2]	K00282	0	0	0	0	1
Metabolism	Amino Acid Metabolism	00260 Glycine, serine and threonine metabolism [PATH:ko00260]	glycine dehydrogenase subunit 2 [EC:1.4.4.2]	K00283	0	2	0	0	4

Level 1	Level 2	Level 3	Function	KEGG ID	Sequence Reads				
					Day17	Day24	Day31	Day38	Bulk Soil
Metabolism	Amino Acid Metabolism	00260 Glycine, serine and threonine metabolism [PATH:ko00260]	homoserine kinase [EC:2.7.1.39]	K00872	1	0	0	2	0
Metabolism	Amino Acid Metabolism	00260 Glycine, serine and threonine metabolism [PATH:ko00260]	hydroxypyruvate reductase [EC:1.1.1.81]	K00050	0	0	0	0	2
Metabolism	Amino Acid Metabolism	00260 Glycine, serine and threonine metabolism [PATH:ko00260]	L-ectoine synthase [EC:4.2.1.108]	K06720	0	0	1	0	0
Metabolism	Amino Acid Metabolism	00260 Glycine, serine and threonine metabolism [PATH:ko00260]	phosphatidylserine synthase [EC:2.7.8.8]	K00998	0	3	0	3	0
Metabolism	Amino Acid Metabolism	00260 Glycine, serine and threonine metabolism [PATH:ko00260]	phosphoserine / homoserine phosphotransferase [EC:3.1.3.3	K02203	0	0	0	1	0
Metabolism	Amino Acid Metabolism	00260 Glycine, serine and threonine metabolism [PATH:ko00260]	phosphoserine phosphatase [EC:3.1.3.3]	K01079	0	0	0	0	1
Metabolism	Amino Acid Metabolism	00260 Glycine, serine and threonine metabolism [PATH:ko00260]	sarcosine oxidase, subunit alpha [EC:1.5.3.1]	K00302	0	1	1	2	3
Metabolism	Amino Acid Metabolism	00260 Glycine, serine and threonine metabolism [PATH:ko00260]	sarcosine oxidase, subunit beta [EC:1.5.3.1]	K00303	0	1	1	3	0
Metabolism	Amino Acid Metabolism	00260 Glycine, serine and threonine metabolism [PATH:ko00260]	threonine 3-dehydrogenase [EC:1.1.1.103]	K00060	0	0	1	1	0
Metabolism	Amino Acid Metabolism	00260 Glycine, serine and threonine metabolism [PATH:ko00260]	threonine aldolase [EC:4.1.2.5]	K01620	3	0	0	0	0

Level 1	Level 2	Level 3	Function	KEGG ID	Sequence Reads				
					Day17	Day24	Day31	Day38	Bulk Soil
Metabolism	Amino Acid Metabolism	00260 Glycine, serine and threonine metabolism [PATH:ko00260]	threonine dehydratase [EC:4.3.1.19]	K01754	0	0	0	7	0
Metabolism	Amino Acid Metabolism	00270 Cysteine and methionine metabolism [PATH:ko00270]	(R)-2-hydroxyacid dehydrogenase [EC:1.1.1.272]	K05884	0	0	0	1	0
Metabolism	Amino Acid Metabolism	00270 Cysteine and methionine metabolism [PATH:ko00270]	5-methyltetrahydropteroyltriglutamate--homocysteine	K00549	0	3	0	0	5
Metabolism	Amino Acid Metabolism	00270 Cysteine and methionine metabolism [PATH:ko00270]	D-cysteine desulfhydrase [EC:4.4.1.15]	K05396	0	1	0	0	0
Metabolism	Amino Acid Metabolism	00270 Cysteine and methionine metabolism [PATH:ko00270]	homoserine dehydrogenase [EC:1.1.1.3]	K00003	0	0	3	0	0
Metabolism	Amino Acid Metabolism	00270 Cysteine and methionine metabolism [PATH:ko00270]	homoserine O-acetyltransferase [EC:2.3.1.31]	K00641	0	0	2	0	0
Metabolism	Amino Acid Metabolism	00270 Cysteine and methionine metabolism [PATH:ko00270]	L-serine dehydratase [EC:4.3.1.17]	K01752	1	2	1	0	2
Metabolism	Amino Acid Metabolism	00270 Cysteine and methionine metabolism [PATH:ko00270]	O-acetylhomoserine (thiol)-lyase [EC:2.5.1.49]	K01740	0	2	4	3	0
Metabolism	Amino Acid Metabolism	00270 Cysteine and methionine metabolism [PATH:ko00270]	O-succinylhomoserine sulfhydrylase [EC:2.5.1.-]	K10764	0	0	0	0	2
Metabolism	Amino Acid Metabolism	00270 Cysteine and methionine metabolism [PATH:ko00270]	S-adenosylhomocysteine/5'-methylthioadenosine nucleosidase	K01243	0	1	0	0	0

Level 1	Level 2	Level 3	Function	KEGG ID	Sequence Reads				
					Day17	Day24	Day31	Day38	Bulk Soil
Metabolism	Amino Acid Metabolism	00280 Valine, leucine and isoleucine degradation [PATH:ko00280]	2-oxoisovalerate dehydrogenase E2 component (dihydrolipoyl	K09699	0	2	0	0	0
Metabolism	Amino Acid Metabolism	00280 Valine, leucine and isoleucine degradation [PATH:ko00280]	3-hydroxyisobutyrate dehydrogenase [EC:1.1.1.31]	K00020	2	0	0	2	2
Metabolism	Amino Acid Metabolism	00280 Valine, leucine and isoleucine degradation [PATH:ko00280]	3-methylcrotonyl-CoA carboxylase alpha subunit [EC:6.4.1.4]	K01968	0	0	0	0	4
Metabolism	Amino Acid Metabolism	00280 Valine, leucine and isoleucine degradation [PATH:ko00280]	3-methylcrotonyl-CoA carboxylase beta subunit [EC:6.4.1.4]	K01969	0	1	3	0	6
Metabolism	Amino Acid Metabolism	00280 Valine, leucine and isoleucine degradation [PATH:ko00280]	3-oxoacid CoA-transferase subunit A [EC:2.8.3.5]	K01028	0	0	0	1	0
Metabolism	Amino Acid Metabolism	00280 Valine, leucine and isoleucine degradation [PATH:ko00280]	3-oxoacid CoA-transferase subunit B [EC:2.8.3.5]	K01029	0	0	1	0	0
Metabolism	Amino Acid Metabolism	00280 Valine, leucine and isoleucine degradation [PATH:ko00280]	butyryl-CoA dehydrogenase [EC:1.3.99.2]	K00248	4	0	0	2	6
Metabolism	Amino Acid Metabolism	00280 Valine, leucine and isoleucine degradation [PATH:ko00280]	dihydrolipoamide dehydrogenase [EC:1.8.1.4]	K00382	12	5	0	0	9

Level 1	Level 2	Level 3	Function	KEGG ID	Sequence Reads				
					Day17	Day24	Day31	Day38	Bulk Soil
Metabolism	Amino Acid Metabolism	00280 Valine, leucine and isoleucine degradation [PATH:ko00280]	isovaleryl-CoA dehydrogenase [EC:1.3.99.10]	K00253	0	0	0	1	0
Metabolism	Amino Acid Metabolism	00280 Valine, leucine and isoleucine degradation [PATH:ko00280]	methylglutaconyl-CoA hydratase [EC:4.2.1.18]	K13766	0	0	1	0	0
Metabolism	Amino Acid Metabolism	00280 Valine, leucine and isoleucine degradation [PATH:ko00280]	methylmalonate-semialdehyde dehydrogenase [EC:1.2.1.27]	K00140	3	0	4	4	2
Metabolism	Amino Acid Metabolism	00280 Valine, leucine and isoleucine degradation [PATH:ko00280]	methylmalonyl-CoA epimerase [EC:5.1.99.1]	K05606	0	0	0	1	1
Metabolism	Amino Acid Metabolism	00280 Valine, leucine and isoleucine degradation [PATH:ko00280]	methylmalonyl-CoA mutase [EC:5.4.99.2]	K01847	5	1	1	0	4
Metabolism	Amino Acid Metabolism	00280 Valine, leucine and isoleucine degradation [PATH:ko00280]	methylmalonyl-CoA mutase [EC:5.4.99.2]	K11942	0	0	0	2	0
Metabolism	Amino Acid Metabolism	00280 Valine, leucine and isoleucine degradation [PATH:ko00280]	methylmalonyl-CoA mutase, C-terminal domain [EC:5.4.99.2]	K01849	0	0	1	0	0
Metabolism	Amino Acid Metabolism	00280 Valine, leucine and isoleucine degradation [PATH:ko00280]	methylmalonyl-CoA mutase, N-terminal domain [EC:5.4.99.2]	K01848	0	6	0	4	1

Level 1	Level 2	Level 3	Function	KEGG ID	Sequence Reads				
					Day17	Day24	Day31	Day38	Bulk Soil
Metabolism	Amino Acid Metabolism	00280 Valine, leucine and isoleucine degradation [PATH:ko00280]	propionyl-CoA carboxylase alpha chain [EC:6.4.1.3]	K01965	2	2	3	0	0
Metabolism	Amino Acid Metabolism	00280 Valine, leucine and isoleucine degradation [PATH:ko00280]	propionyl-CoA carboxylase beta chain [EC:6.4.1.3]	K01966	4	6	6	11	6
Metabolism	Amino Acid Metabolism	00280 Valine, leucine and isoleucine degradation [PATH:ko00280]	short/branched chain acyl-CoA dehydrogenase [EC:1.3.99.12]	K11410	1	0	0	0	0
Metabolism	Amino Acid Metabolism	00290 Valine, leucine and isoleucine biosynthesis [PATH:ko00290]	2-isopropylmalate synthase [EC:2.3.3.13]	K01649	7	5	7	0	9
Metabolism	Amino Acid Metabolism	00290 Valine, leucine and isoleucine biosynthesis [PATH:ko00290]	3-isopropylmalate dehydrogenase [EC:1.1.1.85]	K00052	3	5	3	4	6
Metabolism	Amino Acid Metabolism	00290 Valine, leucine and isoleucine biosynthesis [PATH:ko00290]	3-isopropylmalate/(R)-2-methylmalate dehydratase large	K01703	5	4	7	5	3
Metabolism	Amino Acid Metabolism	00290 Valine, leucine and isoleucine biosynthesis [PATH:ko00290]	3-isopropylmalate/(R)-2-methylmalate dehydratase small	K01704	0	3	2	4	4
Metabolism	Amino Acid Metabolism	00290 Valine, leucine and isoleucine biosynthesis [PATH:ko00290]	acetolactate synthase I/II/III large subunit [EC:2.2.1.6]	K01652	5	11	5	9	11

Level 1	Level 2	Level 3	Function	KEGG ID	Sequence Reads				
					Day17	Day24	Day31	Day38	Bulk Soil
Metabolism	Amino Acid Metabolism	00290 Valine, leucine and isoleucine biosynthesis [PATH:ko00290]	alanine-synthesizing transaminase [EC:2.6.1.66 2.6.1.2]	K14260	0	2	0	0	0
Metabolism	Amino Acid Metabolism	00290 Valine, leucine and isoleucine biosynthesis [PATH:ko00290]	D-citramalate synthase [EC:2.3.1.182]	K09011	0	0	0	0	1
Metabolism	Amino Acid Metabolism	00290 Valine, leucine and isoleucine biosynthesis [PATH:ko00290]	leucine dehydrogenase [EC:1.4.1.9]	K00263	0	0	1	1	0
Metabolism	Amino Acid Metabolism	00290 Valine, leucine and isoleucine biosynthesis [PATH:ko00290]	pyruvate dehydrogenase E1 component [EC:1.2.4.1]	K00163	4	6	2	0	10
Metabolism	Amino Acid Metabolism	00290 Valine, leucine and isoleucine biosynthesis [PATH:ko00290]	pyruvate dehydrogenase E1 component subunit alpha	K00161	8	0	4	3	0
Metabolism	Amino Acid Metabolism	00290 Valine, leucine and isoleucine biosynthesis [PATH:ko00290]	pyruvate dehydrogenase E1 component subunit beta	K00162	0	0	0	4	0
Metabolism	Amino Acid Metabolism	00300 Lysine biosynthesis [PATH:ko00300]	2,3,4,5-tetrahydropyridine-2-carboxylate	K00674	0	2	4	2	0
Metabolism	Amino Acid Metabolism	00300 Lysine biosynthesis [PATH:ko00300]	aspartate kinase [EC:2.7.2.4]	K00928	2	0	3	3	3
Metabolism	Amino Acid Metabolism	00300 Lysine biosynthesis [PATH:ko00300]	aspartate-semialdehyde dehydrogenase [EC:1.2.1.11]	K00133	0	0	2	1	0
Metabolism	Amino Acid Metabolism	00300 Lysine biosynthesis	bifunctional aspartokinase / homoserine dehydrogenase 1	K12524	0	0	0	0	1

Level 1	Level 2	Level 3	Function	KEGG ID	Sequence Reads				
					Day17	Day24	Day31	Day38	Bulk Soil
		[PATH:ko00300]							
Metabolism	Amino Acid Metabolism	00300 Lysine biosynthesis [PATH:ko00300]	diaminopimelate decarboxylase [EC:4.1.1.20]	K01586	0	0	7	0	0
Metabolism	Amino Acid Metabolism	00300 Lysine biosynthesis [PATH:ko00300]	diaminopimelate epimerase [EC:5.1.1.7]	K01778	0	2	0	0	2
Metabolism	Amino Acid Metabolism	00300 Lysine biosynthesis [PATH:ko00300]	dihydrodipicolinate reductase [EC:1.3.1.26]	K00215	0	0	0	0	2
Metabolism	Amino Acid Metabolism	00300 Lysine biosynthesis [PATH:ko00300]	dihydrodipicolinate synthase [EC:4.2.1.52]	K01714	0	5	4	4	5
Metabolism	Amino Acid Metabolism	00300 Lysine biosynthesis [PATH:ko00300]	LL-diaminopimelate aminotransferase [EC:2.6.1.83]	K10206	1	0	0	0	0
Metabolism	Amino Acid Metabolism	00300 Lysine biosynthesis [PATH:ko00300]	L-lysine 6-transaminase [EC:2.6.1.36]	K03918	0	0	0	0	1
Metabolism	Amino Acid Metabolism	00300 Lysine biosynthesis [PATH:ko00300]	N-succinyldiaminopimelate aminotransferase [EC:2.6.1.17]	K14267	1	0	0	0	0
Metabolism	Amino Acid Metabolism	00300 Lysine biosynthesis [PATH:ko00300]	succinyl-diaminopimelate desuccinylase [EC:3.5.1.18]	K01439	0	0	0	5	0
Metabolism	Amino Acid Metabolism	00300 Lysine biosynthesis [PATH:ko00300]	tetrahydrodipicolinate N-acetyltransferase [EC:2.3.1.89]	K05822	0	0	1	0	0
Metabolism	Amino Acid Metabolism	00310 Lysine degradation [PATH:ko00310]	2-oxoglutarate dehydrogenase E2 component (dihydrolipoamide	K00658	0	5	1	7	0
Metabolism	Amino Acid Metabolism	00310 Lysine degradation [PATH:ko00310]	5-aminovalerate aminotransferase [EC:2.6.1.48]	K14268	0	0	0	0	1
Metabolism	Amino Acid Metabolism	00310 Lysine degradation [PATH:ko00310]	beta-lysine 5,6-aminomutase [EC:5.4.3.3]	K01844	0	3	1	1	0
Metabolism	Amino Acid Metabolism	00310 Lysine degradation [PATH:ko00310]	lysine 2,3-aminomutase [EC:5.4.3.2]	K01843	0	2	0	0	1

Level 1	Level 2	Level 3	Function	KEGG ID	Sequence Reads				
					Day17	Day24	Day31	Day38	Bulk Soil
Metabolism	Amino Acid Metabolism	00310 Lysine degradation [PATH:ko00310]	lysine N6-hydroxylase [EC:1.14.13.59]	K03897	1	0	0	0	0
Metabolism	Amino Acid Metabolism	00330 Arginine and proline metabolism [PATH:ko00330]	1-pyrroline-5-carboxylate dehydrogenase [EC:1.5.1.12]	K00294	0	7	2	2	2
Metabolism	Amino Acid Metabolism	00330 Arginine and proline metabolism [PATH:ko00330]	acetylglutamate/acetylaminoadipate kinase [EC:2.7.2.8	K00930	1	2	0	0	0
Metabolism	Amino Acid Metabolism	00330 Arginine and proline metabolism [PATH:ko00330]	acetylornithine aminotransferase [EC:2.6.1.11]	K00818	1	3	3	0	0
Metabolism	Amino Acid Metabolism	00330 Arginine and proline metabolism [PATH:ko00330]	acetylornithine deacetylase [EC:3.5.1.16]	K01438	0	0	0	2	0
Metabolism	Amino Acid Metabolism	00330 Arginine and proline metabolism [PATH:ko00330]	acetylornithine/N-succinyldiaminopimelate aminotransferase	K00821	0	2	0	2	1
Metabolism	Amino Acid Metabolism	00330 Arginine and proline metabolism [PATH:ko00330]	agmatinase [EC:3.5.3.11]	K01480	2	4	0	5	0
Metabolism	Amino Acid Metabolism	00330 Arginine and proline metabolism [PATH:ko00330]	amino-acid N-acetyltransferase [EC:2.3.1.1]	K00619	0	1	0	0	0
Metabolism	Amino Acid Metabolism	00330 Arginine and proline metabolism [PATH:ko00330]	arginine decarboxylase [EC:4.1.1.19]	K01584	1	0	0	2	0
Metabolism	Amino Acid Metabolism	00330 Arginine and proline metabolism [PATH:ko00330]	arginine decarboxylase [EC:4.1.1.19]	K01585	0	1	0	0	0
Metabolism	Amino Acid Metabolism	00330 Arginine and proline metabolism [PATH:ko00330]	arginine deiminase [EC:3.5.3.6]	K01478	0	1	1	0	0
Metabolism	Amino Acid Metabolism	00330 Arginine and proline metabolism [PATH:ko00330]	argininosuccinate lyase [EC:4.3.2.1]	K01755	0	5	5	0	3
Metabolism	Amino Acid Metabolism	00330 Arginine and proline metabolism [PATH:ko00330]	argininosuccinate synthase [EC:6.3.4.5]	K01940	0	3	5	2	5

Level 1	Level 2	Level 3	Function	KEGG ID	Sequence Reads				
					Day17	Day24	Day31	Day38	Bulk Soil
Metabolism	Amino Acid Metabolism	00330 Arginine and proline metabolism [PATH:ko00330]	carbamate kinase [EC:2.7.2.2]	K00926	0	2	0	0	0
Metabolism	Amino Acid Metabolism	00330 Arginine and proline metabolism [PATH:ko00330]	creatinase [EC:3.5.3.3]	K08688	0	0	0	1	0
Metabolism	Amino Acid Metabolism	00330 Arginine and proline metabolism [PATH:ko00330]	cytosine deaminase [EC:3.5.4.1]	K01485	0	1	0	6	0
Metabolism	Amino Acid Metabolism	00330 Arginine and proline metabolism [PATH:ko00330]	glutamate 5-kinase [EC:2.7.2.11]	K00931	0	0	2	0	0
Metabolism	Amino Acid Metabolism	00330 Arginine and proline metabolism [PATH:ko00330]	glutamate dehydrogenase (NADP+) [EC:1.4.1.4]	K00262	3	0	1	0	0
Metabolism	Amino Acid Metabolism	00330 Arginine and proline metabolism [PATH:ko00330]	glutamate dehydrogenase [EC:1.4.1.2]	K00260	0	8	0	0	4
Metabolism	Amino Acid Metabolism	00330 Arginine and proline metabolism [PATH:ko00330]	glutamate N-acetyltransferase / amino-acid	K00620	0	3	1	0	4
Metabolism	Amino Acid Metabolism	00330 Arginine and proline metabolism [PATH:ko00330]	glutamate-5-semialdehyde dehydrogenase [EC:1.2.1.41]	K00147	3	4	2	0	1
Metabolism	Amino Acid Metabolism	00330 Arginine and proline metabolism [PATH:ko00330]	N-acetyl-gamma-glutamyl-phosphate/N-acetyl-gamma-aminoadipyl	K00145	0	4	2	0	0
Metabolism	Amino Acid Metabolism	00330 Arginine and proline metabolism [PATH:ko00330]	N-carbamoylputrescine amidase [EC:3.5.1.53]	K12251	0	0	2	0	0
Metabolism	Amino Acid Metabolism	00330 Arginine and proline metabolism [PATH:ko00330]	N-methylhydantoinase A [EC:3.5.2.14]	K01473	1	7	0	0	0
Metabolism	Amino Acid Metabolism	00330 Arginine and proline metabolism [PATH:ko00330]	N-methylhydantoinase B [EC:3.5.2.14]	K01474	2	2	0	3	0
Metabolism	Amino Acid Metabolism	00330 Arginine and proline metabolism [PATH:ko00330]	ornithine carbamoyltransferase [EC:2.1.3.3]	K00611	1	3	2	0	3

Level 1	Level 2	Level 3	Function	KEGG ID	Sequence Reads				
					Day17	Day24	Day31	Day38	Bulk Soil
Metabolism	Amino Acid Metabolism	00330 Arginine and proline metabolism [PATH:ko00330]	ornithine cyclodeaminase [EC:4.3.1.12]	K01750	1	0	0	0	0
Metabolism	Amino Acid Metabolism	00330 Arginine and proline metabolism [PATH:ko00330]	ornithine--oxo-acid transaminase [EC:2.6.1.13]	K00819	0	0	1	0	0
Metabolism	Amino Acid Metabolism	00330 Arginine and proline metabolism [PATH:ko00330]	oxaloacetate decarboxylase, alpha subunit [EC:4.1.1.3]	K01571	1	0	1	0	0
Metabolism	Amino Acid Metabolism	00330 Arginine and proline metabolism [PATH:ko00330]	proline dehydrogenase / delta 1-pyrroline-5-carboxylate	K13821	0	0	4	0	0
Metabolism	Amino Acid Metabolism	00330 Arginine and proline metabolism [PATH:ko00330]	proline dehydrogenase [EC:1.5.99.8]	K00318	0	0	3	0	0
Metabolism	Amino Acid Metabolism	00330 Arginine and proline metabolism [PATH:ko00330]	proline racemase [EC:5.1.1.4]	K01777	0	0	0	1	0
Metabolism	Amino Acid Metabolism	00330 Arginine and proline metabolism [PATH:ko00330]	prolyl 4-hydroxylase [EC:1.14.11.2]	K00472	0	0	0	2	0
Metabolism	Amino Acid Metabolism	00330 Arginine and proline metabolism [PATH:ko00330]	putrescine aminotransferase [EC:2.6.1.-]	K12256	0	0	1	0	0
Metabolism	Amino Acid Metabolism	00330 Arginine and proline metabolism [PATH:ko00330]	pyrroline-5-carboxylate reductase [EC:1.5.1.2]	K00286	0	0	1	0	2
Metabolism	Amino Acid Metabolism	00330 Arginine and proline metabolism [PATH:ko00330]	S-adenosylmethionine decarboxylase [EC:4.1.1.50]	K01611	2	0	1	2	0
Metabolism	Amino Acid Metabolism	00330 Arginine and proline metabolism [PATH:ko00330]	succinylglutamic semialdehyde dehydrogenase [EC:1.2.1.71]	K06447	0	0	0	2	0
Metabolism	Amino Acid Metabolism	00330 Arginine and proline metabolism [PATH:ko00330]	urease [EC:3.5.1.5]	K01427	0	0	0	0	1
Metabolism	Amino Acid Metabolism	00340 Histidine metabolism [PATH:ko00340]	ATP phosphoribosyltransferase regulatory subunit	K02502	0	0	0	3	0

Level 1	Level 2	Level 3	Function	KEGG ID	Sequence Reads				
					Day17	Day24	Day31	Day38	Bulk Soil
Metabolism	Amino Acid Metabolism	00340 Histidine metabolism [PATH:ko00340]	cyclase [EC:4.1.3.-]	K02500	0	0	1	1	1
Metabolism	Amino Acid Metabolism	00340 Histidine metabolism [PATH:ko00340]	glutamine amidotransferase [EC:2.4.2.-]	K02501	0	1	1	0	2
Metabolism	Amino Acid Metabolism	00340 Histidine metabolism [PATH:ko00340]	histidine ammonia-lyase [EC:4.3.1.3]	K01745	0	8	0	0	0
Metabolism	Amino Acid Metabolism	00340 Histidine metabolism [PATH:ko00340]	histidinol dehydrogenase [EC:1.1.1.23]	K00013	3	5	2	3	0
Metabolism	Amino Acid Metabolism	00340 Histidine metabolism [PATH:ko00340]	imidazoleglycerol-phosphate dehydratase [EC:4.2.1.19]	K01693	0	0	0	2	0
Metabolism	Amino Acid Metabolism	00340 Histidine metabolism [PATH:ko00340]	imidazolonepropionase [EC:3.5.2.7]	K01468	0	0	1	1	0
Metabolism	Amino Acid Metabolism	00340 Histidine metabolism [PATH:ko00340]	N-formylglutamate deformylase [EC:3.5.1.68]	K01458	0	0	0	0	1
Metabolism	Amino Acid Metabolism	00340 Histidine metabolism [PATH:ko00340]	phosphoribosyl-AMP cyclohydrolase [EC:3.5.4.19]	K01496	1	0	0	0	0
Metabolism	Amino Acid Metabolism	00340 Histidine metabolism [PATH:ko00340]	phosphoribosyl-ATP pyrophosphohydrolase / phosphoribosyl-AMP	K11755	0	0	0	0	1
Metabolism	Amino Acid Metabolism	00340 Histidine metabolism [PATH:ko00340]	phosphoribosyl-ATP pyrophosphohydrolase [EC:3.6.1.31]	K01523	0	0	2	0	0
Metabolism	Amino Acid Metabolism	00340 Histidine metabolism [PATH:ko00340]	phosphoribosylformimino-5-aminoimidazole carboxamide	K01814	1	0	1	4	0
Metabolism	Amino Acid Metabolism	00340 Histidine metabolism [PATH:ko00340]	urocanate hydratase [EC:4.2.1.49]	K01712	0	2	5	5	6
Metabolism	Amino Acid Metabolism	00350 Tyrosine metabolism [PATH:ko00350]	[EC:1.13.12.-]	K00468	0	1	0	0	0

Level 1	Level 2	Level 3	Function	KEGG ID	Sequence Reads				
					Day17	Day24	Day31	Day38	Bulk Soil
Metabolism	Amino Acid Metabolism	00350 Tyrosine metabolism [PATH:ko00350]	2,4-dihydroxyhept-2-ene-1,7-dioic acid aldolase [EC:4.1.2.-]	K02510	0	3	2	1	0
Metabolism	Amino Acid Metabolism	00350 Tyrosine metabolism [PATH:ko00350]	2-oxo-hept-3-ene-1,7-dioate hydratase [EC:4.2.1.-]	K02509	0	2	0	0	0
Metabolism	Amino Acid Metabolism	00350 Tyrosine metabolism [PATH:ko00350]	4-hydroxyphenylacetate-3-hydroxylase large chain	K00483	0	0	1	0	1
Metabolism	Amino Acid Metabolism	00350 Tyrosine metabolism [PATH:ko00350]	5-oxopent-3-ene-1,2,5-tricarboxylate decarboxylase /	K05921	0	0	1	0	0
Metabolism	Amino Acid Metabolism	00350 Tyrosine metabolism [PATH:ko00350]	gentisate 1,2-dioxygenase [EC:1.13.11.4]	K00450	1	0	0	0	2
Metabolism	Amino Acid Metabolism	00350 Tyrosine metabolism [PATH:ko00350]	succinate-semialdehyde dehydrogenase (NADP+) [EC:1.2.1.16]	K00135	2	0	1	4	0
Metabolism	Amino Acid Metabolism	00360 Phenylalanine metabolism [PATH:ko00360]	2-hydroxy-6-ketonona-2,4-dienedioic acid hydrolase	K05714	1	0	0	0	0
Metabolism	Amino Acid Metabolism	00360 Phenylalanine metabolism [PATH:ko00360]	3-(3-hydroxy-phenyl)propionate hydroxylase [EC:1.14.13.-]	K05712	0	0	0	2	0
Metabolism	Amino Acid Metabolism	00360 Phenylalanine metabolism [PATH:ko00360]	D-amino-acid dehydrogenase [EC:1.4.99.1]	K00285	0	3	0	0	0
Metabolism	Amino Acid Metabolism	00360 Phenylalanine metabolism [PATH:ko00360]	ferredoxin subunit of phenylpropionate dioxygenase	K05710	0	0	1	1	0
Metabolism	Amino Acid Metabolism	00360 Phenylalanine metabolism [PATH:ko00360]	hippurate hydrolase [EC:3.5.1.32]	K01451	0	0	0	2	0
Metabolism	Amino Acid Metabolism	00360 Phenylalanine metabolism [PATH:ko00360]	large terminal subunit of phenylpropionate dioxygenase	K05708	0	0	1	0	0
Metabolism	Amino Acid Metabolism	00360 Phenylalanine metabolism [PATH:ko00360]	phenylacetate-CoA ligase [EC:6.2.1.30]	K01912	0	1	0	0	2

Level 1	Level 2	Level 3	Function	KEGG ID	Sequence Reads				
					Day17	Day24	Day31	Day38	Bulk Soil
Metabolism	Amino Acid Metabolism	00380 Tryptophan metabolism [PATH:ko00380]	2-oxoglutarate dehydrogenase E1 component [EC:1.2.4.2]	K00164	9	0	13	0	0
Metabolism	Amino Acid Metabolism	00380 Tryptophan metabolism [PATH:ko00380]	3-hydroxyanthranilate 3,4-dioxygenase [EC:1.13.11.6]	K00452	0	0	0	1	1
Metabolism	Amino Acid Metabolism	00380 Tryptophan metabolism [PATH:ko00380]	kynureninase [EC:3.7.1.3]	K01556	0	1	0	0	0
Metabolism	Amino Acid Metabolism	00400 Phenylalanine, tyrosine and tryptophan biosynthesis [PATH:ko00400]	3-dehydroquinate dehydratase II [EC:4.2.1.10]	K03786	0	0	4	0	1
Metabolism	Amino Acid Metabolism	00400 Phenylalanine, tyrosine and tryptophan biosynthesis [PATH:ko00400]	3-dehydroquinate synthase [EC:4.2.3.4]	K01735	1	0	2	0	0
Metabolism	Amino Acid Metabolism	00400 Phenylalanine, tyrosine and tryptophan biosynthesis [PATH:ko00400]	3-deoxy-7-phosphoheptulonate synthase [EC:2.5.1.54]	K03856	3	0	0	1	0
Metabolism	Amino Acid Metabolism	00400 Phenylalanine, tyrosine and tryptophan biosynthesis [PATH:ko00400]	3-phosphoshikimate 1-carboxyvinyltransferase [EC:2.5.1.19]	K00800	0	0	0	3	0
Metabolism	Amino Acid Metabolism	00400 Phenylalanine, tyrosine and tryptophan biosynthesis [PATH:ko00400]	anthranilate phosphoribosyltransferase [EC:2.4.2.18]	K00766	0	0	0	0	3

Level 1	Level 2	Level 3	Function	KEGG ID	Sequence Reads				
					Day17	Day24	Day31	Day38	Bulk Soil
Metabolism	Amino Acid Metabolism	00400 Phenylalanine, tyrosine and tryptophan biosynthesis [PATH:ko00400]	anthranilate synthase [EC:4.1.3.27]	K13503	0	2	0	1	0
Metabolism	Amino Acid Metabolism	00400 Phenylalanine, tyrosine and tryptophan biosynthesis [PATH:ko00400]	anthranilate synthase component I [EC:4.1.3.27]	K01657	0	0	0	6	0
Metabolism	Amino Acid Metabolism	00400 Phenylalanine, tyrosine and tryptophan biosynthesis [PATH:ko00400]	chorismate synthase [EC:4.2.3.5]	K01736	5	0	2	0	5
Metabolism	Amino Acid Metabolism	00400 Phenylalanine, tyrosine and tryptophan biosynthesis [PATH:ko00400]	indole-3-glycerol phosphate synthase [EC:4.1.1.48]	K01609	0	1	0	2	0
Metabolism	Amino Acid Metabolism	00400 Phenylalanine, tyrosine and tryptophan biosynthesis [PATH:ko00400]	phosphoribosylanthranilate isomerase [EC:5.3.1.24]	K01817	0	0	0	1	0
Metabolism	Amino Acid Metabolism	00400 Phenylalanine, tyrosine and tryptophan biosynthesis [PATH:ko00400]	shikimate dehydrogenase [EC:1.1.1.25]	K00014	0	0	0	0	1
Metabolism	Amino Acid Metabolism	00400 Phenylalanine, tyrosine and tryptophan biosynthesis	shikimate kinase [EC:2.7.1.71]	K00891	0	0	0	0	3

Level 1	Level 2	Level 3	Function	KEGG ID	Sequence Reads				
					Day17	Day24	Day31	Day38	Bulk Soil
		[PATH:ko00400]							
Metabolism	Amino Acid Metabolism	00400 Phenylalanine, tyrosine and tryptophan biosynthesis [PATH:ko00400]	tryptophan synthase beta chain [EC:4.2.1.20]	K01696	0	0	3	1	0
Metabolism	Biosynthesis of Other Secondary Metabolites	00401 Novobiocin biosynthesis [PATH:ko00401]	aspartate aminotransferase [EC:2.6.1.1]	K00811	1	0	0	0	0
Metabolism	Biosynthesis of Other Secondary Metabolites	00401 Novobiocin biosynthesis [PATH:ko00401]	aspartate aminotransferase [EC:2.6.1.1]	K00812	0	3	5	0	0
Metabolism	Biosynthesis of Other Secondary Metabolites	00401 Novobiocin biosynthesis [PATH:ko00401]	histidinol-phosphate aminotransferase [EC:2.6.1.9]	K00817	0	6	0	5	0
Metabolism	Biosynthesis of Other Secondary Metabolites	00401 Novobiocin biosynthesis [PATH:ko00401]	prephenate dehydrogenase [EC:1.3.1.12]	K04517	0	1	3	0	0
Metabolism	Biosynthesis of Other Secondary Metabolites	00521 Streptomycin biosynthesis [PATH:ko00521]	dTDP-4-dehydrorhamnose 3,5-epimerase [EC:5.1.3.13]	K01790	0	1	0	0	0
Metabolism	Biosynthesis of Other Secondary Metabolites	00521 Streptomycin biosynthesis [PATH:ko00521]	dTDP-4-dehydrorhamnose reductase [EC:1.1.1.133]	K00067	0	2	0	0	0
Metabolism	Biosynthesis of Other Secondary Metabolites	00521 Streptomycin biosynthesis [PATH:ko00521]	dTDP-glucose 4,6-dehydratase [EC:4.2.1.46]	K01710	0	0	7	8	5

Level 1	Level 2	Level 3	Function	KEGG ID	Sequence Reads				
					Day17	Day24	Day31	Day38	Bulk Soil
Metabolism	Biosynthesis of Other Secondary Metabolites	00521 Streptomycin biosynthesis [PATH:ko00521]	glucose-1-phosphate thymidylyltransferase [EC:2.7.7.24]	K00973	0	0	2	0	0
Metabolism	Biosynthesis of Other Secondary Metabolites	00521 Streptomycin biosynthesis [PATH:ko00521]	myo-inositol 2-dehydrogenase [EC:1.1.1.18]	K00010	1	0	0	0	0
Metabolism	Biosynthesis of Other Secondary Metabolites	00521 Streptomycin biosynthesis [PATH:ko00521]	myo-inositol-1-phosphate synthase [EC:5.5.1.4]	K01858	3	0	0	3	4
Metabolism	Biosynthesis of Other Secondary Metabolites	00521 Streptomycin biosynthesis [PATH:ko00521]	phosphoglucomutase [EC:5.4.2.2]	K01835	0	0	0	2	0
Metabolism	Biosynthesis of Other Secondary Metabolites	00524 Butirosin and neomycin biosynthesis [PATH:ko00524]	glucokinase [EC:2.7.1.2]	K00845	0	0	4	0	1
Metabolism	Biosynthesis of Other Secondary Metabolites	00901 Indole alkaloid biosynthesis [PATH:ko00901]	strictosidine synthase [EC:4.3.3.2]	K01757	0	0	0	0	1
Metabolism	Biosynthesis of Other Secondary Metabolites	00940 Phenylpropanoid biosynthesis [PATH:ko00940]	beta-glucosidase [EC:3.2.1.21]	K05350	0	0	1	0	0
Metabolism	Biosynthesis of Other Secondary Metabolites	00940 Phenylpropanoid biosynthesis [PATH:ko00940]	catalase/peroxidase [EC:1.11.1.6 1.11.1.7]	K03782	4	0	3	3	6
Metabolism	Biosynthesis of Other Secondary Metabolites	00941 Flavonoid biosynthesis [PATH:ko00941]	caffeoyl-CoA O-methyltransferase [EC:2.1.1.104]	K00588	1	0	0	0	0

Level 1	Level 2	Level 3	Function	KEGG ID	Sequence Reads				
					Day17	Day24	Day31	Day38	Bulk Soil
Metabolism	Biosynthesis of Other Secondary Metabolites	00965 Betalain biosynthesis [PATH:ko00965]	aromatic-L-amino-acid decarboxylase [EC:4.1.1.28]	K01593	0	1	1	0	3
Metabolism	Carbohydrate Metabolism	00010 Glycolysis / Gluconeogenesis [PATH:ko00010]	pyruvate decarboxylase [EC:4.1.1.1]	K01568	0	0	1	0	0
Metabolism	Carbohydrate Metabolism	00020 Citrate cycle (TCA cycle) [PATH:ko00020]	isocitrate dehydrogenase (NAD+) [EC:1.1.1.41]	K00030	1	0	0	2	0
Metabolism	Carbohydrate Metabolism	00030 Pentose phosphate pathway [PATH:ko00030]	6-phosphogluconolactonase [EC:3.1.1.31]	K01057	0	0	1	0	0
Metabolism	Carbohydrate Metabolism	00030 Pentose phosphate pathway [PATH:ko00030]	deoxyribose-phosphate aldolase [EC:4.1.2.4]	K01619	0	0	3	0	0
Metabolism	Carbohydrate Metabolism	00030 Pentose phosphate pathway [PATH:ko00030]	gluconate 2-dehydrogenase gamma chain [EC:1.1.99.3]	K06152	0	0	0	1	0
Metabolism	Carbohydrate Metabolism	00030 Pentose phosphate pathway [PATH:ko00030]	gluconokinase [EC:2.7.1.12]	K00851	0	3	0	0	0
Metabolism	Carbohydrate Metabolism	00030 Pentose phosphate pathway [PATH:ko00030]	phosphogluconate dehydratase [EC:4.2.1.12]	K01690	0	0	0	1	0
Metabolism	Carbohydrate Metabolism	00030 Pentose phosphate pathway [PATH:ko00030]	transaldolase [EC:2.2.1.2]	K00616	6	0	4	0	0
Metabolism	Carbohydrate Metabolism	00040 Pentose and glucuronate interconversions [PATH:ko00040]	altronate hydrolase [EC:4.2.1.7]	K01685	0	1	1	0	2
Metabolism	Carbohydrate Metabolism	00040 Pentose and glucuronate interconversions [PATH:ko00040]	L-arabinose isomerase [EC:5.3.1.4]	K01804	0	0	1	0	0
Metabolism	Carbohydrate Metabolism	00040 Pentose and glucuronate interconversions [PATH:ko00040]	L-ribulokinase [EC:2.7.1.16]	K00853	0	1	0	0	0

Level 1	Level 2	Level 3	Function	KEGG ID	Sequence Reads				
					Day17	Day24	Day31	Day38	Bulk Soil
Metabolism	Carbohydrate Metabolism	00040 Pentose and glucuronate interconversions [PATH:ko00040]	xylulokinase [EC:2.7.1.17]	K00854	0	0	1	1	0
Metabolism	Carbohydrate Metabolism	00051 Fructose and mannose metabolism [PATH:ko00051]	[EC:3.1.3.-]	K01112	0	0	0	1	0
Metabolism	Carbohydrate Metabolism	00051 Fructose and mannose metabolism [PATH:ko00051]	L-fuculose-phosphate aldolase [EC:4.1.2.17]	K01628	0	0	1	0	0
Metabolism	Carbohydrate Metabolism	00051 Fructose and mannose metabolism [PATH:ko00051]	L-iditol 2-dehydrogenase [EC:1.1.1.14]	K00008	0	1	1	0	0
Metabolism	Carbohydrate Metabolism	00051 Fructose and mannose metabolism [PATH:ko00051]	L-rhamnose isomerase [EC:5.3.1.14]	K01813	1	0	0	0	2
Metabolism	Carbohydrate Metabolism	00051 Fructose and mannose metabolism [PATH:ko00051]	pyrophosphate--fructose-6-phosphate 1-phosphotransferase	K00895	0	1	0	0	0
Metabolism	Carbohydrate Metabolism	00051 Fructose and mannose metabolism [PATH:ko00051]	rhamnulose-1-phosphate aldolase [EC:4.1.2.19]	K01629	0	0	0	1	1
Metabolism	Carbohydrate Metabolism	00051 Fructose and mannose metabolism [PATH:ko00051]	xylose isomerase [EC:5.3.1.5]	K01805	0	2	0	0	0
Metabolism	Carbohydrate Metabolism	00052 Galactose metabolism [PATH:ko00052]	beta-galactosidase [EC:3.2.1.23]	K12308	0	2	0	0	0
Metabolism	Carbohydrate Metabolism	00052 Galactose metabolism [PATH:ko00052]	galactonate dehydratase [EC:4.2.1.6]	K01684	0	0	2	0	4

Level 1	Level 2	Level 3	Function	KEGG ID	Sequence Reads				
					Day17	Day24	Day31	Day38	Bulk Soil
Metabolism	Carbohydrate Metabolism	00052 Galactose metabolism [PATH:ko00052]	tagatose 1,6-diphosphate aldolase [EC:4.1.2.40]	K01635	0	0	0	0	1
Metabolism	Carbohydrate Metabolism	00053 Ascorbate and aldarate metabolism [PATH:ko00053]	2-dehydro-3-deoxyglucarate aldolase [EC:4.1.2.20]	K01630	0	0	0	1	0
Metabolism	Carbohydrate Metabolism	00053 Ascorbate and aldarate metabolism [PATH:ko00053]	D-threo-aldose 1-dehydrogenase [EC:1.1.1.122]	K00064	0	0	0	0	2
Metabolism	Carbohydrate Metabolism	00053 Ascorbate and aldarate metabolism [PATH:ko00053]	glucarate dehydratase [EC:4.2.1.40]	K01706	1	0	0	0	0
Metabolism	Carbohydrate Metabolism	00500 Starch and sucrose metabolism [PATH:ko00500]	1,4-alpha-glucan branching enzyme [EC:2.4.1.18]	K00700	2	0	0	4	0
Metabolism	Carbohydrate Metabolism	00500 Starch and sucrose metabolism [PATH:ko00500]	4-alpha-glucanotransferase [EC:2.4.1.25]	K00705	0	0	1	0	0
Metabolism	Carbohydrate Metabolism	00500 Starch and sucrose metabolism [PATH:ko00500]	alpha,alpha-trehalase [EC:3.2.1.28]	K01194	0	0	0	1	0
Metabolism	Carbohydrate Metabolism	00500 Starch and sucrose metabolism [PATH:ko00500]	alpha,alpha-trehalose-phosphate synthase (UDP-forming)	K00697	3	0	0	0	0
Metabolism	Carbohydrate Metabolism	00500 Starch and sucrose metabolism [PATH:ko00500]	alpha-amylase [EC:3.2.1.1]	K01176	1	0	0	0	0
Metabolism	Carbohydrate Metabolism	00500 Starch and sucrose metabolism [PATH:ko00500]	alpha-glucosidase [EC:3.2.1.20]	K01187	4	0	0	0	4
Metabolism	Carbohydrate Metabolism	00500 Starch and sucrose metabolism [PATH:ko00500]	amylosucrase [EC:2.4.1.4]	K05341	0	1	0	0	0
Metabolism	Carbohydrate Metabolism	00500 Starch and sucrose metabolism [PATH:ko00500]	beta-fructofuranosidase [EC:3.2.1.26]	K01193	0	0	1	0	0

Level 1	Level 2	Level 3	Function	KEGG ID	Sequence Reads				
					Day17	Day24	Day31	Day38	Bulk Soil
Metabolism	Carbohydrate Metabolism	00500 Starch and sucrose metabolism [PATH:ko00500]	cellulose synthase (UDP-forming) [EC:2.4.1.12]	K00694	0	0	0	2	0
Metabolism	Carbohydrate Metabolism	00500 Starch and sucrose metabolism [PATH:ko00500]	endoglucanase [EC:3.2.1.4]	K01179	0	4	0	0	4
Metabolism	Carbohydrate Metabolism	00500 Starch and sucrose metabolism [PATH:ko00500]	glucan 1,3-beta-glucosidase [EC:3.2.1.58]	K01210	0	0	0	1	0
Metabolism	Carbohydrate Metabolism	00500 Starch and sucrose metabolism [PATH:ko00500]	glucan endo-1,3-beta-D-glucosidase [EC:3.2.1.39]	K01199	0	0	0	0	1
Metabolism	Carbohydrate Metabolism	00500 Starch and sucrose metabolism [PATH:ko00500]	maltose alpha-D-glucosyltransferase [EC:5.4.99.16]	K05343	0	0	2	1	8
Metabolism	Carbohydrate Metabolism	00500 Starch and sucrose metabolism [PATH:ko00500]	oligo-1,6-glucosidase [EC:3.2.1.10]	K01182	1	1	0	1	0
Metabolism	Carbohydrate Metabolism	00500 Starch and sucrose metabolism [PATH:ko00500]	sucrose phosphorylase [EC:2.4.1.7]	K00690	0	0	1	1	0
Metabolism	Carbohydrate Metabolism	00520 Amino sugar and nucleotide sugar metabolism [PATH:ko00520]	CDP-6-deoxy-D-xylo-4-hexulose-3-dehydrase	K12452	0	0	0	2	0
Metabolism	Carbohydrate Metabolism	00520 Amino sugar and nucleotide sugar metabolism [PATH:ko00520]	CDP-paratose 2-epimerase [EC:5.1.3.10]	K12454	1	1	0	0	2
Metabolism	Carbohydrate Metabolism	00520 Amino sugar and nucleotide sugar metabolism [PATH:ko00520]	chitin synthase [EC:2.4.1.16]	K00698	0	0	0	1	0
Metabolism	Carbohydrate Metabolism	00520 Amino sugar and nucleotide sugar metabolism [PATH:ko00520]	galactokinase [EC:2.7.1.6]	K00849	1	0	0	0	1

Level 1	Level 2	Level 3	Function	KEGG ID	Sequence Reads				
					Day17	Day24	Day31	Day38	Bulk Soil
Metabolism	Carbohydrate Metabolism	00520 Amino sugar and nucleotide sugar metabolism [PATH:ko00520]	GDP-L-fucose synthase [EC:1.1.1.271]	K02377	0	3	0	3	0
Metabolism	Carbohydrate Metabolism	00520 Amino sugar and nucleotide sugar metabolism [PATH:ko00520]	GDPmannose 4,6-dehydratase [EC:4.2.1.47]	K01711	0	7	0	5	2
Metabolism	Carbohydrate Metabolism	00520 Amino sugar and nucleotide sugar metabolism [PATH:ko00520]	glucose-1-phosphate adenylyltransferase [EC:2.7.7.27]	K00975	0	0	2	0	3
Metabolism	Carbohydrate Metabolism	00520 Amino sugar and nucleotide sugar metabolism [PATH:ko00520]	glucose-6-phosphate isomerase [EC:5.3.1.9]	K01810	0	5	0	4	6
Metabolism	Carbohydrate Metabolism	00520 Amino sugar and nucleotide sugar metabolism [PATH:ko00520]	mannose-1-phosphate guanylyltransferase [EC:2.7.7.13]	K00966	0	0	3	1	0
Metabolism	Carbohydrate Metabolism	00520 Amino sugar and nucleotide sugar metabolism [PATH:ko00520]	mannose-1-phosphate guanylyltransferase [EC:2.7.7.22]	K00971	3	1	0	5	0
Metabolism	Carbohydrate Metabolism	00520 Amino sugar and nucleotide sugar metabolism [PATH:ko00520]	mannose-6-phosphate isomerase [EC:5.3.1.8]	K01809	0	1	0	5	0
Metabolism	Carbohydrate Metabolism	00520 Amino sugar and nucleotide sugar metabolism [PATH:ko00520]	N-acetylglucosamine-6-phosphate deacetylase [EC:3.5.1.25]	K01443	0	0	1	0	2
Metabolism	Carbohydrate Metabolism	00520 Amino sugar and nucleotide sugar metabolism [PATH:ko00520]	N-acetylneuraminate lyase [EC:4.1.3.3]	K01639	0	0	0	1	0

Level 1	Level 2	Level 3	Function	KEGG ID	Sequence Reads				
					Day17	Day24	Day31	Day38	Bulk Soil
Metabolism	Carbohydrate Metabolism	00520 Amino sugar and nucleotide sugar metabolism [PATH:ko00520]	N-acetylneuraminate synthase [EC:2.5.1.56]	K01654	0	0	1	0	0
Metabolism	Carbohydrate Metabolism	00520 Amino sugar and nucleotide sugar metabolism [PATH:ko00520]	NAD-dependent deacetylase [EC:3.5.1.-]	K12410	0	1	0	0	0
Metabolism	Carbohydrate Metabolism	00520 Amino sugar and nucleotide sugar metabolism [PATH:ko00520]	phosphoglucosamine mutase [EC:5.4.2.10]	K03431	0	4	3	5	2
Metabolism	Carbohydrate Metabolism	00520 Amino sugar and nucleotide sugar metabolism [PATH:ko00520]	phosphomannomutase [EC:5.4.2.8]	K01840	8	5	3	0	4
Metabolism	Carbohydrate Metabolism	00520 Amino sugar and nucleotide sugar metabolism [PATH:ko00520]	polyphosphate glucokinase [EC:2.7.1.63]	K00886	0	1	0	0	0
Metabolism	Carbohydrate Metabolism	00520 Amino sugar and nucleotide sugar metabolism [PATH:ko00520]	UDPglucose 6-dehydrogenase [EC:1.1.1.22]	K00012	0	0	0	5	3
Metabolism	Carbohydrate Metabolism	00520 Amino sugar and nucleotide sugar metabolism [PATH:ko00520]	UDPglucose--hexose-1-phosphate uridylyltransferase	K00965	0	0	0	3	2
Metabolism	Carbohydrate Metabolism	00520 Amino sugar and nucleotide sugar metabolism [PATH:ko00520]	UDP-glucuronate 4-epimerase [EC:5.1.3.6]	K08679	0	1	0	0	0
Metabolism	Carbohydrate Metabolism	00520 Amino sugar and nucleotide sugar metabolism [PATH:ko00520]	UDP-N-acetyl-D-galactosamine dehydrogenase [EC:1.1.1.-]	K02474	0	0	2	0	0

Level 1	Level 2	Level 3	Function	KEGG ID	Sequence Reads				
					Day17	Day24	Day31	Day38	Bulk Soil
Metabolism	Carbohydrate Metabolism	00520 Amino sugar and nucleotide sugar metabolism [PATH:ko00520]	UDP-N-acetyl-D-mannosaminuronic acid dehydrogenase	K02472	0	0	2	0	0
Metabolism	Carbohydrate Metabolism	00520 Amino sugar and nucleotide sugar metabolism [PATH:ko00520]	UDP-N-acetylglucosamine 1-carboxyvinyltransferase	K00790	4	7	5	0	0
Metabolism	Carbohydrate Metabolism	00520 Amino sugar and nucleotide sugar metabolism [PATH:ko00520]	UDP-N-acetylglucosamine 2-epimerase [EC:5.1.3.14]	K01791	0	0	0	4	0
Metabolism	Carbohydrate Metabolism	00520 Amino sugar and nucleotide sugar metabolism [PATH:ko00520]	UDP-N-acetylglucosamine 4-epimerase [EC:5.1.3.7]	K02473	0	1	0	0	0
Metabolism	Carbohydrate Metabolism	00520 Amino sugar and nucleotide sugar metabolism [PATH:ko00520]	undecaprenyl-phosphate 4-deoxy-4-formamido-L-arabinose	K10012	0	0	0	0	3
Metabolism	Carbohydrate Metabolism	00520 Amino sugar and nucleotide sugar metabolism [PATH:ko00520]	xylan 1,4-beta-xylosidase [EC:3.2.1.37]	K01198	0	0	1	0	0
Metabolism	Carbohydrate Metabolism	00562 Inositol phosphate metabolism [PATH:ko00562]	3D-(3,5/4)-trihydroxycyclohexane-1,2-dione hydrolase	K03336	0	0	0	0	2
Metabolism	Carbohydrate Metabolism	00562 Inositol phosphate metabolism [PATH:ko00562]	inositol oxygenase [EC:1.13.99.1]	K00469	0	1	0	0	0
Metabolism	Carbohydrate Metabolism	00620 Pyruvate metabolism [PATH:ko00620]	acetyl-CoA hydrolase [EC:3.1.2.1]	K01067	0	0	2	0	0
Metabolism	Carbohydrate Metabolism	00620 Pyruvate metabolism [PATH:ko00620]	D-lactate dehydrogenase (cytochrome) [EC:1.1.2.4]	K00102	4	2	0	6	2

Level 1	Level 2	Level 3	Function	KEGG ID	Sequence Reads				
					Day17	Day24	Day31	Day38	Bulk Soil
Metabolism	Carbohydrate Metabolism	00620 Pyruvate metabolism [PATH:ko00620]	hydroxyacylglutathione hydrolase [EC:3.1.2.6]	K01069	0	0	1	0	0
Metabolism	Carbohydrate Metabolism	00620 Pyruvate metabolism [PATH:ko00620]	L-lactate dehydrogenase (cytochrome) [EC:1.1.2.3]	K00101	0	0	2	1	3
Metabolism	Carbohydrate Metabolism	00620 Pyruvate metabolism [PATH:ko00620]	malate dehydrogenase (quinone) [EC:1.1.5.4]	K00116	0	0	0	1	0
Metabolism	Carbohydrate Metabolism	00620 Pyruvate metabolism [PATH:ko00620]	pyruvate carboxylase [EC:6.4.1.1]	K01958	0	0	1	2	1
Metabolism	Carbohydrate Metabolism	00620 Pyruvate metabolism [PATH:ko00620]	pyruvate carboxylase subunit A [EC:6.4.1.1]	K01959	0	0	0	1	0
Metabolism	Carbohydrate Metabolism	00620 Pyruvate metabolism [PATH:ko00620]	pyruvate dehydrogenase (quinone) [EC:1.2.5.1]	K00156	1	1	4	0	2
Metabolism	Carbohydrate Metabolism	00620 Pyruvate metabolism [PATH:ko00620]	pyruvate dehydrogenase E2 component (dihydrolipoamide	K00627	10	0	0	4	0
Metabolism	Carbohydrate Metabolism	00630 Glyoxylate and dicarboxylate metabolism [PATH:ko00630]	(2S)-methylsuccinyl-CoA dehydrogenase	K14448	0	0	0	2	0
Metabolism	Carbohydrate Metabolism	00630 Glyoxylate and dicarboxylate metabolism [PATH:ko00630]	2-hydroxy-3-oxopropionate reductase [EC:1.1.1.60]	K00042	0	2	0	0	0
Metabolism	Carbohydrate Metabolism	00630 Glyoxylate and dicarboxylate metabolism [PATH:ko00630]	citrate synthase [EC:2.3.3.1]	K01647	0	10	5	9	0
Metabolism	Carbohydrate Metabolism	00630 Glyoxylate and dicarboxylate metabolism [PATH:ko00630]	crotonyl-CoA carboxylase/reductase	K14446	1	4	1	0	1
Metabolism	Carbohydrate Metabolism	00630 Glyoxylate and dicarboxylate metabolism	ethylmalonyl-CoA mutase	K14447	0	1	3	2	3

Level 1	Level 2	Level 3	Function	KEGG ID	Sequence Reads				
					Day17	Day24	Day31	Day38	Bulk Soil
		[PATH:ko00630]							
Metabolism	Carbohydrate Metabolism	00630 Glyoxylate and dicarboxylate metabolism [PATH:ko00630]	glycolate oxidase [EC:1.1.3.15]	K00104	0	5	3	0	5
Metabolism	Carbohydrate Metabolism	00630 Glyoxylate and dicarboxylate metabolism [PATH:ko00630]	glycolate oxidase FAD binding subunit	K11472	0	3	0	0	0
Metabolism	Carbohydrate Metabolism	00630 Glyoxylate and dicarboxylate metabolism [PATH:ko00630]	glycolate oxidase iron-sulfur subunit	K11473	0	3	0	0	0
Metabolism	Carbohydrate Metabolism	00630 Glyoxylate and dicarboxylate metabolism [PATH:ko00630]	glyoxylate reductase [EC:1.1.1.26]	K00015	0	0	0	1	0
Metabolism	Carbohydrate Metabolism	00630 Glyoxylate and dicarboxylate metabolism [PATH:ko00630]	L(+)-tartrate dehydratase beta subunit [EC:4.2.1.32]	K03780	0	0	0	2	0
Metabolism	Carbohydrate Metabolism	00630 Glyoxylate and dicarboxylate metabolism [PATH:ko00630]	malate synthase [EC:2.3.3.9]	K01638	0	2	2	2	0
Metabolism	Carbohydrate Metabolism	00630 Glyoxylate and dicarboxylate metabolism [PATH:ko00630]	oxalyl-CoA decarboxylase [EC:4.1.1.8]	K01577	1	1	0	0	0
Metabolism	Carbohydrate Metabolism	00630 Glyoxylate and dicarboxylate metabolism [PATH:ko00630]	phosphoglycolate phosphatase [EC:3.1.3.18]	K01091	0	0	0	1	2
Metabolism	Carbohydrate Metabolism	00630 Glyoxylate and dicarboxylate metabolism [PATH:ko00630]	ribulose-bisphosphate carboxylase large chain [EC:4.1.1.39]	K01601	7	2	4	0	0

Level 1	Level 2	Level 3	Function	KEGG ID	Sequence Reads				
					Day17	Day24	Day31	Day38	Bulk Soil
Metabolism	Carbohydrate Metabolism	00630 Glyoxylate and dicarboxylate metabolism [PATH:ko00630]	tartronate-semialdehyde synthase [EC:4.1.1.47]	K01608	1	1	1	0	0
Metabolism	Carbohydrate Metabolism	00640 Propanoate metabolism [PATH:ko00640]	1-aminocyclopropane-1-carboxylate deaminase [EC:3.5.99.7]	K01505	0	2	0	0	0
Metabolism	Carbohydrate Metabolism	00640 Propanoate metabolism [PATH:ko00640]	2-methylcitrate dehydratase [EC:4.2.1.79]	K01720	1	0	0	0	0
Metabolism	Carbohydrate Metabolism	00640 Propanoate metabolism [PATH:ko00640]	2-methylcitrate synthase [EC:2.3.3.5]	K01659	1	0	0	0	0
Metabolism	Carbohydrate Metabolism	00640 Propanoate metabolism [PATH:ko00640]	methylmalonyl-CoA decarboxylase [EC:4.1.1.41]	K11264	0	1	0	0	0
Metabolism	Carbohydrate Metabolism	00640 Propanoate metabolism [PATH:ko00640]	methylmalonyl-CoA decarboxylase beta chain [EC:4.1.1.41]	K01605	1	0	0	0	0
Metabolism	Carbohydrate Metabolism	00640 Propanoate metabolism [PATH:ko00640]	propionyl-CoA synthetase [EC:6.2.1.17]	K01908	0	0	1	3	1
Metabolism	Carbohydrate Metabolism	00650 Butanoate metabolism [PATH:ko00650]	acetoacetyl-CoA synthetase [EC:6.2.1.16]	K01907	0	0	0	1	0
Metabolism	Carbohydrate Metabolism	00650 Butanoate metabolism [PATH:ko00650]	formate C-acetyltransferase [EC:2.3.1.54]	K00656	2	0	0	0	1
Metabolism	Carbohydrate Metabolism	00650 Butanoate metabolism [PATH:ko00650]	hydroxybutyrate-dimer hydrolase [EC:3.1.1.22]	K07518	0	0	1	0	0
Metabolism	Carbohydrate Metabolism	00650 Butanoate metabolism [PATH:ko00650]	medium-chain acyl-CoA synthetase [EC:6.2.1.2]	K01896	0	1	0	0	0
Metabolism	Carbohydrate Metabolism	00650 Butanoate metabolism [PATH:ko00650]	poly(3-hydroxybutyrate) depolymerase [EC:3.1.1.75]	K05973	0	0	1	3	0

Level 1	Level 2	Level 3	Function	KEGG ID	Sequence Reads				
					Day17	Day24	Day31	Day38	Bulk Soil
Metabolism	Carbohydrate Metabolism	00650 Butanoate metabolism [PATH:ko00650]	polyhydroxyalkanoate synthase [EC:2.3.1.-]	K03821	1	3	1	0	0
Metabolism	Carbohydrate Metabolism	00650 Butanoate metabolism [PATH:ko00650]	tartrate dehydrogenase/decarboxylase / D-malate	K07246	1	4	1	1	0
Metabolism	Carbohydrate Metabolism	00650 Butanoate metabolism [PATH:ko00650]	trans-2-enoyl-CoA reductase (NAD+) [EC:1.3.1.44]	K10783	0	0	0	1	0
Metabolism	Carbohydrate Metabolism	00660 C5-Branched dibasic acid metabolism [PATH:ko00660]	methylaspartate mutase [EC:5.4.99.1]	K01846	1	0	0	0	0
Metabolism	Energy Metabolism	00190 Oxidative phosphorylation [PATH:ko00190]	cb-type cytochrome c oxidase subunit I [EC:1.9.3.1]	K00404	0	0	0	1	2
Metabolism	Energy Metabolism	00190 Oxidative phosphorylation [PATH:ko00190]	cb-type cytochrome c oxidase subunit II [EC:1.9.3.1]	K00405	0	0	0	2	0
Metabolism	Energy Metabolism	00190 Oxidative phosphorylation [PATH:ko00190]	cb-type cytochrome c oxidase subunit III [EC:1.9.3.1]	K00406	1	0	0	0	0
Metabolism	Energy Metabolism	00190 Oxidative phosphorylation [PATH:ko00190]	cytochrome bd-I oxidase subunit I [EC:1.10.3.-]	K00425	2	0	0	0	4
Metabolism	Energy Metabolism	00190 Oxidative phosphorylation [PATH:ko00190]	cytochrome bd-I oxidase subunit II [EC:1.10.3.-]	K00426	0	0	0	2	0
Metabolism	Energy Metabolism	00190 Oxidative phosphorylation [PATH:ko00190]	cytochrome c oxidase subunit I [EC:1.9.3.1]	K02274	5	13	8	12	0
Metabolism	Energy Metabolism	00190 Oxidative phosphorylation [PATH:ko00190]	cytochrome c oxidase subunit III [EC:1.9.3.1]	K02276	0	0	0	5	0
Metabolism	Energy Metabolism	00190 Oxidative phosphorylation [PATH:ko00190]	cytochrome c oxidase subunit XI assembly protein	K02258	1	0	0	0	0

Level 1	Level 2	Level 3	Function	KEGG ID	Sequence Reads				
					Day17	Day24	Day31	Day38	Bulk Soil
Metabolism	Energy Metabolism	00190 Oxidative phosphorylation [PATH:ko00190]	cytochrome o ubiquinol oxidase subunit I [EC:1.10.3.-]	K02298	0	0	2	0	0
Metabolism	Energy Metabolism	00190 Oxidative phosphorylation [PATH:ko00190]	H+-transporting ATPase [EC:3.6.3.6]	K01535	2	1	1	0	0
Metabolism	Energy Metabolism	00190 Oxidative phosphorylation [PATH:ko00190]	inorganic pyrophosphatase [EC:3.6.1.1]	K01507	18	5	15	0	10
Metabolism	Energy Metabolism	00190 Oxidative phosphorylation [PATH:ko00190]	menaquinol-cytochrome c reductase cytochrome b/c subunit	K03888	0	0	1	0	0
Metabolism	Energy Metabolism	00190 Oxidative phosphorylation [PATH:ko00190]	NADH dehydrogenase (quinone) [EC:1.6.99.5]	K05903	0	0	1	0	0
Metabolism	Energy Metabolism	00190 Oxidative phosphorylation [PATH:ko00190]	NADH dehydrogenase I subunit 2 [EC:1.6.5.3]	K05573	1	0	0	0	0
Metabolism	Energy Metabolism	00190 Oxidative phosphorylation [PATH:ko00190]	NADH dehydrogenase I subunit 4L [EC:1.6.5.3]	K05576	0	0	0	1	0
Metabolism	Energy Metabolism	00190 Oxidative phosphorylation [PATH:ko00190]	NADH dehydrogenase I subunit 5 [EC:1.6.5.3]	K05577	3	0	2	0	0
Metabolism	Energy Metabolism	00190 Oxidative phosphorylation [PATH:ko00190]	NADH dehydrogenase I subunit 6 [EC:1.6.5.3]	K05578	1	0	0	0	0
Metabolism	Energy Metabolism	00190 Oxidative phosphorylation [PATH:ko00190]	NADH dehydrogenase I subunit A [EC:1.6.5.3]	K00330	0	0	0	1	1
Metabolism	Energy Metabolism	00190 Oxidative phosphorylation [PATH:ko00190]	NADH dehydrogenase I subunit B [EC:1.6.5.3]	K00331	3	2	2	2	1
Metabolism	Energy Metabolism	00190 Oxidative phosphorylation [PATH:ko00190]	NADH dehydrogenase I subunit C [EC:1.6.5.3]	K00332	1	0	0	0	3
Metabolism	Energy Metabolism	00190 Oxidative phosphorylation [PATH:ko00190]	NADH dehydrogenase I subunit C/D [EC:1.6.5.3]	K13378	0	1	0	0	0

Level 1	Level 2	Level 3	Function	KEGG ID	Sequence Reads				
					Day17	Day24	Day31	Day38	Bulk Soil
Metabolism	Energy Metabolism	00190 Oxidative phosphorylation [PATH:ko00190]	NADH dehydrogenase I subunit D [EC:1.6.5.3]	K00333	0	4	0	3	3
Metabolism	Energy Metabolism	00190 Oxidative phosphorylation [PATH:ko00190]	NADH dehydrogenase I subunit E [EC:1.6.5.3]	K00334	1	0	0	0	2
Metabolism	Energy Metabolism	00190 Oxidative phosphorylation [PATH:ko00190]	NADH dehydrogenase I subunit F [EC:1.6.5.3]	K00335	4	7	3	0	0
Metabolism	Energy Metabolism	00190 Oxidative phosphorylation [PATH:ko00190]	NADH dehydrogenase I subunit G [EC:1.6.5.3]	K00336	3	3	0	0	0
Metabolism	Energy Metabolism	00190 Oxidative phosphorylation [PATH:ko00190]	NADH dehydrogenase I subunit H [EC:1.6.5.3]	K00337	2	2	4	0	0
Metabolism	Energy Metabolism	00190 Oxidative phosphorylation [PATH:ko00190]	NADH dehydrogenase I subunit I [EC:1.6.5.3]	K00338	0	2	1	0	1
Metabolism	Energy Metabolism	00190 Oxidative phosphorylation [PATH:ko00190]	NADH dehydrogenase I subunit I [EC:1.6.5.3]	K05580	1	0	0	0	0
Metabolism	Energy Metabolism	00190 Oxidative phosphorylation [PATH:ko00190]	NADH dehydrogenase I subunit J [EC:1.6.5.3]	K00339	4	2	0	0	0
Metabolism	Energy Metabolism	00190 Oxidative phosphorylation [PATH:ko00190]	NADH dehydrogenase I subunit K [EC:1.6.5.3]	K00340	1	1	2	3	1
Metabolism	Energy Metabolism	00190 Oxidative phosphorylation [PATH:ko00190]	NADH dehydrogenase I subunit L [EC:1.6.5.3]	K00341	5	9	0	5	0
Metabolism	Energy Metabolism	00190 Oxidative phosphorylation [PATH:ko00190]	NADH dehydrogenase I subunit M [EC:1.6.5.3]	K00342	2	0	1	8	5
Metabolism	Energy Metabolism	00190 Oxidative phosphorylation [PATH:ko00190]	NADH dehydrogenase I subunit N [EC:1.6.5.3]	K00343	2	0	0	0	0
Metabolism	Energy Metabolism	00190 Oxidative phosphorylation [PATH:ko00190]	ubiquinol-cytochrome c reductase cytochrome b subunit	K03891	0	0	0	0	2

Level 1	Level 2	Level 3	Function	KEGG ID	Sequence Reads				
					Day17	Day24	Day31	Day38	Bulk Soil
Metabolism	Energy Metabolism	00195 Photosynthesis [PATH:ko00195]	apocytochrome f	K02634	0	0	2	1	0
Metabolism	Energy Metabolism	00195 Photosynthesis [PATH:ko00195]	cytochrome b6	K02635	5	0	6	0	0
Metabolism	Energy Metabolism	00195 Photosynthesis [PATH:ko00195]	cytochrome b6-f complex iron-sulfur subunit [EC:1.10.99.1]	K02636	1	0	0	0	0
Metabolism	Energy Metabolism	00195 Photosynthesis [PATH:ko00195]	cytochrome b6-f complex subunit 4	K02637	6	1	2	0	0
Metabolism	Energy Metabolism	00195 Photosynthesis [PATH:ko00195]	ferredoxin	K02639	1	2	1	0	0
Metabolism	Energy Metabolism	00195 Photosynthesis [PATH:ko00195]	ferredoxin--NADP+ reductase [EC:1.18.1.2]	K02641	2	0	4	0	0
Metabolism	Energy Metabolism	00195 Photosynthesis [PATH:ko00195]	F-type H+-transporting ATPase subunit a [EC:3.6.3.14]	K02108	21	3	5	2	1
Metabolism	Energy Metabolism	00195 Photosynthesis [PATH:ko00195]	F-type H+-transporting ATPase subunit alpha [EC:3.6.3.14]	K02111	18	5	9	5	6
Metabolism	Energy Metabolism	00195 Photosynthesis [PATH:ko00195]	F-type H+-transporting ATPase subunit b [EC:3.6.3.14]	K02109	6	0	0	0	0
Metabolism	Energy Metabolism	00195 Photosynthesis [PATH:ko00195]	F-type H+-transporting ATPase subunit beta [EC:3.6.3.14]	K02112	9	6	4	5	4
Metabolism	Energy Metabolism	00195 Photosynthesis [PATH:ko00195]	F-type H+-transporting ATPase subunit c [EC:3.6.3.14]	K02110	5	1	0	4	0
Metabolism	Energy Metabolism	00195 Photosynthesis [PATH:ko00195]	F-type H+-transporting ATPase subunit delta [EC:3.6.3.14]	K02113	0	1	1	0	0
Metabolism	Energy Metabolism	00195 Photosynthesis [PATH:ko00195]	F-type H+-transporting ATPase subunit gamma [EC:3.6.3.14]	K02115	6	0	4	0	0
Metabolism	Energy Metabolism	00195 Photosynthesis	photosystem I P700 chlorophyll a apoprotein A1	K02689	45	15	20	19	0

Level 1	Level 2	Level 3	Function	KEGG ID	Sequence Reads				
					Day17	Day24	Day31	Day38	Bulk Soil
		[PATH:ko00195]							
Metabolism	Energy Metabolism	00195 Photosynthesis [PATH:ko00195]	photosystem I P700 chlorophyll a apoprotein A2	K02690	84	9	29	32	0
Metabolism	Energy Metabolism	00195 Photosynthesis [PATH:ko00195]	photosystem I subunit II	K02692	0	0	1	0	0
Metabolism	Energy Metabolism	00195 Photosynthesis [PATH:ko00195]	photosystem I subunit IV	K02693	2	0	0	2	0
Metabolism	Energy Metabolism	00195 Photosynthesis [PATH:ko00195]	photosystem I subunit IX	K02697	0	0	1	1	0
Metabolism	Energy Metabolism	00195 Photosynthesis [PATH:ko00195]	photosystem I subunit VII	K02691	1	0	1	0	0
Metabolism	Energy Metabolism	00195 Photosynthesis [PATH:ko00195]	photosystem I subunit XI	K02699	0	0	2	1	0
Metabolism	Energy Metabolism	00195 Photosynthesis [PATH:ko00195]	photosystem I subunit XII	K02700	1	0	0	0	0
Metabolism	Energy Metabolism	00195 Photosynthesis [PATH:ko00195]	photosystem II 13kDa protein	K08903	1	0	0	0	0
Metabolism	Energy Metabolism	00195 Photosynthesis [PATH:ko00195]	photosystem II CP43 chlorophyll apoprotein	K02705	4	1	4	5	0
Metabolism	Energy Metabolism	00195 Photosynthesis [PATH:ko00195]	photosystem II CP47 chlorophyll apoprotein	K02704	9	2	4	3	0
Metabolism	Energy Metabolism	00195 Photosynthesis [PATH:ko00195]	photosystem II cytochrome b559 subunit alpha	K02707	3	0	1	1	0
Metabolism	Energy Metabolism	00195 Photosynthesis [PATH:ko00195]	photosystem II cytochrome b559 subunit beta	K02708	0	0	1	1	0
Metabolism	Energy Metabolism	00195 Photosynthesis [PATH:ko00195]	photosystem II P680 reaction center D2 protein	K02706	6	5	9	7	0

Level 1	Level 2	Level 3	Function	KEGG ID	Sequence Reads				
					Day17	Day24	Day31	Day38	Bulk Soil
Metabolism	Energy Metabolism	00195 Photosynthesis [PATH:ko00195]	photosystem II PsbH protein	K02709	2	0	0	2	0
Metabolism	Energy Metabolism	00195 Photosynthesis [PATH:ko00195]	photosystem II PsbI protein	K02710	1	0	0	0	0
Metabolism	Energy Metabolism	00195 Photosynthesis [PATH:ko00195]	photosystem II PsbJ protein	K02711	0	0	0	1	0
Metabolism	Energy Metabolism	00195 Photosynthesis [PATH:ko00195]	photosystem II PsbK protein	K02712	0	0	0	1	0
Metabolism	Energy Metabolism	00195 Photosynthesis [PATH:ko00195]	photosystem II PsbL protein	K02713	0	0	1	1	0
Metabolism	Energy Metabolism	00196 Photosynthesis - antenna proteins [PATH:ko00196]	allophycocyanin beta subunit	K02093	8	1	2	7	0
Metabolism	Energy Metabolism	00196 Photosynthesis - antenna proteins [PATH:ko00196]	allophycocyanin-B	K02095	0	0	0	1	0
Metabolism	Energy Metabolism	00196 Photosynthesis - antenna proteins [PATH:ko00196]	bilin biosynthesis protein	K05385	0	0	1	0	0
Metabolism	Energy Metabolism	00196 Photosynthesis - antenna proteins [PATH:ko00196]	light-harvesting complex I chlorophyll a/b binding protein 2	K08908	0	0	0	1	0
Metabolism	Energy Metabolism	00196 Photosynthesis - antenna proteins [PATH:ko00196]	phycobilisome core component	K02097	0	0	0	1	0
Metabolism	Energy Metabolism	00196 Photosynthesis - antenna proteins [PATH:ko00196]	phycobilisome core linker protein	K02094	3	1	0	0	0

Level 1	Level 2	Level 3	Function	KEGG ID	Sequence Reads				
					Day17	Day24	Day31	Day38	Bulk Soil
Metabolism	Energy Metabolism	00196 Photosynthesis - antenna proteins [PATH:ko00196]	phycobilisome core-membrane linker protein	K02096	8	1	1	3	0
Metabolism	Energy Metabolism	00196 Photosynthesis - antenna proteins [PATH:ko00196]	phycobilisome rod-core linker protein	K02290	1	0	0	1	0
Metabolism	Energy Metabolism	00196 Photosynthesis - antenna proteins [PATH:ko00196]	phycocyanin alpha chain	K02284	5	2	10	7	0
Metabolism	Energy Metabolism	00196 Photosynthesis - antenna proteins [PATH:ko00196]	phycocyanin beta chain	K02285	35	3	11	6	0
Metabolism	Energy Metabolism	00196 Photosynthesis - antenna proteins [PATH:ko00196]	phycocyanin-associated rod linker protein	K02286	5	0	2	1	0
Metabolism	Energy Metabolism	00196 Photosynthesis - antenna proteins [PATH:ko00196]	phycocyanin-associated, rod	K02287	0	1	0	0	0
Metabolism	Energy Metabolism	00196 Photosynthesis - antenna proteins [PATH:ko00196]	phycocyanobilin lyase alpha subunit [EC:4.-.-.-]	K02288	0	0	2	1	0
Metabolism	Energy Metabolism	00196 Photosynthesis - antenna proteins [PATH:ko00196]	phycoerythrin alpha chain	K05376	0	0	1	3	0
Metabolism	Energy Metabolism	00196 Photosynthesis - antenna proteins [PATH:ko00196]	phycoerythrin beta chain	K05377	4	0	0	2	0
Metabolism	Energy Metabolism	00196 Photosynthesis - antenna proteins [PATH:ko00196]	phycoerythrin-associated linker protein	K05378	0	1	2	0	0

Level 1	Level 2	Level 3	Function	KEGG ID	Sequence Reads				
					Day17	Day24	Day31	Day38	Bulk Soil
Metabolism	Energy Metabolism	00680 Methane metabolism [PATH:ko00680]	6-phosphofructokinase [EC:2.7.1.11]	K00850	2	3	0	0	4
Metabolism	Energy Metabolism	00680 Methane metabolism [PATH:ko00680]	acetyl-CoA synthetase [EC:6.2.1.1]	K01895	13	5	0	6	0
Metabolism	Energy Metabolism	00680 Methane metabolism [PATH:ko00680]	coenzyme F420 hydrogenase beta subunit [EC:1.12.98.1]	K00441	1	0	1	0	0
Metabolism	Energy Metabolism	00680 Methane metabolism [PATH:ko00680]	coenzyme F420-dependent	K00320	0	0	0	0	1
Metabolism	Energy Metabolism	00680 Methane metabolism [PATH:ko00680]	energy-converting hydrogenase B subunit Q	K06862	0	0	1	0	0
Metabolism	Energy Metabolism	00680 Methane metabolism [PATH:ko00680]	formaldehyde-activating enzyme [EC:4.3.-.-]	K10713	3	1	2	0	0
Metabolism	Energy Metabolism	00680 Methane metabolism [PATH:ko00680]	formate dehydrogenase, alpha subunit [EC:1.2.1.2]	K00123	0	11	5	0	0
Metabolism	Energy Metabolism	00680 Methane metabolism [PATH:ko00680]	formate dehydrogenase, beta subunit [EC:1.2.1.2]	K00124	0	0	0	1	4
Metabolism	Energy Metabolism	00680 Methane metabolism [PATH:ko00680]	formate dehydrogenase, gamma subunit [EC:1.2.1.2]	K00127	0	1	0	0	1
Metabolism	Energy Metabolism	00680 Methane metabolism [PATH:ko00680]	fructose-bisphosphate aldolase, class II [EC:4.1.2.13]	K01624	1	0	11	4	3
Metabolism	Energy Metabolism	00680 Methane metabolism [PATH:ko00680]	heterodisulfide reductase subunit B [EC:1.8.98.1]	K03389	0	0	1	0	0
Metabolism	Energy Metabolism	00680 Methane metabolism [PATH:ko00680]	hydrogen dehydrogenase [EC:1.12.1.2]	K00436	0	0	0	0	1
Metabolism	Energy Metabolism	00680 Methane metabolism [PATH:ko00680]	malate dehydrogenase [EC:1.1.1.37]	K00024	0	1	0	0	0

Level 1	Level 2	Level 3	Function	KEGG ID	Sequence Reads				
					Day17	Day24	Day31	Day38	Bulk Soil
Metabolism	Energy Metabolism	00680 Methane metabolism [PATH:ko00680]	malate-CoA ligase subunit alpha [EC:6.2.1.9]	K08692	0	1	0	0	0
Metabolism	Energy Metabolism	00680 Methane metabolism [PATH:ko00680]	malate-CoA ligase subunit beta [EC:6.2.1.9]	K14067	0	0	1	0	0
Metabolism	Energy Metabolism	00680 Methane metabolism [PATH:ko00680]	malyl-CoA lyase [EC:4.1.3.24]	K08691	0	0	0	2	1
Metabolism	Energy Metabolism	00680 Methane metabolism [PATH:ko00680]	Na+:H+ antiporter, NhaA family	K03313	0	0	0	0	3
Metabolism	Energy Metabolism	00680 Methane metabolism [PATH:ko00680]	phosphoenolpyruvate carboxylase [EC:4.1.1.31]	K01595	3	6	0	4	7
Metabolism	Energy Metabolism	00680 Methane metabolism [PATH:ko00680]	phosphoglycerate mutase [EC:5.4.2.1]	K01834	6	5	4	0	0
Metabolism	Energy Metabolism	00680 Methane metabolism [PATH:ko00680]	phosphoketolase [EC:4.1.2.9]	K01621	0	0	0	0	1
Metabolism	Energy Metabolism	00680 Methane metabolism [PATH:ko00680]	pyruvate, water dikinase [EC:2.7.9.2]	K01007	4	3	3	7	4
Metabolism	Energy Metabolism	00680 Methane metabolism [PATH:ko00680]	S-formylglutathione hydrolase [EC:3.1.2.12]	K01070	0	0	0	1	0
Metabolism	Energy Metabolism	00680 Methane metabolism [PATH:ko00680]	V-type H+-transporting ATPase subunit I [EC:3.6.3.14]	K02123	0	0	0	1	0
Metabolism	Energy Metabolism	00710 Carbon fixation in photosynthetic organisms [PATH:ko00710]	fructose-1,6-bisphosphatase II /	K11532	1	0	1	0	0
Metabolism	Energy Metabolism	00710 Carbon fixation in photosynthetic organisms [PATH:ko00710]	fructose-6-phosphate phosphoketolase [EC:4.1.2.22]	K01632	0	2	0	0	4

Level 1	Level 2	Level 3	Function	KEGG ID	Sequence Reads				
					Day17	Day24	Day31	Day38	Bulk Soil
Metabolism	Energy Metabolism	00710 Carbon fixation in photosynthetic organisms [PATH:ko00710]	fructose-bisphosphate aldolase, class I [EC:4.1.2.13]	K01623	0	2	0	1	0
Metabolism	Energy Metabolism	00710 Carbon fixation in photosynthetic organisms [PATH:ko00710]	glyceraldehyde-3-phosphate dehydrogenase (NAD(P))	K00150	0	0	1	0	0
Metabolism	Energy Metabolism	00710 Carbon fixation in photosynthetic organisms [PATH:ko00710]	glyceraldehyde-3-phosphate dehydrogenase (NADP+)	K05298	0	0	0	1	0
Metabolism	Energy Metabolism	00710 Carbon fixation in photosynthetic organisms [PATH:ko00710]	malate dehydrogenase (oxaloacetate-decarboxylating)(NADP+)	K00029	0	2	5	1	2
Metabolism	Energy Metabolism	00710 Carbon fixation in photosynthetic organisms [PATH:ko00710]	malate dehydrogenase [EC:1.1.1.37]	K00026	0	1	1	0	0
Metabolism	Energy Metabolism	00710 Carbon fixation in photosynthetic organisms [PATH:ko00710]	phosphoenolpyruvate carboxykinase (ATP) [EC:4.1.1.49]	K01610	0	2	0	0	0
Metabolism	Energy Metabolism	00710 Carbon fixation in photosynthetic organisms [PATH:ko00710]	phosphoglycerate kinase [EC:2.7.2.3]	K00927	4	3	4	0	8
Metabolism	Energy Metabolism	00710 Carbon fixation in photosynthetic organisms [PATH:ko00710]	phosphoribulokinase [EC:2.7.1.19]	K00855	1	0	2	1	1

Level 1	Level 2	Level 3	Function	KEGG ID	Sequence Reads				
					Day17	Day24	Day31	Day38	Bulk Soil
Metabolism	Energy Metabolism	00710 Carbon fixation in photosynthetic organisms [PATH:ko00710]	pyruvate,orthophosphate dikinase [EC:2.7.9.1]	K01006	6	0	9	7	13
Metabolism	Energy Metabolism	00710 Carbon fixation in photosynthetic organisms [PATH:ko00710]	ribulose-bisphosphate carboxylase small chain [EC:4.1.1.39]	K01602	0	0	1	0	0
Metabolism	Energy Metabolism	00710 Carbon fixation in photosynthetic organisms [PATH:ko00710]	ribulose-phosphate 3-epimerase [EC:5.1.3.1]	K01783	0	1	0	2	0
Metabolism	Energy Metabolism	00710 Carbon fixation in photosynthetic organisms [PATH:ko00710]	triosephosphate isomerase (TIM) [EC:5.3.1.1]	K01803	7	3	0	0	0
Metabolism	Energy Metabolism	00720 Carbon fixation in autotrophic prokaryotes [PATH:ko00720]	2-oxoglutarate ferredoxin oxidoreductase subunit alpha	K00174	0	0	3	2	0
Metabolism	Energy Metabolism	00720 Carbon fixation in autotrophic prokaryotes [PATH:ko00720]	2-oxoglutarate ferredoxin oxidoreductase subunit beta	K00175	0	3	0	3	0
Metabolism	Energy Metabolism	00720 Carbon fixation in autotrophic prokaryotes [PATH:ko00720]	2-oxoglutarate ferredoxin oxidoreductase subunit gamma	K00177	2	0	0	0	0
Metabolism	Energy Metabolism	00720 Carbon fixation in autotrophic prokaryotes [PATH:ko00720]	aconitate hydratase 1 [EC:4.2.1.3]	K01681	5	2	7	8	8

Level 1	Level 2	Level 3	Function	KEGG ID	Sequence Reads				
					Day17	Day24	Day31	Day38	Bulk Soil
Metabolism	Energy Metabolism	00720 Carbon fixation in autotrophic prokaryotes [PATH:ko00720]	aconitate hydratase 2 [EC:4.2.1.3]	K01682	2	0	0	1	0
Metabolism	Energy Metabolism	00720 Carbon fixation in autotrophic prokaryotes [PATH:ko00720]	ATP citrate (pro-S)-lyase [EC:2.3.3.8]	K01648	0	0	1	0	0
Metabolism	Energy Metabolism	00720 Carbon fixation in autotrophic prokaryotes [PATH:ko00720]	fumarate hydratase, class I [EC:4.2.1.2]	K01676	0	4	0	2	3
Metabolism	Energy Metabolism	00720 Carbon fixation in autotrophic prokaryotes [PATH:ko00720]	mesaconyl-C4 CoA hydratase	K09709	0	0	0	1	0
Metabolism	Energy Metabolism	00720 Carbon fixation in autotrophic prokaryotes [PATH:ko00720]	mesaconyl-CoA hydratase	K14449	0	0	2	0	1
Metabolism	Energy Metabolism	00720 Carbon fixation in autotrophic prokaryotes [PATH:ko00720]	succinyl-CoA synthetase alpha subunit [EC:6.2.1.5]	K01902	3	0	0	3	1
Metabolism	Energy Metabolism	00720 Carbon fixation in autotrophic prokaryotes [PATH:ko00720]	succinyl-CoA synthetase beta subunit [EC:6.2.1.5]	K01903	2	0	0	4	0
Metabolism	Energy Metabolism	00910 Nitrogen metabolism [PATH:ko00910]	carbonic anhydrase [EC:4.2.1.1]	K01673	4	3	0	0	3
Metabolism	Energy Metabolism	00910 Nitrogen metabolism [PATH:ko00910]	ferredoxin-nitrate reductase [EC:1.7.7.2]	K00367	1	0	1	1	0

Level 1	Level 2	Level 3	Function	KEGG ID	Sequence Reads				
					Day17	Day24	Day31	Day38	Bulk Soil
Metabolism	Energy Metabolism	00910 Nitrogen metabolism [PATH:ko00910]	ferredoxin-nitrite reductase [EC:1.7.7.1]	K00366	0	1	3	6	0
Metabolism	Energy Metabolism	00910 Nitrogen metabolism [PATH:ko00910]	formamidase [EC:3.5.1.49]	K01455	1	1	0	0	1
Metabolism	Energy Metabolism	00910 Nitrogen metabolism [PATH:ko00910]	glutamate synthase (ferredoxin) [EC:1.4.7.1]	K00284	0	0	5	0	2
Metabolism	Energy Metabolism	00910 Nitrogen metabolism [PATH:ko00910]	hydroxylamine reductase [EC:1.7.99.1]	K05601	5	0	0	0	0
Metabolism	Energy Metabolism	00910 Nitrogen metabolism [PATH:ko00910]	nitrate reductase (NADH) [EC:1.7.1.1]	K00360	0	0	0	0	1
Metabolism	Energy Metabolism	00910 Nitrogen metabolism [PATH:ko00910]	nitrate reductase catalytic subunit [EC:1.7.99.4]	K00372	0	6	4	0	0
Metabolism	Energy Metabolism	00910 Nitrogen metabolism [PATH:ko00910]	nitric-oxide reductase NorQ protein [EC:1.7.99.7]	K04748	2	1	0	0	0
Metabolism	Energy Metabolism	00910 Nitrogen metabolism [PATH:ko00910]	nitrite reductase (NAD(P)H) large subunit [EC:1.7.1.4]	K00362	0	0	3	3	0
Metabolism	Energy Metabolism	00910 Nitrogen metabolism [PATH:ko00910]	nitrite reductase (NO-forming) [EC:1.7.2.1]	K00368	1	0	0	0	1
Metabolism	Energy Metabolism	00910 Nitrogen metabolism [PATH:ko00910]	nitrous-oxide reductase [EC:1.7.99.6]	K00376	0	1	0	0	4
Metabolism	Energy Metabolism	00910 Nitrogen metabolism [PATH:ko00910]	periplasmic nitrate reductase NapA [EC:1.7.99.4]	K02567	0	0	2	1	0
Metabolism	Energy Metabolism	00920 Sulfur metabolism [PATH:ko00920]	serine O-acetyltransferase [EC:2.3.1.30]	K00640	1	0	5	1	0
Metabolism	Energy Metabolism	00920 Sulfur metabolism [PATH:ko00920]	sulfite dehydrogenase [EC:1.8.2.1]	K05301	0	1	0	0	3

Level 1	Level 2	Level 3	Function	KEGG ID	Sequence Reads				
					Day17	Day24	Day31	Day38	Bulk Soil
Metabolism	Energy Metabolism	00920 Sulfur metabolism [PATH:ko00920]	sulfite reductase (NADPH) flavoprotein alpha-component	K00380	0	0	0	0	1
Metabolism	Energy Metabolism	00920 Sulfur metabolism [PATH:ko00920]	sulfite reductase (NADPH) hemoprotein beta-component	K00381	6	0	1	6	0
Metabolism	Glycan Biosynthesis and Metabolism	00510 N-Glycan biosynthesis [PATH:ko00510]	dolichol-phosphate mannosyltransferase [EC:2.4.1.83]	K00721	0	0	1	0	0
Metabolism	Glycan Biosynthesis and Metabolism	00511 Other glycan degradation [PATH:ko00511]	beta-galactosidase [EC:3.2.1.23]	K01190	0	0	0	3	0
Metabolism	Glycan Biosynthesis and Metabolism	00540 Lipopolysaccharide biosynthesis [PATH:ko00540]	2-dehydro-3-deoxyphosphooctonate aldolase (KDO 8-P synthase)	K01627	1	0	0	0	4
Metabolism	Glycan Biosynthesis and Metabolism	00540 Lipopolysaccharide biosynthesis [PATH:ko00540]	3-deoxy-D-manno-octulosonate 8-phosphate phosphatase (KDO	K03270	0	0	0	0	1
Metabolism	Glycan Biosynthesis and Metabolism	00540 Lipopolysaccharide biosynthesis [PATH:ko00540]	3-deoxy-manno-octulosonate cytidylyltransferase (CMP-KDO	K00979	0	0	1	3	0
Metabolism	Glycan Biosynthesis and Metabolism	00540 Lipopolysaccharide biosynthesis [PATH:ko00540]	D-beta-D-heptose 7-phosphate kinase / D-beta-D-heptose	K03272	0	0	0	0	2
Metabolism	Glycan Biosynthesis and Metabolism	00540 Lipopolysaccharide biosynthesis [PATH:ko00540]	tetraacyldisaccharide 4'-kinase [EC:2.7.1.130]	K00912	0	1	0	1	3
Metabolism	Glycan Biosynthesis and Metabolism	00540 Lipopolysaccharide biosynthesis [PATH:ko00540]	UDP-3-O-[3-hydroxymyristoyl] N-acetylglucosamine deacetylase	K02535	0	0	2	0	0

Level 1	Level 2	Level 3	Function	KEGG ID	Sequence Reads				
					Day17	Day24	Day31	Day38	Bulk Soil
Metabolism	Glycan Biosynthesis and Metabolism	00550 Peptidoglycan biosynthesis [PATH:ko00550]	cell division protein FtsI (penicillin-binding protein 3)	K03587	0	0	0	2	0
Metabolism	Glycan Biosynthesis and Metabolism	00550 Peptidoglycan biosynthesis [PATH:ko00550]	D-alanine-D-alanine ligase [EC:6.3.2.4]	K01921	1	0	6	4	0
Metabolism	Glycan Biosynthesis and Metabolism	00550 Peptidoglycan biosynthesis [PATH:ko00550]	D-alanyl-D-alanine carboxypeptidase (penicillin-binding	K07258	0	0	3	0	0
Metabolism	Glycan Biosynthesis and Metabolism	00550 Peptidoglycan biosynthesis [PATH:ko00550]	penicillin-binding protein 1B [EC:2.4.1.129 3.4.-.-]	K05365	0	0	1	0	0
Metabolism	Glycan Biosynthesis and Metabolism	00550 Peptidoglycan biosynthesis [PATH:ko00550]	penicillin-binding protein 1C [EC:2.4.1.-]	K05367	0	0	1	0	1
Metabolism	Glycan Biosynthesis and Metabolism	00550 Peptidoglycan biosynthesis [PATH:ko00550]	penicillin-binding protein 2	K05515	0	2	0	0	0
Metabolism	Glycan Biosynthesis and Metabolism	00550 Peptidoglycan biosynthesis [PATH:ko00550]	peptidoglycan glycosyltransferase [EC:2.4.1.129]	K05364	2	0	0	0	0
Metabolism	Glycan Biosynthesis and Metabolism	00550 Peptidoglycan biosynthesis [PATH:ko00550]	phospho-N-acetylmuramoyl-pentapeptide-transferase	K01000	1	0	0	4	4
Metabolism	Glycan Biosynthesis and Metabolism	00550 Peptidoglycan biosynthesis [PATH:ko00550]	UDP-N-acetylmuramate--alanine ligase [EC:6.3.2.8]	K01924	0	3	0	0	0

Level 1	Level 2	Level 3	Function	KEGG ID	Sequence Reads				
					Day17	Day24	Day31	Day38	Bulk Soil
Metabolism	Glycan Biosynthesis and Metabolism	00550 Peptidoglycan biosynthesis [PATH:ko00550]	UDP-N-acetylmuramoylalanyl-D-glutamate--2,6-diaminopimelate	K01928	0	5	0	0	3
Metabolism	Glycan Biosynthesis and Metabolism	00550 Peptidoglycan biosynthesis [PATH:ko00550]	UDP-N-acetylmuramoylalanyl-D-glutamyl-2,6-diaminopimelate--D	K01929	0	0	0	4	0
Metabolism	Glycan Biosynthesis and Metabolism	00550 Peptidoglycan biosynthesis [PATH:ko00550]	undecaprenyl-diphosphatase [EC:3.6.1.27]	K06153	0	0	3	0	0
Metabolism	Glycan Biosynthesis and Metabolism	00550 Peptidoglycan biosynthesis [PATH:ko00550]	virulence factor	K03980	0	0	1	5	3
Metabolism	Glycan Biosynthesis and Metabolism	00603 Glycosphingolipid biosynthesis - globo series [PATH:ko00603]	alpha-galactosidase [EC:3.2.1.22]	K07406	0	4	0	0	0
Metabolism	Glycan Biosynthesis and Metabolism	00603 Glycosphingolipid biosynthesis - globo series [PATH:ko00603]	alpha-galactosidase [EC:3.2.1.22]	K07407	2	0	0	2	2
Metabolism	Lipid Metabolism	00061 Fatty acid biosynthesis [PATH:ko00061]	[acyl-carrier-protein] S-malonyltransferase [EC:2.3.1.39]	K00645	0	1	0	0	0
Metabolism	Lipid Metabolism	00061 Fatty acid biosynthesis [PATH:ko00061]	3-oxoacyl-[acyl-carrier-protein] synthase I [EC:2.3.1.41]	K00647	0	0	0	2	0
Metabolism	Lipid Metabolism	00061 Fatty acid biosynthesis [PATH:ko00061]	3-oxoacyl-[acyl-carrier-protein] synthase II [EC:2.3.1.179]	K09458	7	9	5	0	0
Metabolism	Lipid Metabolism	00061 Fatty acid biosynthesis [PATH:ko00061]	3-oxoacyl-[acyl-carrier-protein] synthase III [EC:2.3.1.180]	K00648	5	0	0	0	5

Level 1	Level 2	Level 3	Function	KEGG ID	Sequence Reads				
					Day17	Day24	Day31	Day38	Bulk Soil
Metabolism	Lipid Metabolism	00061 Fatty acid biosynthesis [PATH:ko00061]	3R-hydroxymyristoyl ACP dehydrase [EC:4.2.1.-]	K02372	4	0	0	0	0
Metabolism	Lipid Metabolism	00061 Fatty acid biosynthesis [PATH:ko00061]	acetyl-CoA carboxylase carboxyl transferase subunit alpha	K01962	2	2	0	3	0
Metabolism	Lipid Metabolism	00061 Fatty acid biosynthesis [PATH:ko00061]	enoyl-[acyl carrier protein] reductase II [EC:1.3.1.-]	K02371	0	0	0	1	0
Metabolism	Lipid Metabolism	00061 Fatty acid biosynthesis [PATH:ko00061]	enoyl-[acyl-carrier protein] reductase I [EC:1.3.1.9]	K00208	0	4	3	0	4
Metabolism	Lipid Metabolism	00061 Fatty acid biosynthesis [PATH:ko00061]	fatty acid synthase, bacteria type [EC:2.3.1.-]	K11533	0	1	0	0	0
Metabolism	Lipid Metabolism	00071 Fatty acid metabolism [PATH:ko00071]	3-hydroxyacyl-CoA dehydrogenase [EC:1.1.1.35]	K07516	0	3	0	3	0
Metabolism	Lipid Metabolism	00071 Fatty acid metabolism [PATH:ko00071]	acyl-CoA dehydrogenase [EC:1.3.99.-]	K06445	2	0	1	0	0
Metabolism	Lipid Metabolism	00071 Fatty acid metabolism [PATH:ko00071]	very long chain acyl-CoA dehydrogenase [EC:1.3.99.-]	K09479	0	0	1	0	0
Metabolism	Lipid Metabolism	00072 Synthesis and degradation of ketone bodies [PATH:ko00072]	3-hydroxybutyrate dehydrogenase [EC:1.1.1.30]	K00019	0	0	0	2	8
Metabolism	Lipid Metabolism	00100 Steroid biosynthesis [PATH:ko00100]	cholesterol oxidase [EC:1.1.3.6]	K03333	0	2	0	2	2
Metabolism	Lipid Metabolism	00100 Steroid biosynthesis [PATH:ko00100]	squalene-hopene cyclase [EC:5.4.99.17]	K06045	0	0	0	0	4
Metabolism	Lipid Metabolism	00100 Steroid biosynthesis [PATH:ko00100]	sterol-4alpha-carboxylate 3-dehydrogenase (decarboxylating)	K07748	1	0	0	0	0
Metabolism	Lipid Metabolism	00561 Glycerolipid metabolism [PATH:ko00561]	1,2-diacylglycerol 3-beta-galactosyltransferase	K03715	0	0	0	1	0

Level 1	Level 2	Level 3	Function	KEGG ID	Sequence Reads				
					Day17	Day24	Day31	Day38	Bulk Soil
Metabolism	Lipid Metabolism	00561 Glycerolipid metabolism [PATH:ko00561]	diacylglycerol O-acyltransferase [EC:2.3.1.20]	K00635	0	1	0	0	2
Metabolism	Lipid Metabolism	00561 Glycerolipid metabolism [PATH:ko00561]	dihydroxyacetone kinase, N-terminal domain [EC:2.7.1.-]	K05878	0	0	1	0	1
Metabolism	Lipid Metabolism	00561 Glycerolipid metabolism [PATH:ko00561]	UDP-sulfoquinovose synthase [EC:3.13.1.1]	K06118	0	1	1	0	0
Metabolism	Lipid Metabolism	00564 Glycerophospholipid metabolism [PATH:ko00564]	cardiolipin synthase [EC:2.7.8.-]	K06131	0	4	1	0	1
Metabolism	Lipid Metabolism	00564 Glycerophospholipid metabolism [PATH:ko00564]	CDP-diacylglycerol--glycerol-3-phosphate	K00995	1	1	0	0	0
Metabolism	Lipid Metabolism	00564 Glycerophospholipid metabolism [PATH:ko00564]	ethanolamine ammonia-lyase large subunit [EC:4.3.1.7]	K03735	1	0	2	0	2
Metabolism	Lipid Metabolism	00564 Glycerophospholipid metabolism [PATH:ko00564]	ethanolamine kinase [EC:2.7.1.82]	K00894	0	0	0	1	0
Metabolism	Lipid Metabolism	00564 Glycerophospholipid metabolism [PATH:ko00564]	glycerol-3-phosphate acyltransferase PlsX [EC:2.3.1.15]	K03621	2	0	0	0	2
Metabolism	Lipid Metabolism	00564 Glycerophospholipid metabolism [PATH:ko00564]	glycerol-3-phosphate acyltransferase PlsY [EC:2.3.1.15]	K08591	0	1	0	1	0
Metabolism	Lipid Metabolism	00564 Glycerophospholipid metabolism [PATH:ko00564]	glycerol-3-phosphate dehydrogenase (NAD(P)+) [EC:1.1.1.94]	K00057	2	3	0	0	0

Level 1	Level 2	Level 3	Function	KEGG ID	Sequence Reads				
					Day17	Day24	Day31	Day38	Bulk Soil
Metabolism	Lipid Metabolism	00564 Glycerophospholipid metabolism [PATH:ko00564]	glycerol-3-phosphate dehydrogenase [EC:1.1.5.3]	K00111	0	0	4	0	0
Metabolism	Lipid Metabolism	00564 Glycerophospholipid metabolism [PATH:ko00564]	glycerophosphoryl diester phosphodiesterase [EC:3.1.4.46]	K01126	1	2	0	0	0
Metabolism	Lipid Metabolism	00564 Glycerophospholipid metabolism [PATH:ko00564]	lysophospholipase [EC:3.1.1.5]	K01048	0	0	0	1	0
Metabolism	Lipid Metabolism	00564 Glycerophospholipid metabolism [PATH:ko00564]	phosphatidylserine decarboxylase [EC:4.1.1.65]	K01613	0	0	0	1	0
Metabolism	Lipid Metabolism	00565 Ether lipid metabolism [PATH:ko00565]	phospholipase C [EC:3.1.4.3]	K01114	0	5	0	2	0
Metabolism	Lipid Metabolism	00591 Linoleic acid metabolism [PATH:ko00591]	linoleoyl-CoA desaturase [EC:1.14.19.3]	K00508	1	0	2	0	0
Metabolism	Lipid Metabolism	00600 Sphingolipid metabolism [PATH:ko00600]	arylsulfatase [EC:3.1.6.1]	K01130	1	0	6	8	6
Metabolism	Lipid Metabolism	00600 Sphingolipid metabolism [PATH:ko00600]	ceramide glucosyltransferase [EC:2.4.1.80]	K00720	0	0	0	1	0
Metabolism	Lipid Metabolism	01040 Biosynthesis of unsaturated fatty acids [PATH:ko01040]	acyl-[acyl-carrier-protein] desaturase [EC:1.14.19.2]	K03921	1	0	0	0	0
Metabolism	Lipid Metabolism	01040 Biosynthesis of unsaturated fatty acids [PATH:ko01040]	acyl-CoA thioesterase I [EC:3.1.2.- 3.1.1.5]	K10804	1	0	0	0	0
Metabolism	Lipid Metabolism	01040 Biosynthesis of unsaturated fatty acids [PATH:ko01040]	acyl-CoA thioesterase II [EC:3.1.2.-]	K10805	0	0	0	0	1

Level 1	Level 2	Level 3	Function	KEGG ID	Sequence Reads				
					Day17	Day24	Day31	Day38	Bulk Soil
Metabolism	Lipid Metabolism	01040 Biosynthesis of unsaturated fatty acids [PATH:ko01040]	acyl-CoA thioesterase YciA [EC:3.1.2.-]	K10806	0	1	0	0	1
Metabolism	Metabolism of Cofactors and Vitamins	00130 Ubiquinone and other terpenoid-quinone biosynthesis [PATH:ko00130]	1,4-dihydroxy-2-naphthoate octaprenyltransferase	K02548	1	2	1	2	0
Metabolism	Metabolism of Cofactors and Vitamins	00130 Ubiquinone and other terpenoid-quinone biosynthesis [PATH:ko00130]	1,4-dihydroxy-6-naphthoate synthase [EC:1.14.-.-]	K11785	0	0	1	0	0
Metabolism	Metabolism of Cofactors and Vitamins	00130 Ubiquinone and other terpenoid-quinone biosynthesis [PATH:ko00130]	2-octaprenyl-6-methoxyphenol hydroxylase [EC:1.14.13.-]	K03185	0	0	0	2	0
Metabolism	Metabolism of Cofactors and Vitamins	00130 Ubiquinone and other terpenoid-quinone biosynthesis [PATH:ko00130]	2-succinyl-5-enolpyruvyl-6-hydroxy-3-cyclohexene-1-carboxyla	K02551	1	0	0	1	0
Metabolism	Metabolism of Cofactors and Vitamins	00130 Ubiquinone and other terpenoid-quinone biosynthesis [PATH:ko00130]	3-demethylubiquinone-9 3-methyltransferase	K00568	0	0	2	0	0
Metabolism	Metabolism of Cofactors and Vitamins	00130 Ubiquinone and other terpenoid-quinone biosynthesis [PATH:ko00130]	3-octaprenyl-4-hydroxybenzoate carboxy-lyase UbiD	K03182	2	3	0	3	0
Metabolism	Metabolism of Cofactors and Vitamins	00130 Ubiquinone and other terpenoid-quinone biosynthesis [PATH:ko00130]	4-hydroxybenzoate octaprenyltransferase [EC:2.5.1.-]	K03179	1	0	0	2	0

Level 1	Level 2	Level 3	Function	KEGG ID	Sequence Reads				
					Day17	Day24	Day31	Day38	Bulk Soil
Metabolism	Metabolism of Cofactors and Vitamins	00130 Ubiquinone and other terpenoid-quinone biosynthesis [PATH:ko00130]	4-hydroxyphenylpyruvate dioxygenase [EC:1.13.11.27]	K00457	2	2	0	0	0
Metabolism	Metabolism of Cofactors and Vitamins	00130 Ubiquinone and other terpenoid-quinone biosynthesis [PATH:ko00130]	naphthoate synthase [EC:4.1.3.36]	K01661	0	1	2	0	2
Metabolism	Metabolism of Cofactors and Vitamins	00130 Ubiquinone and other terpenoid-quinone biosynthesis [PATH:ko00130]	O-succinylbenzoate synthase [EC:4.2.1.113]	K02549	0	0	0	0	1
Metabolism	Metabolism of Cofactors and Vitamins	00130 Ubiquinone and other terpenoid-quinone biosynthesis [PATH:ko00130]	O-succinylbenzoic acid--CoA ligase [EC:6.2.1.26]	K01911	0	0	1	0	0
Metabolism	Metabolism of Cofactors and Vitamins	00130 Ubiquinone and other terpenoid-quinone biosynthesis [PATH:ko00130]	ubiquinone biosynthesis monooxygenase Coq7 [EC:1.14.13.-]	K06134	0	0	0	0	1
Metabolism	Metabolism of Cofactors and Vitamins	00130 Ubiquinone and other terpenoid-quinone biosynthesis [PATH:ko00130]	ubiquinone biosynthesis protein	K03688	0	0	1	0	3
Metabolism	Metabolism of Cofactors and Vitamins	00130 Ubiquinone and other terpenoid-quinone biosynthesis [PATH:ko00130]	ubiquinone/menaquinone biosynthesis methyltransferase	K03183	1	0	0	2	0
Metabolism	Metabolism of Cofactors and Vitamins	00670 One carbon pool by folate [PATH:ko00670]	5-methyltetrahydrofolate--homocysteine methyltransferase	K00548	6	13	0	0	0
Metabolism	Metabolism of Cofactors and Vitamins	00670 One carbon pool by folate [PATH:ko00670]	aminomethyltransferase [EC:2.1.2.10]	K00605	0	0	0	3	0

Level 1	Level 2	Level 3	Function	KEGG ID	Sequence Reads				
					Day17	Day24	Day31	Day38	Bulk Soil
Metabolism	Metabolism of Cofactors and Vitamins	00670 One carbon pool by folate [PATH:ko00670]	formate--tetrahydrofolate ligase [EC:6.3.4.3]	K01938	0	0	5	0	0
Metabolism	Metabolism of Cofactors and Vitamins	00670 One carbon pool by folate [PATH:ko00670]	formyltetrahydrofolate deformylase [EC:3.5.1.10]	K01433	0	0	2	1	0
Metabolism	Metabolism of Cofactors and Vitamins	00670 One carbon pool by folate [PATH:ko00670]	glycine hydroxymethyltransferase [EC:2.1.2.1]	K00600	5	3	0	0	7
Metabolism	Metabolism of Cofactors and Vitamins	00670 One carbon pool by folate [PATH:ko00670]	methylenetetrahydrofolate reductase (NADPH) [EC:1.5.1.20]	K00297	0	1	0	0	0
Metabolism	Metabolism of Cofactors and Vitamins	00670 One carbon pool by folate [PATH:ko00670]	phosphoribosylaminoimidazolecarboxamide formyltransferase /	K00602	0	0	0	5	4
Metabolism	Metabolism of Cofactors and Vitamins	00670 One carbon pool by folate [PATH:ko00670]	phosphoribosylglycinamide formyltransferase 2 [EC:2.1.2.2]	K08289	4	0	0	0	0
Metabolism	Metabolism of Cofactors and Vitamins	00670 One carbon pool by folate [PATH:ko00670]	thymidylate synthase [EC:2.1.1.45]	K00560	0	0	0	0	2
Metabolism	Metabolism of Cofactors and Vitamins	00730 Thiamine metabolism [PATH:ko00730]	cysteine desulfurase / selenocysteine lyase [EC:2.8.1.7	K11717	0	0	0	6	0
Metabolism	Metabolism of Cofactors and Vitamins	00730 Thiamine metabolism [PATH:ko00730]	cysteine desulfurase [EC:2.8.1.7]	K04487	0	3	0	0	0
Metabolism	Metabolism of Cofactors and Vitamins	00730 Thiamine metabolism [PATH:ko00730]	hydroxymethylpyrimidine/phosphomethylpyrimidine kinase	K00941	1	0	0	2	0
Metabolism	Metabolism of Cofactors and Vitamins	00730 Thiamine metabolism [PATH:ko00730]	nucleoside-triphosphatase [EC:3.6.1.15]	K01516	0	0	2	0	5
Metabolism	Metabolism of Cofactors and Vitamins	00730 Thiamine metabolism [PATH:ko00730]	thiamine biosynthesis ThiG	K03149	0	3	3	0	4
Metabolism	Metabolism of Cofactors and Vitamins	00730 Thiamine metabolism [PATH:ko00730]	thiamine pyrophosphokinase [EC:2.7.6.2]	K00949	0	0	1	0	0

Level 1	Level 2	Level 3	Function	KEGG ID	Sequence Reads				
					Day17	Day24	Day31	Day38	Bulk Soil
Metabolism	Metabolism of Cofactors and Vitamins	00730 Thiamine metabolism [PATH:ko00730]	thiamine-monophosphate kinase [EC:2.7.4.16]	K00946	0	0	3	0	0
Metabolism	Metabolism of Cofactors and Vitamins	00740 Riboflavin metabolism [PATH:ko00740]	3,4-dihydroxy 2-butanone 4-phosphate synthase [EC:4.1.99.12]	K02858	0	0	0	7	0
Metabolism	Metabolism of Cofactors and Vitamins	00740 Riboflavin metabolism [PATH:ko00740]	diaminohydroxyphosphoribosylaminopyrimidine deaminase /	K11752	1	3	0	0	0
Metabolism	Metabolism of Cofactors and Vitamins	00740 Riboflavin metabolism [PATH:ko00740]	riboflavin synthase beta chain [EC:2.5.1.-]	K00794	1	0	0	0	1
Metabolism	Metabolism of Cofactors and Vitamins	00750 Vitamin B6 metabolism [PATH:ko00750]	4-hydroxythreonine-4-phosphate dehydrogenase [EC:1.1.1.262]	K00097	2	0	0	0	0
Metabolism	Metabolism of Cofactors and Vitamins	00750 Vitamin B6 metabolism [PATH:ko00750]	phosphoserine aminotransferase [EC:2.6.1.52]	K00831	5	3	0	4	0
Metabolism	Metabolism of Cofactors and Vitamins	00750 Vitamin B6 metabolism [PATH:ko00750]	pyridoxine 4-dehydrogenase [EC:1.1.1.65]	K05275	1	0	0	0	0
Metabolism	Metabolism of Cofactors and Vitamins	00750 Vitamin B6 metabolism [PATH:ko00750]	pyridoxine biosynthesis protein [EC:4.-.-.-]	K06215	1	4	0	1	1
Metabolism	Metabolism of Cofactors and Vitamins	00750 Vitamin B6 metabolism [PATH:ko00750]	pyridoxine kinase [EC:2.7.1.35]	K00868	0	1	0	0	1
Metabolism	Metabolism of Cofactors and Vitamins	00750 Vitamin B6 metabolism [PATH:ko00750]	threonine synthase [EC:4.2.3.1]	K01733	2	8	3	0	5
Metabolism	Metabolism of Cofactors and Vitamins	00760 Nicotinate and nicotinamide metabolism [PATH:ko00760]	5'-nucleotidase [EC:3.1.3.5]	K01081	0	0	1	4	0
Metabolism	Metabolism of Cofactors and Vitamins	00760 Nicotinate and nicotinamide metabolism [PATH:ko00760]	5'-nucleotidase [EC:3.1.3.5]	K03787	0	3	0	0	0

Level 1	Level 2	Level 3	Function	KEGG ID	Sequence Reads				
					Day17	Day24	Day31	Day38	Bulk Soil
Metabolism	Metabolism of Cofactors and Vitamins	00760 Nicotinate and nicotinamide metabolism [PATH:ko00760]	5'-nucleotidase [EC:3.1.3.5]	K08722	1	0	0	0	0
Metabolism	Metabolism of Cofactors and Vitamins	00760 Nicotinate and nicotinamide metabolism [PATH:ko00760]	maleate isomerase [EC:5.2.1.1]	K01799	0	1	0	0	0
Metabolism	Metabolism of Cofactors and Vitamins	00760 Nicotinate and nicotinamide metabolism [PATH:ko00760]	NAD(P) transhydrogenase [EC:1.6.1.1]	K00322	0	2	1	0	2
Metabolism	Metabolism of Cofactors and Vitamins	00760 Nicotinate and nicotinamide metabolism [PATH:ko00760]	NAD(P) transhydrogenase subunit alpha [EC:1.6.1.2]	K00324	0	3	4	0	8
Metabolism	Metabolism of Cofactors and Vitamins	00760 Nicotinate and nicotinamide metabolism [PATH:ko00760]	NAD(P) transhydrogenase subunit beta [EC:1.6.1.2]	K00325	7	2	3	3	2
Metabolism	Metabolism of Cofactors and Vitamins	00760 Nicotinate and nicotinamide metabolism [PATH:ko00760]	NAD+ kinase [EC:2.7.1.23]	K00858	0	3	0	1	0
Metabolism	Metabolism of Cofactors and Vitamins	00760 Nicotinate and nicotinamide metabolism [PATH:ko00760]	NAD+ synthase (glutamine-hydrolysing) [EC:6.3.5.1]	K01950	0	0	1	5	3
Metabolism	Metabolism of Cofactors and Vitamins	00760 Nicotinate and nicotinamide metabolism [PATH:ko00760]	NAD+ synthase [EC:6.3.1.5]	K01916	0	2	0	1	0
Metabolism	Metabolism of Cofactors and Vitamins	00760 Nicotinate and nicotinamide metabolism [PATH:ko00760]	nicotinate phosphoribosyltransferase [EC:2.4.2.11]	K00763	3	2	0	4	0
Metabolism	Metabolism of Cofactors and Vitamins	00760 Nicotinate and nicotinamide metabolism [PATH:ko00760]	nicotinate-nucleotide pyrophosphorylase (carboxylating)	K00767	0	3	0	0	0

Level 1	Level 2	Level 3	Function	KEGG ID	Sequence Reads				
					Day17	Day24	Day31	Day38	Bulk Soil
Metabolism	Metabolism of Cofactors and Vitamins	00760 Nicotinate and nicotinamide metabolism [PATH:ko00760]	purine-nucleoside phosphorylase [EC:2.4.2.1]	K03784	1	0	0	0	0
Metabolism	Metabolism of Cofactors and Vitamins	00760 Nicotinate and nicotinamide metabolism [PATH:ko00760]	quinolinate synthase [EC:2.5.1.72]	K03517	2	6	2	0	0
Metabolism	Metabolism of Cofactors and Vitamins	00770 Pantothenate and CoA biosynthesis [PATH:ko00770]	3-methyl-2-oxobutanoate hydroxymethyltransferase	K00606	2	0	2	0	0
Metabolism	Metabolism of Cofactors and Vitamins	00770 Pantothenate and CoA biosynthesis [PATH:ko00770]	acetolactate synthase I/III small subunit [EC:2.2.1.6]	K01653	0	0	0	1	0
Metabolism	Metabolism of Cofactors and Vitamins	00770 Pantothenate and CoA biosynthesis [PATH:ko00770]	aspartate 1-decarboxylase [EC:4.1.1.11]	K01579	1	0	0	1	0
Metabolism	Metabolism of Cofactors and Vitamins	00770 Pantothenate and CoA biosynthesis [PATH:ko00770]	branched-chain amino acid aminotransferase [EC:2.6.1.42]	K00826	0	5	0	0	0
Metabolism	Metabolism of Cofactors and Vitamins	00770 Pantothenate and CoA biosynthesis [PATH:ko00770]	dihydroxy-acid dehydratase [EC:4.2.1.9]	K01687	3	0	5	0	7
Metabolism	Metabolism of Cofactors and Vitamins	00770 Pantothenate and CoA biosynthesis [PATH:ko00770]	holo-[acyl-carrier protein] synthase [EC:2.7.8.7]	K00997	0	0	2	1	0
Metabolism	Metabolism of Cofactors and Vitamins	00770 Pantothenate and CoA biosynthesis [PATH:ko00770]	ketol-acid reductoisomerase [EC:1.1.1.86]	K00053	3	0	1	2	0
Metabolism	Metabolism of Cofactors and Vitamins	00770 Pantothenate and CoA biosynthesis [PATH:ko00770]	pantetheine-phosphate adenylyltransferase [EC:2.7.7.3]	K00954	1	0	1	2	0

Level 1	Level 2	Level 3	Function	KEGG ID	Sequence Reads				
					Day17	Day24	Day31	Day38	Bulk Soil
Metabolism	Metabolism of Cofactors and Vitamins	00770 Pantothenate and CoA biosynthesis [PATH:ko00770]	pantoate--beta-alanine ligase [EC:6.3.2.1]	K01918	1	0	0	0	0
Metabolism	Metabolism of Cofactors and Vitamins	00770 Pantothenate and CoA biosynthesis [PATH:ko00770]	phosphopantothenoylcysteine decarboxylase /	K13038	4	0	0	3	1
Metabolism	Metabolism of Cofactors and Vitamins	00770 Pantothenate and CoA biosynthesis [PATH:ko00770]	type I pantothenate kinase [EC:2.7.1.33]	K00867	0	0	2	0	0
Metabolism	Metabolism of Cofactors and Vitamins	00770 Pantothenate and CoA biosynthesis [PATH:ko00770]	type III pantothenate kinase [EC:2.7.1.33]	K03525	2	0	0	0	1
Metabolism	Metabolism of Cofactors and Vitamins	00780 Biotin metabolism [PATH:ko00780]	[EC:3.4.-.-]	K01423	0	0	0	0	3
Metabolism	Metabolism of Cofactors and Vitamins	00780 Biotin metabolism [PATH:ko00780]	8-amino-7-oxononanoate synthase [EC:2.3.1.47]	K00652	2	0	1	0	0
Metabolism	Metabolism of Cofactors and Vitamins	00780 Biotin metabolism [PATH:ko00780]	biotin synthetase [EC:2.8.1.6]	K01012	0	1	0	2	0
Metabolism	Metabolism of Cofactors and Vitamins	00780 Biotin metabolism [PATH:ko00780]	dethiobiotin synthetase [EC:6.3.3.3]	K01935	0	0	1	0	1
Metabolism	Metabolism of Cofactors and Vitamins	00785 Lipoic acid metabolism [PATH:ko00785]	lipoyl(octanoyl) transferase [EC:2.3.1.181]	K03801	0	0	0	0	1
Metabolism	Metabolism of Cofactors and Vitamins	00790 Folate biosynthesis [PATH:ko00790]	dihydrofolate reductase [EC:1.5.1.3]	K00287	0	0	1	0	0
Metabolism	Metabolism of Cofactors and Vitamins	00790 Folate biosynthesis [PATH:ko00790]	dihydrofolate synthase / folylpolyglutamate synthase	K11754	2	0	0	2	3
Metabolism	Metabolism of Cofactors and Vitamins	00790 Folate biosynthesis [PATH:ko00790]	dihydroneopterin aldolase [EC:4.1.2.25]	K01633	1	0	1	0	1

Level 1	Level 2	Level 3	Function	KEGG ID	Sequence Reads				
					Day17	Day24	Day31	Day38	Bulk Soil
Metabolism	Metabolism of Cofactors and Vitamins	00790 Folate biosynthesis [PATH:ko00790]	dihydropteroate synthase [EC:2.5.1.15]	K00796	0	2	0	0	2
Metabolism	Metabolism of Cofactors and Vitamins	00790 Folate biosynthesis [PATH:ko00790]	para-aminobenzoate synthetase / 4-amino-4-deoxychorismate	K03342	0	0	0	1	0
Metabolism	Metabolism of Cofactors and Vitamins	00790 Folate biosynthesis [PATH:ko00790]	para-aminobenzoate synthetase component I [EC:2.6.1.85]	K01665	0	2	0	0	0
Metabolism	Metabolism of Cofactors and Vitamins	00860 Porphyrin and chlorophyll metabolism [PATH:ko00860]	adenosylcobinamide-phosphate synthase CobD [EC:6.3.1.10]	K02227	0	1	0	0	0
Metabolism	Metabolism of Cofactors and Vitamins	00860 Porphyrin and chlorophyll metabolism [PATH:ko00860]	adenosylcobyric acid synthase [EC:6.3.5.10]	K02232	0	1	0	0	3
Metabolism	Metabolism of Cofactors and Vitamins	00860 Porphyrin and chlorophyll metabolism [PATH:ko00860]	cob(I)alamin adenosyltransferase [EC:2.5.1.17]	K00798	0	4	0	3	4
Metabolism	Metabolism of Cofactors and Vitamins	00860 Porphyrin and chlorophyll metabolism [PATH:ko00860]	cobalamin biosynthesis protein CbiG / precorrin-3B	K13541	0	0	0	2	0
Metabolism	Metabolism of Cofactors and Vitamins	00860 Porphyrin and chlorophyll metabolism [PATH:ko00860]	cobaltochelatase CobN [EC:6.6.1.2]	K02230	0	3	0	0	2
Metabolism	Metabolism of Cofactors and Vitamins	00860 Porphyrin and chlorophyll metabolism [PATH:ko00860]	cobaltochelatase CobS [EC:6.6.1.2]	K09882	0	2	0	0	1
Metabolism	Metabolism of Cofactors and Vitamins	00860 Porphyrin and chlorophyll metabolism [PATH:ko00860]	cobaltochelatase CobT [EC:6.6.1.2]	K09883	0	0	0	3	0
Metabolism	Metabolism of Cofactors and Vitamins	00860 Porphyrin and chlorophyll metabolism [PATH:ko00860]	coproporphyrinogen III oxidase [EC:1.3.3.3]	K00228	0	0	2	0	0

Level 1	Level 2	Level 3	Function	KEGG ID	Sequence Reads				
					Day17	Day24	Day31	Day38	Bulk Soil
Metabolism	Metabolism of Cofactors and Vitamins	00860 Porphyrin and chlorophyll metabolism [PATH:ko00860]	ferrochelatase [EC:4.99.1.1]	K01772	2	0	0	3	0
Metabolism	Metabolism of Cofactors and Vitamins	00860 Porphyrin and chlorophyll metabolism [PATH:ko00860]	FMN reductase [EC:1.5.1.29]	K00299	0	0	0	1	0
Metabolism	Metabolism of Cofactors and Vitamins	00860 Porphyrin and chlorophyll metabolism [PATH:ko00860]	glutamate-1-semialdehyde 2,1-aminomutase [EC:5.4.3.8]	K01845	0	0	0	3	10
Metabolism	Metabolism of Cofactors and Vitamins	00860 Porphyrin and chlorophyll metabolism [PATH:ko00860]	glutamyl-tRNA reductase [EC:1.2.1.70]	K02492	2	0	0	0	0
Metabolism	Metabolism of Cofactors and Vitamins	00860 Porphyrin and chlorophyll metabolism [PATH:ko00860]	heme oxygenase [EC:1.14.99.3]	K00510	0	0	1	0	0
Metabolism	Metabolism of Cofactors and Vitamins	00860 Porphyrin and chlorophyll metabolism [PATH:ko00860]	hydroxymethylbilane synthase [EC:2.5.1.61]	K01749	0	0	0	3	0
Metabolism	Metabolism of Cofactors and Vitamins	00860 Porphyrin and chlorophyll metabolism [PATH:ko00860]	light-independent protochlorophyllide reductase subunit L	K04037	1	0	1	0	0
Metabolism	Metabolism of Cofactors and Vitamins	00860 Porphyrin and chlorophyll metabolism [PATH:ko00860]	light-independent protochlorophyllide reductase subunit N	K04038	0	0	1	1	0
Metabolism	Metabolism of Cofactors and Vitamins	00860 Porphyrin and chlorophyll metabolism [PATH:ko00860]	magnesium chelatase subunit D [EC:6.6.1.1]	K03404	2	0	0	2	0
Metabolism	Metabolism of Cofactors and Vitamins	00860 Porphyrin and chlorophyll metabolism [PATH:ko00860]	magnesium chelatase subunit H [EC:6.6.1.1]	K03403	0	0	1	1	0

Level 1	Level 2	Level 3	Function	KEGG ID	Sequence Reads				
					Day17	Day24	Day31	Day38	Bulk Soil
Metabolism	Metabolism of Cofactors and Vitamins	00860 Porphyrin and chlorophyll metabolism [PATH:ko00860]	magnesium chelatase subunit I [EC:6.6.1.1]	K03405	5	5	0	3	5
Metabolism	Metabolism of Cofactors and Vitamins	00860 Porphyrin and chlorophyll metabolism [PATH:ko00860]	magnesium-protoporphyrin IX monomethyl ester (oxidative)	K04035	3	0	0	2	0
Metabolism	Metabolism of Cofactors and Vitamins	00860 Porphyrin and chlorophyll metabolism [PATH:ko00860]	nicotinate-nucleotide--dimethylbenzimidazole	K00768	1	1	1	0	0
Metabolism	Metabolism of Cofactors and Vitamins	00860 Porphyrin and chlorophyll metabolism [PATH:ko00860]	oxygen-independent coproporphyrinogen III oxidase	K02495	0	1	0	0	4
Metabolism	Metabolism of Cofactors and Vitamins	00860 Porphyrin and chlorophyll metabolism [PATH:ko00860]	porphobilinogen synthase [EC:4.2.1.24]	K01698	1	0	0	0	0
Metabolism	Metabolism of Cofactors and Vitamins	00860 Porphyrin and chlorophyll metabolism [PATH:ko00860]	precorrin-2/cobalt-factor-2 C20-methyltransferase	K03394	0	0	1	0	0
Metabolism	Metabolism of Cofactors and Vitamins	00860 Porphyrin and chlorophyll metabolism [PATH:ko00860]	precorrin-4 C11-methyltransferase [EC:2.1.1.133]	K05936	0	0	0	3	1
Metabolism	Metabolism of Cofactors and Vitamins	00860 Porphyrin and chlorophyll metabolism [PATH:ko00860]	precorrin-6Y C5,15-methyltransferase / precorrin-8W	K00595	0	0	1	0	0
Metabolism	Metabolism of Cofactors and Vitamins	00860 Porphyrin and chlorophyll metabolism [PATH:ko00860]	precorrin-8X methylmutase [EC:5.4.1.2]	K06042	0	0	0	1	0
Metabolism	Metabolism of Cofactors and Vitamins	00860 Porphyrin and chlorophyll metabolism [PATH:ko00860]	protoheme IX farnesyltransferase [EC:2.5.1.-]	K02301	0	0	0	2	2

Level 1	Level 2	Level 3	Function	KEGG ID	Sequence Reads				
					Day17	Day24	Day31	Day38	Bulk Soil
Metabolism	Metabolism of Cofactors and Vitamins	00860 Porphyrin and chlorophyll metabolism [PATH:ko00860]	protoporphyrinogen oxidase [EC:1.3.3.4]	K00231	0	2	0	0	0
Metabolism	Metabolism of Cofactors and Vitamins	00860 Porphyrin and chlorophyll metabolism [PATH:ko00860]	uroporphyrin-III C-methyltransferase / precorrin-2	K02302	0	2	0	0	3
Metabolism	Metabolism of Cofactors and Vitamins	00860 Porphyrin and chlorophyll metabolism [PATH:ko00860]	uroporphyrinogen decarboxylase [EC:4.1.1.37]	K01599	0	1	2	4	2
Metabolism	Metabolism of Other Amino Acids	00410 beta-Alanine metabolism [PATH:ko00410]	4-aminobutyrate aminotransferase /	K07250	2	0	3	0	0
Metabolism	Metabolism of Other Amino Acids	00410 beta-Alanine metabolism [PATH:ko00410]	beta-alanine--pyruvate transaminase [EC:2.6.1.18]	K00822	1	0	0	0	1
Metabolism	Metabolism of Other Amino Acids	00430 Taurine and hypotaurine metabolism [PATH:ko00430]	alanine dehydrogenase [EC:1.4.1.1]	K00259	5	1	3	2	0
Metabolism	Metabolism of Other Amino Acids	00430 Taurine and hypotaurine metabolism [PATH:ko00430]	phosphate acetyltransferase [EC:2.3.1.8]	K00625	0	1	0	0	0
Metabolism	Metabolism of Other Amino Acids	00430 Taurine and hypotaurine metabolism [PATH:ko00430]	phosphate acetyltransferase [EC:2.3.1.8]	K13788	0	0	0	0	3
Metabolism	Metabolism of Other Amino Acids	00450 Selenoamino acid metabolism [PATH:ko00450]	adenosylhomocysteinase [EC:3.3.1.1]	K01251	15	8	13	6	3
Metabolism	Metabolism of Other Amino Acids	00450 Selenoamino acid metabolism [PATH:ko00450]	bifunctional enzyme CysN/CysC [EC:2.7.7.4 2.7.1.25]	K00955	1	1	2	0	0
Metabolism	Metabolism of Other Amino Acids	00450 Selenoamino acid metabolism [PATH:ko00450]	cystathionine beta-synthase [EC:4.2.1.22]	K01697	0	5	5	0	3

Level 1	Level 2	Level 3	Function	KEGG ID	Sequence Reads				
					Day17	Day24	Day31	Day38	Bulk Soil
Metabolism	Metabolism of Other Amino Acids	00450 Selenoamino acid metabolism [PATH:ko00450]	cystathionine gamma-lyase [EC:4.4.1.1]	K01758	0	0	0	1	0
Metabolism	Metabolism of Other Amino Acids	00450 Selenoamino acid metabolism [PATH:ko00450]	cystathionine gamma-synthase [EC:2.5.1.48]	K01739	0	4	0	0	0
Metabolism	Metabolism of Other Amino Acids	00450 Selenoamino acid metabolism [PATH:ko00450]	cysteine synthase A [EC:2.5.1.47]	K01738	6	2	6	6	0
Metabolism	Metabolism of Other Amino Acids	00450 Selenoamino acid metabolism [PATH:ko00450]	S-adenosylmethionine synthetase [EC:2.5.1.6]	K00789	9	4	2	7	2
Metabolism	Metabolism of Other Amino Acids	00450 Selenoamino acid metabolism [PATH:ko00450]	selenide, water dikinase [EC:2.7.9.3]	K01008	2	0	0	0	1
Metabolism	Metabolism of Other Amino Acids	00450 Selenoamino acid metabolism [PATH:ko00450]	sulfate adenylyltransferase [EC:2.7.7.4]	K00958	0	0	0	0	1
Metabolism	Metabolism of Other Amino Acids	00450 Selenoamino acid metabolism [PATH:ko00450]	sulfate adenylyltransferase subunit 1 [EC:2.7.7.4]	K00956	0	0	0	3	2
Metabolism	Metabolism of Other Amino Acids	00450 Selenoamino acid metabolism [PATH:ko00450]	sulfite reductase (ferredoxin) [EC:1.8.7.1]	K00392	0	0	0	1	0
Metabolism	Metabolism of Other Amino Acids	00460 Cyanoamino acid metabolism [PATH:ko00460]	aldoxime dehydratase [EC:4.99.1.5]	K13028	0	0	1	0	0
Metabolism	Metabolism of Other Amino Acids	00460 Cyanoamino acid metabolism [PATH:ko00460]	gamma-glutamyltranspeptidase [EC:2.3.2.2]	K00681	0	0	0	5	0
Metabolism	Metabolism of Other Amino Acids	00460 Cyanoamino acid metabolism [PATH:ko00460]	L-asparaginase [EC:3.5.1.1]	K01424	76	2	53	1	0
Metabolism	Metabolism of Other Amino Acids	00480 Glutathione metabolism [PATH:ko00480]	5-oxoprolinase (ATP-hydrolysing) [EC:3.5.2.9]	K01469	0	2	4	0	2
Metabolism	Metabolism of Other Amino Acids	00480 Glutathione metabolism [PATH:ko00480]	6-phosphogluconate dehydrogenase [EC:1.1.1.44]	K00033	3	5	4	2	0

Level 1	Level 2	Level 3	Function	KEGG ID	Sequence Reads				
					Day17	Day24	Day31	Day38	Bulk Soil
Metabolism	Metabolism of Other Amino Acids	00480 Glutathione metabolism [PATH:ko00480]	aminopeptidase N [EC:3.4.11.2]	K01256	0	0	2	5	0
Metabolism	Metabolism of Other Amino Acids	00480 Glutathione metabolism [PATH:ko00480]	glucose-6-phosphate 1-dehydrogenase [EC:1.1.1.49]	K00036	4	7	0	10	0
Metabolism	Metabolism of Other Amino Acids	00480 Glutathione metabolism [PATH:ko00480]	glutamate--cysteine ligase [EC:6.3.2.2]	K01919	0	2	4	1	3
Metabolism	Metabolism of Other Amino Acids	00480 Glutathione metabolism [PATH:ko00480]	glutathione peroxidase [EC:1.11.1.9]	K00432	0	0	0	0	1
Metabolism	Metabolism of Other Amino Acids	00480 Glutathione metabolism [PATH:ko00480]	glutathione reductase (NADPH) [EC:1.8.1.7]	K00383	0	3	0	0	0
Metabolism	Metabolism of Other Amino Acids	00480 Glutathione metabolism [PATH:ko00480]	L-ascorbate peroxidase [EC:1.11.1.11]	K00434	0	1	0	0	0
Metabolism	Metabolism of Other Amino Acids	00480 Glutathione metabolism [PATH:ko00480]	leucyl aminopeptidase [EC:3.4.11.1]	K01255	6	5	3	0	0
Metabolism	Metabolism of Other Amino Acids	00480 Glutathione metabolism [PATH:ko00480]	ornithine decarboxylase [EC:4.1.1.17]	K01581	0	0	2	0	1
Metabolism	Metabolism of Other Amino Acids	00480 Glutathione metabolism [PATH:ko00480]	ribonucleoside-diphosphate reductase subunit M1	K10807	1	0	0	0	0
Metabolism	Metabolism of Other Amino Acids	00480 Glutathione metabolism [PATH:ko00480]	spermidine synthase [EC:2.5.1.16]	K00797	2	0	0	0	0
Metabolism	Metabolism of Terpenoids and Polyketides	00253 Tetracycline biosynthesis [PATH:ko00253]	acetyl-CoA carboxylase biotin carboxyl carrier protein	K02160	0	0	2	0	0
Metabolism	Metabolism of Terpenoids and Polyketides	00253 Tetracycline biosynthesis [PATH:ko00253]	acetyl-CoA carboxylase carboxyl transferase subunit beta	K01963	1	3	1	3	0
Metabolism	Metabolism of Terpenoids and Polyketides	00253 Tetracycline biosynthesis [PATH:ko00253]	acetyl-CoA carboxylase, biotin carboxylase subunit	K01961	0	0	4	0	0

Level 1	Level 2	Level 3	Function	KEGG ID	Sequence Reads				
					Day17	Day24	Day31	Day38	Bulk Soil
Metabolism	Metabolism of Terpenoids and Polyketides	00281 Geraniol degradation [PATH:ko00281]	citronellol/citronellal dehydrogenase	K13775	0	2	1	0	0
Metabolism	Metabolism of Terpenoids and Polyketides	00281 Geraniol degradation [PATH:ko00281]	geranyl-CoA carboxylase alpha subunit [EC:6.4.1.5]	K13777	0	1	0	0	0
Metabolism	Metabolism of Terpenoids and Polyketides	00281 Geraniol degradation [PATH:ko00281]	geranyl-CoA carboxylase beta subunit [EC:6.4.1.5]	K13778	0	2	0	1	0
Metabolism	Metabolism of Terpenoids and Polyketides	00900 Terpenoid backbone biosynthesis [PATH:ko00900]	(E)-4-hydroxy-3-methylbut-2-enyl-diphosphate synthase	K03526	2	2	3	4	6
Metabolism	Metabolism of Terpenoids and Polyketides	00900 Terpenoid backbone biosynthesis [PATH:ko00900]	2-C-methyl-D-erythritol 4-phosphate cytidylyltransferase /	K12506	0	0	2	1	0
Metabolism	Metabolism of Terpenoids and Polyketides	00900 Terpenoid backbone biosynthesis [PATH:ko00900]	4-diphosphocytidyl-2-C-methyl-D-erythritol kinase	K00919	0	1	0	0	3
Metabolism	Metabolism of Terpenoids and Polyketides	00900 Terpenoid backbone biosynthesis [PATH:ko00900]	4-hydroxy-3-methylbut-2-enyl diphosphate reductase	K03527	0	2	0	2	0
Metabolism	Metabolism of Terpenoids and Polyketides	00900 Terpenoid backbone biosynthesis [PATH:ko00900]	farnesyl diphosphate synthase [EC:2.5.1.1 2.5.1.10]	K00795	2	1	0	0	3
Metabolism	Metabolism of Terpenoids and Polyketides	00900 Terpenoid backbone biosynthesis [PATH:ko00900]	geranylgeranyl diphosphate synthase, type II [EC:2.5.1.1	K13789	2	0	0	0	0
Metabolism	Metabolism of Terpenoids and Polyketides	00900 Terpenoid backbone biosynthesis [PATH:ko00900]	hydroxymethylglutaryl-CoA reductase [EC:1.1.1.88]	K00054	1	0	0	2	0
Metabolism	Metabolism of Terpenoids and Polyketides	00900 Terpenoid backbone biosynthesis [PATH:ko00900]	isopentenyl-diphosphate delta-isomerase [EC:5.3.3.2]	K01823	2	0	0	0	0

Level 1	Level 2	Level 3	Function	KEGG ID	Sequence Reads				
					Day17	Day24	Day31	Day38	Bulk Soil
Metabolism	Metabolism of Terpenoids and Polyketides	00900 Terpenoid backbone biosynthesis [PATH:ko00900]	undecaprenyl diphosphate synthase [EC:2.5.1.31]	K00806	1	0	0	1	0
Metabolism	Metabolism of Terpenoids and Polyketides	00903 Limonene and pinene degradation [PATH:ko00903]	(+)-trans-carveol dehydrogenase [EC:1.1.1.275]	K12466	0	0	1	0	0
Metabolism	Metabolism of Terpenoids and Polyketides	00903 Limonene and pinene degradation [PATH:ko00903]	[EC:1.1.99.-]	K00119	0	0	2	0	0
Metabolism	Metabolism of Terpenoids and Polyketides	00903 Limonene and pinene degradation [PATH:ko00903]	[EC:3.1.2.-]	K01076	0	0	0	0	1
Metabolism	Metabolism of Terpenoids and Polyketides	00906 Carotenoid biosynthesis [PATH:ko00906]	beta-carotene ketolase (CrtO type)	K02292	0	0	0	1	0
Metabolism	Metabolism of Terpenoids and Polyketides	00906 Carotenoid biosynthesis [PATH:ko00906]	zeta-carotene desaturase [EC:1.14.99.30]	K00514	0	0	0	1	0
Metabolism	Metabolism of Terpenoids and Polyketides	00908 Zeatin biosynthesis [PATH:ko00908]	tRNA dimethylallyltransferase [EC:2.5.1.75]	K00791	0	0	0	0	2
Metabolism	Metabolism of Terpenoids and Polyketides	01051 Biosynthesis of ansamycins [PATH:ko01051]	transketolase [EC:2.2.1.1]	K00615	0	0	7	0	7
Metabolism	Metabolism of Terpenoids and Polyketides	01053 Biosynthesis of siderophore group nonribosomal peptides [PATH:ko01053]	isochorismatase [EC:3.3.2.1]	K05993	1	0	2	0	1
Metabolism	Metabolism of Terpenoids and Polyketides	01053 Biosynthesis of siderophore group nonribosomal peptides [PATH:ko01053]	mycobactin peptide synthetase MbtF	K04792	0	1	0	0	0
Metabolism	Nucleotide Metabolism	00230 Purine metabolism [PATH:ko00230]	3',5'-cyclic-nucleotide phosphodiesterase [EC:3.1.4.17]	K01120	0	0	0	1	0
Metabolism	Nucleotide Metabolism	00230 Purine metabolism	5-(carboxyamino)imidazole ribonucleotide mutase	K01588	1	0	0	0	2

Level 1	Level 2	Level 3	Function	KEGG ID	Sequence Reads				
					Day17	Day24	Day31	Day38	Bulk Soil
		[PATH:ko00230]							
Metabolism	Nucleotide Metabolism	00230 Purine metabolism [PATH:ko00230]	5-hydroxyisourate hydrolase [EC:3.5.2.17]	K07127	0	0	0	0	2
Metabolism	Nucleotide Metabolism	00230 Purine metabolism [PATH:ko00230]	adenosine deaminase [EC:3.5.4.4]	K01488	0	3	1	0	0
Metabolism	Nucleotide Metabolism	00230 Purine metabolism [PATH:ko00230]	adenosine kinase [EC:2.7.1.20]	K00856	0	0	2	0	0
Metabolism	Nucleotide Metabolism	00230 Purine metabolism [PATH:ko00230]	allantoinase [EC:3.5.2.5]	K01466	1	0	0	0	0
Metabolism	Nucleotide Metabolism	00230 Purine metabolism [PATH:ko00230]	AMP deaminase [EC:3.5.4.6]	K01490	0	0	1	0	0
Metabolism	Nucleotide Metabolism	00230 Purine metabolism [PATH:ko00230]	bis(5'-nucleosyl)-tetraphosphatase (symmetrical)	K01525	0	0	2	0	0
Metabolism	Nucleotide Metabolism	00230 Purine metabolism [PATH:ko00230]	dGTPase [EC:3.1.5.1]	K01129	0	3	1	1	1
Metabolism	Nucleotide Metabolism	00230 Purine metabolism [PATH:ko00230]	exopolyphosphatase [EC:3.6.1.11]	K01514	0	2	0	0	0
Metabolism	Nucleotide Metabolism	00230 Purine metabolism [PATH:ko00230]	GMP reductase [EC:1.7.1.7]	K00364	0	0	0	0	1
Metabolism	Nucleotide Metabolism	00230 Purine metabolism [PATH:ko00230]	GTP pyrophosphokinase [EC:2.7.6.5]	K00951	4	4	0	0	0
Metabolism	Nucleotide Metabolism	00230 Purine metabolism [PATH:ko00230]	guanine deaminase [EC:3.5.4.3]	K01487	1	0	1	0	0
Metabolism	Nucleotide Metabolism	00230 Purine metabolism [PATH:ko00230]	guanosine-3',5'-bis(diphosphate) 3'-pyrophosphohydrolase	K01139	0	1	0	0	0
Metabolism	Nucleotide Metabolism	00230 Purine metabolism [PATH:ko00230]	guanylate kinase [EC:2.7.4.8]	K00942	3	0	0	2	0

Level 1	Level 2	Level 3	Function	KEGG ID	Sequence Reads				
					Day17	Day24	Day31	Day38	Bulk Soil
Metabolism	Nucleotide Metabolism	00230 Purine metabolism [PATH:ko00230]	phosphopentomutase [EC:5.4.2.7]	K01839	0	0	0	1	1
Metabolism	Nucleotide Metabolism	00230 Purine metabolism [PATH:ko00230]	phosphoribosylamine--glycine ligase [EC:6.3.4.13]	K01945	0	1	4	2	1
Metabolism	Nucleotide Metabolism	00230 Purine metabolism [PATH:ko00230]	phosphoribosylaminoimidazole-succinocarboxamide synthase	K01923	0	0	7	5	6
Metabolism	Nucleotide Metabolism	00230 Purine metabolism [PATH:ko00230]	phosphoribosylformylglycinamidine cyclo-ligase [EC:6.3.3.1]	K01933	0	1	0	0	1
Metabolism	Nucleotide Metabolism	00230 Purine metabolism [PATH:ko00230]	phosphoribosylformylglycinamidine synthase [EC:6.3.5.3]	K01952	13	0	3	0	0
Metabolism	Nucleotide Metabolism	00230 Purine metabolism [PATH:ko00230]	ribose-phosphate pyrophosphokinase [EC:2.7.6.1]	K00948	4	2	2	0	0
Metabolism	Nucleotide Metabolism	00230 Purine metabolism [PATH:ko00230]	RNA-directed RNA polymerase [EC:2.7.7.48]	K00985	0	1	0	0	0
Metabolism	Nucleotide Metabolism	00230 Purine metabolism [PATH:ko00230]	xanthine dehydrogenase iron-sulfur-binding subunit	K13480	1	0	0	0	0
Metabolism	Nucleotide Metabolism	00230 Purine metabolism [PATH:ko00230]	xanthine dehydrogenase large subunit [EC:1.17.1.4]	K13482	0	1	0	1	2
Metabolism	Nucleotide Metabolism	00230 Purine metabolism [PATH:ko00230]	xanthine dehydrogenase molybdenum-binding subunit	K00087	1	0	0	0	0
Metabolism	Nucleotide Metabolism	00230 Purine metabolism [PATH:ko00230]	xanthine dehydrogenase small subunit [EC:1.17.1.4]	K13481	1	0	1	0	0
Metabolism	Nucleotide Metabolism	00230 Purine metabolism [PATH:ko00230]	xanthine dehydrogenase YagR molybdenum-binding subunit	K11177	0	4	0	0	5
Metabolism	Nucleotide Metabolism	00230 Purine metabolism [PATH:ko00230]	xanthine dehydrogenase YagS FAD-binding subunit	K11178	0	0	0	1	0
Metabolism	Nucleotide Metabolism	00230 Purine metabolism	xanthine dehydrogenase YagT iron-sulfur-binding subunit	K13483	0	0	1	0	0

Level 1	Level 2	Level 3	Function	KEGG ID	Sequence Reads				
					Day17	Day24	Day31	Day38	Bulk Soil
		[PATH:ko00230]							
Metabolism	Nucleotide Metabolism	00240 Pyrimidine metabolism [PATH:ko00240]	2',3'-cyclic-nucleotide 2'-phosphodiesterase [EC:3.1.4.16]	K01119	0	0	0	1	1
Metabolism	Nucleotide Metabolism	00240 Pyrimidine metabolism [PATH:ko00240]	3'-nucleotidase [EC:3.1.3.6]	K08693	0	0	0	0	1
Metabolism	Nucleotide Metabolism	00240 Pyrimidine metabolism [PATH:ko00240]	CTP synthase [EC:6.3.4.2]	K01937	8	4	5	0	0
Metabolism	Nucleotide Metabolism	00240 Pyrimidine metabolism [PATH:ko00240]	cytidylate kinase [EC:2.7.4.14]	K00945	0	2	1	0	0
Metabolism	Nucleotide Metabolism	00240 Pyrimidine metabolism [PATH:ko00240]	dCTP deaminase [EC:3.5.4.13]	K01494	6	3	1	0	0
Metabolism	Nucleotide Metabolism	00240 Pyrimidine metabolism [PATH:ko00240]	dihydroorotase [EC:3.5.2.3]	K01465	5	0	0	4	5
Metabolism	Nucleotide Metabolism	00240 Pyrimidine metabolism [PATH:ko00240]	dihydroorotate oxidase [EC:1.3.3.1]	K00226	0	0	1	0	0
Metabolism	Nucleotide Metabolism	00240 Pyrimidine metabolism [PATH:ko00240]	DNA-directed RNA polymerase subunit alpha [EC:2.7.7.6]	K03040	15	6	7	4	5
Metabolism	Nucleotide Metabolism	00240 Pyrimidine metabolism [PATH:ko00240]	dUTP pyrophosphatase [EC:3.6.1.23]	K01520	0	3	3	0	0
Metabolism	Nucleotide Metabolism	00240 Pyrimidine metabolism [PATH:ko00240]	nucleoside-diphosphate kinase [EC:2.7.4.6]	K00940	1	3	1	0	2
Metabolism	Nucleotide Metabolism	00240 Pyrimidine metabolism [PATH:ko00240]	nucleoside-triphosphate pyrophosphatase [EC:3.6.1.19]	K02428	0	0	2	2	0
Metabolism	Nucleotide Metabolism	00240 Pyrimidine metabolism [PATH:ko00240]	orotate phosphoribosyltransferase [EC:2.4.2.10]	K00762	0	1	3	0	1

Level 1	Level 2	Level 3	Function	KEGG ID	Sequence Reads				
					Day17	Day24	Day31	Day38	Bulk Soil
Metabolism	Nucleotide Metabolism	00240 Pyrimidine metabolism [PATH:ko00240]	orotidine-5'-phosphate decarboxylase [EC:4.1.1.23]	K01591	1	5	0	0	0
Metabolism	Nucleotide Metabolism	00240 Pyrimidine metabolism [PATH:ko00240]	pseudouridylate synthase [EC:4.2.1.70]	K01718	0	0	0	0	1
Metabolism	Nucleotide Metabolism	00240 Pyrimidine metabolism [PATH:ko00240]	pyrimidine operon attenuation protein / uracil	K02825	0	0	0	0	2
Metabolism	Nucleotide Metabolism	00240 Pyrimidine metabolism [PATH:ko00240]	pyrimidine-nucleoside phosphorylase [EC:2.4.2.2]	K00756	3	0	0	0	0
Metabolism	Nucleotide Metabolism	00240 Pyrimidine metabolism [PATH:ko00240]	ribonucleoside-diphosphate reductase alpha chain	K00525	0	10	4	8	10
Metabolism	Nucleotide Metabolism	00240 Pyrimidine metabolism [PATH:ko00240]	ribonucleoside-diphosphate reductase beta chain	K00526	2	0	0	0	0
Metabolism	Nucleotide Metabolism	00240 Pyrimidine metabolism [PATH:ko00240]	ribonucleoside-triphosphate reductase [EC:1.17.4.2]	K00527	1	0	0	0	0
Metabolism	Nucleotide Metabolism	00240 Pyrimidine metabolism [PATH:ko00240]	thioredoxin reductase (NADPH) [EC:1.8.1.9]	K00384	0	10	10	0	5
Metabolism	Nucleotide Metabolism	00240 Pyrimidine metabolism [PATH:ko00240]	uracil phosphoribosyltransferase [EC:2.4.2.9]	K00761	0	1	0	0	1
Metabolism	Nucleotide Metabolism	00240 Pyrimidine metabolism [PATH:ko00240]	uridylate kinase [EC:2.7.4.22]	K09903	2	0	0	0	0
Metabolism	Xenobiotics Biodegradation and Metabolism	00361 Chlorocyclohexane and chlorobenzene degradation [PATH:ko00361]	2,4-dichlorophenol 6-monooxygenase [EC:1.14.13.20]	K10676	0	0	1	0	0
Metabolism	Xenobiotics Biodegradation and Metabolism	00361 Chlorocyclohexane and chlorobenzene degradation [PATH:ko00361]	2-haloacid dehalogenase [EC:3.8.1.2]	K01560	1	0	0	0	0

Level 1	Level 2	Level 3	Function	KEGG ID	Sequence Reads				
					Day17	Day24	Day31	Day38	Bulk Soil
Metabolism	Xenobiotics Biodegradation and Metabolism	00361 Chlorocyclohexane and chlorobenzene degradation [PATH:ko00361]	haloacetate dehalogenase [EC:3.8.1.3]	K01561	0	0	0	2	0
Metabolism	Xenobiotics Biodegradation and Metabolism	00362 Benzoate degradation [PATH:ko00362]	2-hydroxycyclohexanecarboxyl-CoA dehydrogenase [EC:1.1.1.-]	K07535	0	0	1	0	0
Metabolism	Xenobiotics Biodegradation and Metabolism	00362 Benzoate degradation [PATH:ko00362]	3-carboxy-cis,cis-muconate cycloisomerase [EC:5.5.1.2]	K01857	0	0	0	2	4
Metabolism	Xenobiotics Biodegradation and Metabolism	00362 Benzoate degradation [PATH:ko00362]	3-hydroxybutyryl-CoA dehydrogenase [EC:1.1.1.157]	K00074	0	0	0	5	1
Metabolism	Xenobiotics Biodegradation and Metabolism	00362 Benzoate degradation [PATH:ko00362]	3-oxoadipate CoA-transferase, alpha subunit [EC:2.8.3.6]	K01031	0	0	0	0	1
Metabolism	Xenobiotics Biodegradation and Metabolism	00362 Benzoate degradation [PATH:ko00362]	3-oxoadipyl-CoA thiolase [EC:2.3.1.174]	K07823	0	0	0	0	1
Metabolism	Xenobiotics Biodegradation and Metabolism	00362 Benzoate degradation [PATH:ko00362]	4-carboxy-2-hydroxymuconate-6-semialdehyde dehydrogenase	K10219	0	0	0	1	0
Metabolism	Xenobiotics Biodegradation and Metabolism	00362 Benzoate degradation [PATH:ko00362]	4-carboxymuconolactone decarboxylase [EC:4.1.1.44]	K01607	0	0	0	1	3
Metabolism	Xenobiotics Biodegradation and Metabolism	00362 Benzoate degradation [PATH:ko00362]	4-hydroxy-4-methyl-2-oxoglutarate aldolase [EC:4.1.3.17]	K10218	0	0	0	0	1

Level 1	Level 2	Level 3	Function	KEGG ID	Sequence Reads				
					Day17	Day24	Day31	Day38	Bulk Soil
Metabolism	Xenobiotics Biodegradation and Metabolism	00362 Benzoate degradation [PATH:ko00362]	6-oxo-cyclohex-1-ene-carbonyl-CoA hydrolase [EC:3.7.1.-]	K07539	0	0	1	0	0
Metabolism	Xenobiotics Biodegradation and Metabolism	00362 Benzoate degradation [PATH:ko00362]	cyclohexa-1,5-dienecarbonyl-CoA hydratase [EC:4.2.1.100]	K07537	0	0	1	0	0
Metabolism	Xenobiotics Biodegradation and Metabolism	00362 Benzoate degradation [PATH:ko00362]	glutaryl-CoA dehydrogenase [EC:1.3.99.7]	K00252	3	1	0	4	0
Metabolism	Xenobiotics Biodegradation and Metabolism	00362 Benzoate degradation [PATH:ko00362]	p-hydroxybenzoate 3-monooxygenase [EC:1.14.13.2]	K00481	0	0	2	2	3
Metabolism	Xenobiotics Biodegradation and Metabolism	00621 Dioxin degradation [PATH:ko00621]	acetaldehyde dehydrogenase [EC:1.2.1.10]	K04073	0	0	1	0	0
Metabolism	Xenobiotics Biodegradation and Metabolism	00621 Dioxin degradation [PATH:ko00621]	biphenyl-2,3-diol 1,2-dioxygenase [EC:1.13.11.39]	K00462	0	1	0	0	0
Metabolism	Xenobiotics Biodegradation and Metabolism	00622 Xylene degradation [PATH:ko00622]	benzaldehyde dehydrogenase (NAD) [EC:1.2.1.28]	K00141	0	2	0	0	0
Metabolism	Xenobiotics Biodegradation and Metabolism	00623 Toluene degradation [PATH:ko00623]	carboxymethylenebutenolidase [EC:3.1.1.45]	K01061	8	1	5	2	5
Metabolism	Xenobiotics Biodegradation and Metabolism	00623 Toluene degradation [PATH:ko00623]	catechol 1,2-dioxygenase [EC:1.13.11.1]	K03381	0	0	0	0	1

Level 1	Level 2	Level 3	Function	KEGG ID	Sequence Reads				
					Day17	Day24	Day31	Day38	Bulk Soil
Metabolism	Xenobiotics Biodegradation and Metabolism	00623 Toluene degradation [PATH:ko00623]	maleylacetate reductase [EC:1.3.1.32]	K00217	0	0	0	1	0
Metabolism	Xenobiotics Biodegradation and Metabolism	00623 Toluene degradation [PATH:ko00623]	phenol 2-monooxygenase [EC:1.14.13.7]	K03380	0	0	1	1	1
Metabolism	Xenobiotics Biodegradation and Metabolism	00623 Toluene degradation [PATH:ko00623]	succinate dehydrogenase cytochrome b-556 subunit	K00241	3	0	1	0	0
Metabolism	Xenobiotics Biodegradation and Metabolism	00623 Toluene degradation [PATH:ko00623]	succinate dehydrogenase flavoprotein subunit [EC:1.3.99.1]	K00239	5	4	0	6	10
Metabolism	Xenobiotics Biodegradation and Metabolism	00623 Toluene degradation [PATH:ko00623]	succinate dehydrogenase hydrophobic membrane anchor protein	K00242	0	0	1	0	1
Metabolism	Xenobiotics Biodegradation and Metabolism	00623 Toluene degradation [PATH:ko00623]	succinate dehydrogenase iron-sulfur protein [EC:1.3.99.1]	K00240	4	2	0	0	0
Metabolism	Xenobiotics Biodegradation and Metabolism	00624 Polycyclic aromatic hydrocarbon degradation [PATH:ko00624]	[EC:1.14.-.-]	K00517	0	0	5	0	0
Metabolism	Xenobiotics Biodegradation and Metabolism	00624 Polycyclic aromatic hydrocarbon degradation [PATH:ko00624]	[EC:1.14.13.-]	K00492	0	0	0	1	3
Metabolism	Xenobiotics Biodegradation and Metabolism	00624 Polycyclic aromatic hydrocarbon degradation [PATH:ko00624]	[EC:1.2.1.-]	K00155	0	0	0	2	2

Level 1	Level 2	Level 3	Function	KEGG ID	Sequence Reads				
					Day17	Day24	Day31	Day38	Bulk Soil
Metabolism	Xenobiotics Biodegradation and Metabolism	00624 Polycyclic aromatic hydrocarbon degradation [PATH:ko00624]	[EC:2.1.1.-]	K00599	0	0	4	0	0
Metabolism	Xenobiotics Biodegradation and Metabolism	00624 Polycyclic aromatic hydrocarbon degradation [PATH:ko00624]	protocatechuate 3,4-dioxygenase, beta subunit [EC:1.13.11.3]	K00449	1	0	0	0	0
Metabolism	Xenobiotics Biodegradation and Metabolism	00624 Polycyclic aromatic hydrocarbon degradation [PATH:ko00624]	protocatechuate 4,5-dioxygenase [EC:1.13.11.8]	K04099	0	0	0	1	0
Metabolism	Xenobiotics Biodegradation and Metabolism	00624 Polycyclic aromatic hydrocarbon degradation [PATH:ko00624]	salicylate hydroxylase [EC:1.14.13.1]	K00480	0	1	0	2	0
Metabolism	Xenobiotics Biodegradation and Metabolism	00625 Chloroalkane and chloroalkene degradation [PATH:ko00625]	alcohol dehydrogenase (cytochrome c) [EC:1.1.2.8]	K00114	3	0	0	0	0
Metabolism	Xenobiotics Biodegradation and Metabolism	00625 Chloroalkane and chloroalkene degradation [PATH:ko00625]	aldehyde dehydrogenase (NAD+) [EC:1.2.1.3]	K00128	0	7	9	15	0
Metabolism	Xenobiotics Biodegradation and Metabolism	00625 Chloroalkane and chloroalkene degradation [PATH:ko00625]	glutathione-independent formaldehyde dehydrogenase	K00148	0	0	3	0	0
Metabolism	Xenobiotics Biodegradation and Metabolism	00625 Chloroalkane and chloroalkene degradation [PATH:ko00625]	methanol dehydrogenase (cytochrome c) subunit 1 [EC:1.1.2.7]	K14028	0	0	0	1	0
Metabolism	Xenobiotics Biodegradation and Metabolism	00625 Chloroalkane and chloroalkene degradation [PATH:ko00625]	nitrogenase iron protein NifH [EC:1.18.6.1]	K02588	0	0	0	1	0

Level 1	Level 2	Level 3	Function	KEGG ID	Sequence Reads				
					Day17	Day24	Day31	Day38	Bulk Soil
Metabolism	Xenobiotics Biodegradation and Metabolism	00625 Chloroalkane and chloroalkene degradation [PATH:ko00625]	nitrogenase molybdenum-iron protein alpha chain	K02586	1	0	0	0	0
Metabolism	Xenobiotics Biodegradation and Metabolism	00626 Naphthalene degradation [PATH:ko00626]	[EC:1.3.99.-]	K00257	10	10	8	15	18
Metabolism	Xenobiotics Biodegradation and Metabolism	00626 Naphthalene degradation [PATH:ko00626]	[EC:2.3.1.-]	K00680	0	0	1	0	0
Metabolism	Xenobiotics Biodegradation and Metabolism	00626 Naphthalene degradation [PATH:ko00626]	[EC:4.2.1.-]	K01726	0	1	0	0	0
Metabolism	Xenobiotics Biodegradation and Metabolism	00626 Naphthalene degradation [PATH:ko00626]	alcohol dehydrogenase [EC:1.1.1.1]	K13954	1	0	0	0	0
Metabolism	Xenobiotics Biodegradation and Metabolism	00627 Aminobenzoate degradation [PATH:ko00627]	2-aminobenzoate-CoA ligase [EC:6.2.1.32]	K08295	0	0	2	7	0
Metabolism	Xenobiotics Biodegradation and Metabolism	00627 Aminobenzoate degradation [PATH:ko00627]	4-hydroxybenzoate-CoA ligase [EC:6.2.1.27]	K04105	0	0	1	0	0
Metabolism	Xenobiotics Biodegradation and Metabolism	00627 Aminobenzoate degradation [PATH:ko00627]	4-hydroxybenzoyl-CoA reductase subunit gamma [EC:1.3.99.20]	K04107	0	0	0	1	0
Metabolism	Xenobiotics Biodegradation and Metabolism	00627 Aminobenzoate degradation [PATH:ko00627]	anthraniloyl-CoA monooxygenase [EC:1.14.13.40]	K09461	0	0	5	6	0

Level 1	Level 2	Level 3	Function	KEGG ID	Sequence Reads				
					Day17	Day24	Day31	Day38	Bulk Soil
Metabolism	Xenobiotics Biodegradation and Metabolism	00627 Aminobenzoate degradation [PATH:ko00627]	benzoylformate decarboxylase [EC:4.1.1.7]	K01576	1	0	0	0	0
Metabolism	Xenobiotics Biodegradation and Metabolism	00627 Aminobenzoate degradation [PATH:ko00627]	cytochrome P450 / NADPH-cytochrome P450 reductase	K14338	0	0	1	0	1
Metabolism	Xenobiotics Biodegradation and Metabolism	00627 Aminobenzoate degradation [PATH:ko00627]	unspecific monooxygenase [EC:1.14.14.1]	K00493	1	0	0	0	0
Metabolism	Xenobiotics Biodegradation and Metabolism	00627 Aminobenzoate degradation [PATH:ko00627]	vanillate monooxygenase [EC:1.14.13.82]	K03862	0	0	1	3	1
Metabolism	Xenobiotics Biodegradation and Metabolism	00633 Nitrotoluene degradation [PATH:ko00633]	carbon-monoxide dehydrogenase large subunit [EC:1.2.99.2]	K03520	2	0	0	0	0
Metabolism	Xenobiotics Biodegradation and Metabolism	00633 Nitrotoluene degradation [PATH:ko00633]	carbon-monoxide dehydrogenase medium subunit [EC:1.2.99.2]	K03519	0	0	0	0	5
Metabolism	Xenobiotics Biodegradation and Metabolism	00633 Nitrotoluene degradation [PATH:ko00633]	carbon-monoxide dehydrogenase small subunit [EC:1.2.99.2]	K03518	2	0	0	0	0
Metabolism	Xenobiotics Biodegradation and Metabolism	00633 Nitrotoluene degradation [PATH:ko00633]	hydrogenase large subunit [EC:1.12.99.6]	K06281	1	1	0	0	4
Metabolism	Xenobiotics Biodegradation and Metabolism	00633 Nitrotoluene degradation [PATH:ko00633]	hydrogenase small subunit [EC:1.12.99.6]	K06282	0	1	0	1	0

Level 1	Level 2	Level 3	Function	KEGG ID	Sequence Reads				
					Day17	Day24	Day31	Day38	Bulk Soil
Metabolism	Xenobiotics Biodegradation and Metabolism	00633 Nitrotoluene degradation [PATH:ko00633]	pyruvate ferredoxin oxidoreductase, alpha subunit	K00169	0	0	0	2	0
Metabolism	Xenobiotics Biodegradation and Metabolism	00633 Nitrotoluene degradation [PATH:ko00633]	pyruvate ferredoxin oxidoreductase, beta subunit	K00170	0	2	0	0	0
Metabolism	Xenobiotics Biodegradation and Metabolism	00642 Ethylbenzene degradation [PATH:ko00642]	acetyl-CoA acyltransferase [EC:2.3.1.16]	K00632	0	2	0	1	2
Metabolism	Xenobiotics Biodegradation and Metabolism	00643 Styrene degradation [PATH:ko00643]	glutaconate CoA-transferase, subunit B [EC:2.8.3.12]	K01040	0	1	0	0	0
Metabolism	Xenobiotics Biodegradation and Metabolism	00643 Styrene degradation [PATH:ko00643]	homogentisate 1,2-dioxygenase [EC:1.13.11.5]	K00451	0	1	0	0	0
Metabolism	Xenobiotics Biodegradation and Metabolism	00643 Styrene degradation [PATH:ko00643]	nitrile hydratase [EC:4.2.1.84]	K01721	2	0	0	0	0
Metabolism	Xenobiotics Biodegradation and Metabolism	00643 Styrene degradation [PATH:ko00643]	propionate CoA-transferase [EC:2.8.3.1]	K01026	0	2	0	0	0
Metabolism	Xenobiotics Biodegradation and Metabolism	00791 Atrazine degradation [PATH:ko00791]	[EC:3.5.4.-]	K01500	0	1	0	0	3
Metabolism	Xenobiotics Biodegradation and Metabolism	00791 Atrazine degradation [PATH:ko00791]	allophanate hydrolase [EC:3.5.1.54]	K01457	1	3	0	0	0

Level 1	Level 2	Level 3	Function	KEGG ID	Sequence Reads				
					Day17	Day24	Day31	Day38	Bulk Soil
Metabolism	Xenobiotics Biodegradation and Metabolism	00791 Atrazine degradation [PATH:ko00791]	cyanuric acid amidohydrolase [EC:3.5.2.15]	K03383	0	0	0	1	0
Metabolism	Xenobiotics Biodegradation and Metabolism	00791 Atrazine degradation [PATH:ko00791]	hydroxyatrazine ethylaminohydrolase [EC:3.5.99.3]	K03382	0	0	1	0	0
Metabolism	Xenobiotics Biodegradation and Metabolism	00791 Atrazine degradation [PATH:ko00791]	urease subunit gamma [EC:3.5.1.5]	K01430	0	0	0	0	1
Metabolism	Xenobiotics Biodegradation and Metabolism	00930 Caprolactam degradation [PATH:ko00930]	3-hydroxyacyl-CoA dehydrogenase / enoyl-CoA hydratase /	K01782	1	0	2	1	5
Metabolism	Xenobiotics Biodegradation and Metabolism	00930 Caprolactam degradation [PATH:ko00930]	acyl-CoA dehydrogenase [EC:1.3.99.-]	K06446	0	0	1	2	2
Metabolism	Xenobiotics Biodegradation and Metabolism	00930 Caprolactam degradation [PATH:ko00930]	enoyl-CoA hydratase [EC:4.2.1.17]	K01692	7	19	14	15	0
Metabolism	Xenobiotics Biodegradation and Metabolism	00930 Caprolactam degradation [PATH:ko00930]	gluconolactonase [EC:3.1.1.17]	K01053	4	0	5	0	2
Metabolism	Xenobiotics Biodegradation and Metabolism	00980 Metabolism of xenobiotics by cytochrome P450 [PATH:ko00980]	microsomal epoxide hydrolase [EC:3.3.2.9]	K01253	1	0	0	0	1
Metabolism	Xenobiotics Biodegradation and Metabolism	00982 Drug metabolism - cytochrome P450 [PATH:ko00982]	alcohol dehydrogenase [EC:1.1.1.1]	K00001	0	0	0	6	5

Level 1	Level 2	Level 3	Function	KEGG ID	Sequence Reads				
					Day17	Day24	Day31	Day38	Bulk Soil
Metabolism	Xenobiotics Biodegradation and Metabolism	00982 Drug metabolism - cytochrome P450 [PATH:ko00982]	glutathione S-transferase [EC:2.5.1.18]	K00799	5	0	0	0	0
Metabolism	Xenobiotics Biodegradation and Metabolism	00982 Drug metabolism - cytochrome P450 [PATH:ko00982]	S-(hydroxymethyl)glutathione dehydrogenase / alcohol	K00121	0	0	2	0	3
Metabolism	Xenobiotics Biodegradation and Metabolism	00983 Drug metabolism - other enzymes [PATH:ko00983]	dihydropyrimidinase [EC:3.5.2.2]	K01464	0	0	0	0	3
Metabolism	Xenobiotics Biodegradation and Metabolism	00983 Drug metabolism - other enzymes [PATH:ko00983]	GMP synthase (glutamine-hydrolysing) [EC:6.3.5.2]	K01951	3	5	0	2	7
Metabolism	Xenobiotics Biodegradation and Metabolism	00983 Drug metabolism - other enzymes [PATH:ko00983]	IMP dehydrogenase [EC:1.1.1.205]	K00088	4	3	7	3	6
Metabolism	Xenobiotics Biodegradation and Metabolism	00983 Drug metabolism - other enzymes [PATH:ko00983]	thymidine kinase [EC:2.7.1.21]	K00857	0	2	1	1	0
Organismal Systems	Digestive System	04972 Pancreatic secretion [PATH:ko04972]	Ca2+ transporting ATPase, plasma membrane [EC:3.6.3.8]	K05850	0	0	2	0	0
Organismal Systems	Digestive System	04974 Protein digestion and absorption [PATH:ko04974]	lysosomal Pro-X carboxypeptidase [EC:3.4.16.2]	K01285	0	1	0	0	0
Organismal Systems	Digestive System	04976 Bile secretion [PATH:ko04976]	hydroxymethylglutaryl-CoA reductase (NADPH) [EC:1.1.1.34]	K00021	0	1	0	0	0
Organismal Systems	Endocrine System	03320 PPAR signaling pathway [PATH:ko03320]	acyl-CoA dehydrogenase [EC:1.3.99.3]	K00249	0	13	10	13	0
Organismal Systems	Endocrine System	03320 PPAR signaling pathway [PATH:ko03320]	acyl-CoA oxidase [EC:1.3.3.6]	K00232	1	0	0	0	0

Level 1	Level 2	Level 3	Function	KEGG ID	Sequence Reads				
					Day17	Day24	Day31	Day38	Bulk Soil
Organismal Systems	Endocrine System	03320 PPAR signaling pathway [PATH:ko03320]	long-chain acyl-CoA synthetase [EC:6.2.1.3]	K01897	10	0	0	8	0
Organismal Systems	Endocrine System	03320 PPAR signaling pathway [PATH:ko03320]	stearoyl-CoA desaturase (delta-9 desaturase) [EC:1.14.19.1]	K00507	3	0	0	1	0
Organismal Systems	Endocrine System	03320 PPAR signaling pathway [PATH:ko03320]	sterol carrier protein 2 [EC:2.3.1.176]	K08764	2	0	0	0	0
Organismal Systems	Endocrine System	03320 PPAR signaling pathway [PATH:ko03320]	ubiquitin C	K08770	0	0	2	1	0
Organismal Systems	Endocrine System	04910 Insulin signaling pathway [PATH:ko04910]	flotillin	K07192	0	2	0	0	1
Organismal Systems	Endocrine System	04910 Insulin signaling pathway [PATH:ko04910]	fructose-1,6-bisphosphatase I [EC:3.1.3.11]	K03841	0	1	1	1	0
Organismal Systems	Endocrine System	04910 Insulin signaling pathway [PATH:ko04910]	phosphorylase kinase alpha/beta subunit	K07190	1	0	0	0	0
Organismal Systems	Endocrine System	04910 Insulin signaling pathway [PATH:ko04910]	small subunit ribosomal protein S6e	K02991	0	0	1	0	0
Organismal Systems	Endocrine System	04910 Insulin signaling pathway [PATH:ko04910]	starch phosphorylase [EC:2.4.1.1]	K00688	4	5	3	8	0
Organismal Systems	Environmental Adaptation	04626 Plant-pathogen interaction [PATH:ko04626]	elongation factor EF-Tu [EC:3.6.5.3]	K02358	39	9	18	23	5
Organismal Systems	Environmental Adaptation	04626 Plant-pathogen interaction [PATH:ko04626]	flagellin	K02406	38	2	0	0	0
Organismal Systems	Environmental Adaptation	04626 Plant-pathogen interaction [PATH:ko04626]	glycerol kinase [EC:2.7.1.30]	K00864	0	5	4	0	0
Organismal Systems	Environmental Adaptation	04710 Circadian rhythm - mammal	cullin 1	K03347	1	0	0	0	0

Level 1	Level 2	Level 3	Function	KEGG ID	Sequence Reads				
					Day17	Day24	Day31	Day38	Bulk Soil
		[PATH:ko04710]							
Organismal Systems	Environmental Adaptation	04710 Circadian rhythm - mammal [PATH:ko04710]	S-phase kinase-associated protein 1	K03094	0	0	1	0	0
Organismal Systems	Environmental Adaptation	04712 Circadian rhythm - plant [PATH:ko04712]	casein kinase 2, alpha polypeptide [EC:2.7.11.1]	K03097	1	0	1	0	0
Organismal Systems	Environmental Adaptation	04712 Circadian rhythm - plant [PATH:ko04712]	chalcone synthase [EC:2.3.1.74]	K00660	0	0	1	0	0
Organismal Systems	Environmental Adaptation	04712 Circadian rhythm - plant [PATH:ko04712]	phytochrome A	K12120	1	0	0	0	0
Organismal Systems	Excretory System	04962 Vasopressin-regulated water reabsorption [PATH:ko04962]	dynein heavy chain 1, cytosolic	K10413	2	0	0	0	0
Organismal Systems	Excretory System	04964 Proximal tubule bicarbonate reclamation [PATH:ko04964]	glutaminase [EC:3.5.1.2]	K01425	0	0	2	2	0
Organismal Systems	Excretory System	04964 Proximal tubule bicarbonate reclamation [PATH:ko04964]	phosphoenolpyruvate carboxykinase (GTP) [EC:4.1.1.32]	K01596	6	3	0	0	4
Organismal Systems	Immune System	04612 Antigen processing and presentation [PATH:ko04612]	calnexin	K08054	0	0	1	0	0
Organismal Systems	Immune System	04612 Antigen processing and presentation [PATH:ko04612]	cathepsin B [EC:3.4.22.1]	K01363	0	1	0	0	0
Organismal Systems	Immune System	04622 RIG-I-like receptor signaling pathway [PATH:ko04622]	ATP-dependent RNA helicase [EC:3.6.4.13]	K11594	1	0	0	0	0
Organismal Systems	Immune System	04623 Cytosolic DNA-sensing pathway	DNA-directed RNA polymerase III subunit C2 [EC:2.7.7.6]	K03021	0	2	0	0	0

Level 1	Level 2	Level 3	Function	KEGG ID	Sequence Reads				
					Day17	Day24	Day31	Day38	Bulk Soil
		[PATH:ko04623]							
Organismal Systems	Nervous System	04720 Long-term potentiation [PATH:ko04720]	protein phosphatase 1, catalytic subunit [EC:3.1.3.16]	K06269	0	0	1	0	0
Organismal Systems	Nervous System	04722 Neurotrophin signaling pathway [PATH:ko04722]	tyrosine 3-monooxygenase/tryptophan 5-monooxygenase	K06630	1	0	0	0	0
