# Supplementary figures and images for: Root Exudation of Phytochemicals in Arabidopsis Follows Specific Patterns That Are Developmentally Programmed and Correlate with Soil Microbial Functions
Source: PLoS One. 2013 Feb 1;8(2):e55731. doi: 10.1371/journal.pone.0055731 (PMC3562227; doi:10.1371/journal.pone.0055731)

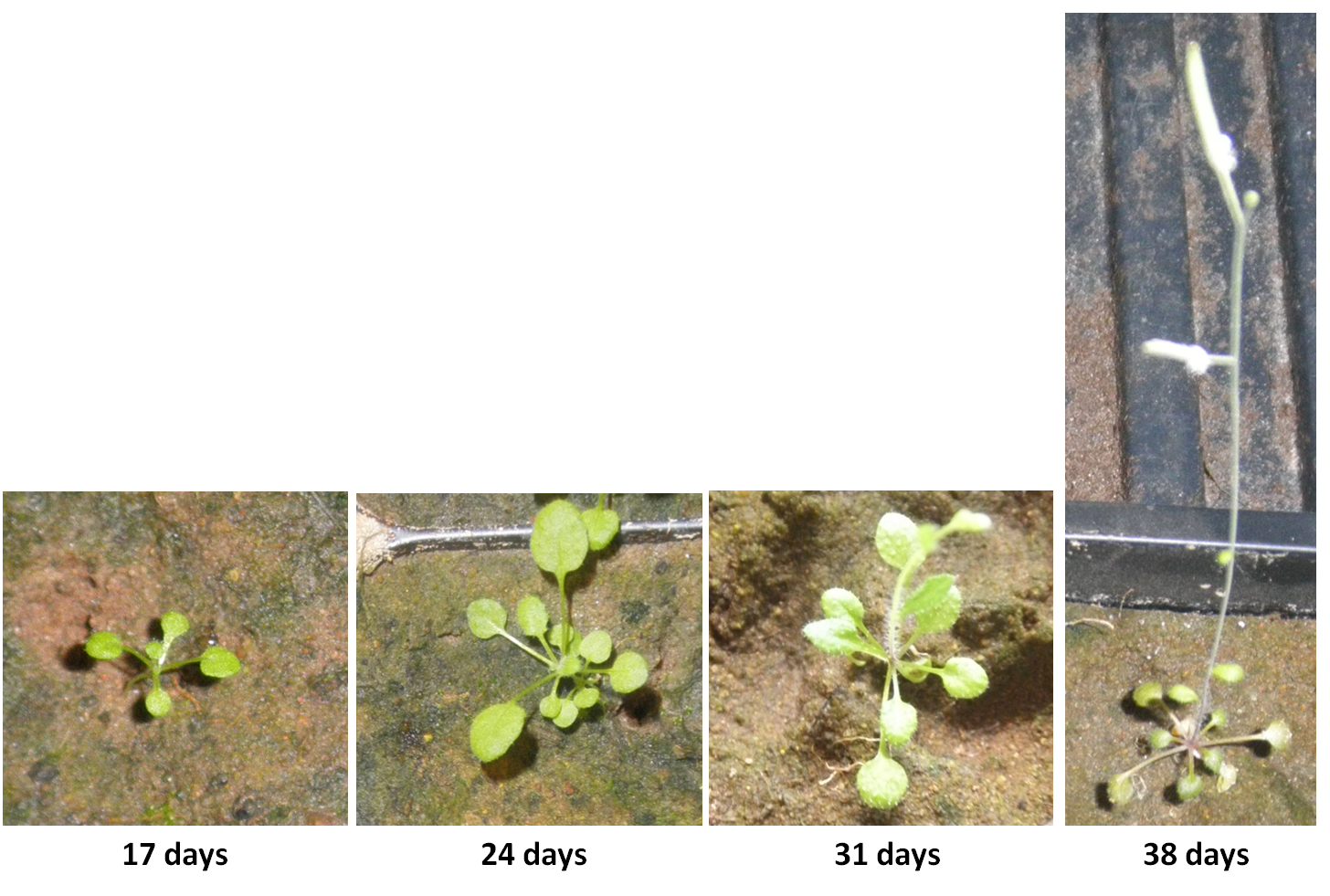

Supplement: Figure S1 — Soil grown Arabidopsis thaliana Col-0 at each plant developmental stage (17, 24, 31 and 38 days). (TIF) [file pone.0055731.s008.tif]

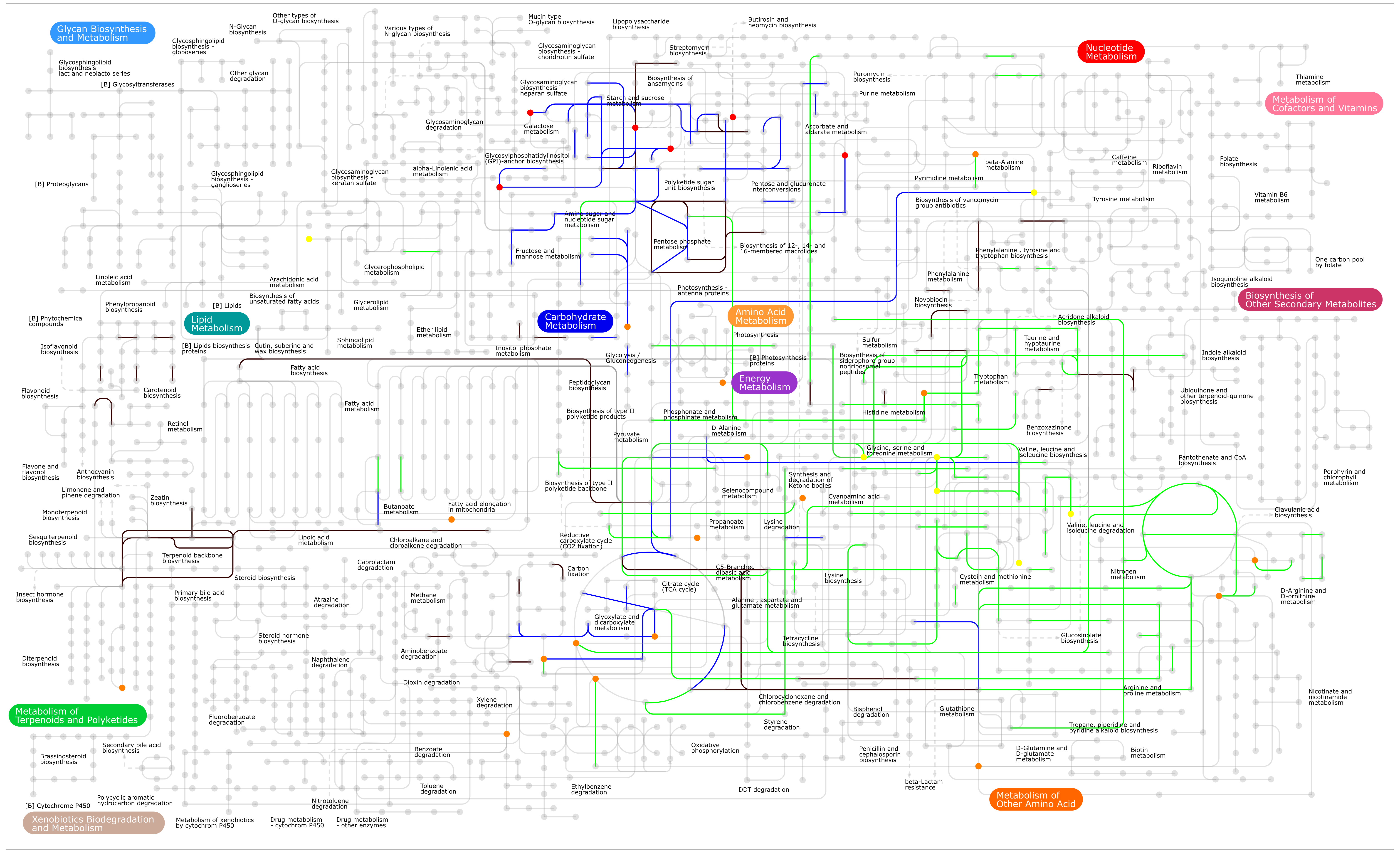

Supplement: Figure S2 — iPATH 2 KEGG Map exhibiting the functional genes involved in Metabolism with the identified root exudate compounds. Blue lines: functional genes involved in Carbohydrate Metabolism; Green lines: functional genes involved in Amino Acid Metabolism; Brown lines: functional genes involved in the Metabolism of Secondary Metabolites which includes the Biosynthesis of Other Secondary Metabolites and Metabolism of Terpenoids and Polyketides; Red dots: root exudate compounds classified as sugars; Yellow dots: root exudate compounds classified as amino acids; Orange dots: root exudate compounds classified as phenolics. (PNG) [file pone.0055731.s009.png]
